# Melanopsin elevates locomotor activity during the wake state of the diurnal zebrafish

**DOI:** 10.15252/embr.202051528

**Published:** 2022-03-01

**Authors:** Marcus P S Dekens, Bruno M Fontinha, Miguel Gallach, Sandra Pflügler, Kristin Tessmar‐Raible

**Affiliations:** ^1^ Max Perutz Laboratory Centre for Molecular Biology University of Vienna and Medical University of Vienna Vienna Austria; ^2^ Max Perutz Laboratory Centre for Integrative Bioinformatics University of Vienna and Medical University of Vienna Vienna Austria; ^3^ Research Platform “Marine Rhythms of Life” University of Vienna Vienna Austria; ^4^ Present address: iLabSystems Vienna Austria

**Keywords:** behavioural genetics, melatonin, neurogenetics, photobiology, pineal, Neuroscience, Signal Transduction

## Abstract

Mammalian and fish pineals play a key role in adapting behaviour to the ambient light conditions through the release of melatonin. In mice, light inhibits nocturnal locomotor activity via the non‐visual photoreceptor Melanopsin. In contrast to the extensively studied function of Melanopsin in the indirect regulation of the rodent pineal, its role in the intrinsically photosensitive zebrafish pineal has not been elucidated. Therefore, it is not evident if the light signalling mechanism is conserved between distant vertebrates, and how Melanopsin could affect diurnal behaviour. A double knockout of *melanopsins* (*opn4.1*‐*opn4xb*) was generated in the diurnal zebrafish, which manifests attenuated locomotor activity during the wake state. Transcriptome sequencing gave insight into pathways downstream of Melanopsin, implying that sustained repression of the melatonin pathway is required to elevate locomotor activity during the diurnal wake state. Moreover, we show that light induces locomotor activity during the diurnal wake state in an intensity‐dependent manner. These observations suggest a common Melanopsin‐driven mechanism between zebrafish and mammals, while the diurnal and nocturnal chronotypes are inversely regulated downstream of melatonin.

## Graphical abstract

This study proposes a correlation between the number of detected photons and locomotor activity in the diurnal wake state in zebrafish. Light elevates the activity level through a Melanopsin‐mediated repression of the melatonin pathway.

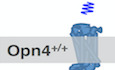

## Introduction

To adapt to the ambient light conditions, activity is regulated directly by light and indirectly through the circadian clock (Dunlap *et al*, 2004), which anticipates daily recurring events. In nocturnal mice, the non‐visual photoreceptor Melanopsin (OPN4) functions in circadian clock entrainment (Panda *et al*, 2002; Ruby *et al*, 2002) and in the direct induction of sleep by light (Mrosovsky & Hattar, 2003; Lupi *et al*, 2008; Tsai *et al*, 2009). In contrast to nocturnal mice, where *Opn4* is solely expressed in the intrinsically photoreceptive retinal ganglion cells (ipRGCs) (Hatori & Panda, 2010), several diurnal species also express *Opn4* in the brain. *Opn4* is transcribed in many domains of the human brain (Hawrylycz *et al*, 2012; Nissilä *et al*, 2017), the zebrafish larval brain (Davies *et al*, 2011; Matos‐Cruz *et al*, 2011), the chicken pineal (Holthues *et al*, 2004; Bailey & Cassone, 2005; Chaurasia *et al*, 2005) and in the pineal of the Atlantic halibut (Eilertsen *et al*, 2014). In vertebrates, the pineal gland or epiphysis cerebri plays a key role in synchronising behaviour and physiology to the environmental light–dark (LD) cycles through the rhythmic production of the indolamine melatonin (Sapède & Cau, 2013). The melatonin level reaches its peak during the night in both diurnal and nocturnal animals (Challet, 2007), thus melatonin is a dark phase indicator. In mice, ipRGCs provide the central clock located in the suprachiasmatic nucleus (SCN) with photic information, which in succession regulates the pineal via the paraventricular nucleus (PVN) (Simonneaux & Ribelayga, 2003). In contrast to the mammalian pineal, which receives indirect photic input through sympathetic norepinephrine (NE) innervation (Simonneaux & Ribelayga, 2003), the pineals of many fish, amphibians, reptiles and birds display all characteristics of a photoreceptive organ (Korf *et al*, 1998; Ziv *et al*, 2007; Sapède & Cau, 2013; Ben‐Moshe Livne *et al*, 2016; Bertolesi & McFarlane, 2018) and generate endogenous endocrine rhythms, combining input and output functions in the photoreceptor cell (Falcon *et al*, 2007). Moreover, NE does not play a role in regulating melatonin synthesis in zebrafish (Cahill, 1997).

Melatonin is synthesised by four enzymes: Tryptophan hydroxylase (TPH) catalyses the oxidation of the amino acid tryptophan to 5‐hydroxy‐L‐tryptophan followed by the removal of a carboxyl group by Dopa decarboxylase (DDC) to produce serotonin. Arylalkylamine N‐acetyltransferase (AANAT) adds an acetyl group to produce N‐acetyl‐serotonin, which is then methylated by Acetyl‐serotonin O‐methyltransferase (ASMT) into melatonin. The nightly release of NE in the rodent pineal activates the cAMP pathway resulting in the phosphorylation of cAMP response element‐binding protein (CREB), which induces *Aanat* transcription (Rohde *et al*, 2014a). Homeodomain transcription factors control pinealocyte specific gene expression by binding to highly conserved pineal regulatory elements (PIRE) (Li *et al*, 1998), thereby permitting pCREB to regulate these genes through CRE elements. The transcription factor Cone‐rod homeodomain (CRX) induces *Aanat* (Li *et al*, 1998; Rohde *et al*, 2014b), *Tph1* and *Asmt* (Rohde *et al*, 2019) transcription in the mature rat pineal. Furthermore, Orthodenticle homeobox 2 (OTX2) transactivates *Crx* (Nishida *et al*, 2003; Rohde *et al*, 2019) and induces *Tph1*, *Aanat* and *Asmt* (Rohde *et al*, 2019), and LIM homeobox 4 (LHX4) induces *Aanat* (Hertz *et al*, 2020). In zebrafish, the homeodomain transcription factor Otx5 has been reported to regulate *aanat2* (Gamse *et al*, 2002). As the *aanat2* promoter contains CRE elements (Falcon *et al*, 2007), Creb could also play a role in regulating the zebrafish melatonin pathway. The rate of melatonin synthesis mainly depends on AANAT (Klein & Weller, 1970; Klein *et al*, 1997). However, the melatonin level is effectively raised by inducing multiple components of the melatonin pathway (Liu & Borjigin, 2005; Rohde *et al*, 2019).

Melatonin has been demonstrated to promote sleep in diurnal vertebrates ranging from zebrafish (Zhdanova *et al*, 2001) to humans (Dollins *et al*, 1994; Mintz *et al*, 1998; Zhdanova *et al*, 2002; Brzezinski *et al*, 2005; Lok *et al*, 2019), and a light pulse in the night suppresses melatonin production with the strongest effect at the wavelength where Opn4/OPN4 has its absorption optimum in both zebrafish (Ziv *et al*, 2007) and humans (Lewy *et al*, 1980; Czeisler *et al*, 1995; Cajochen *et al*, 2000; Lockley *et al*, 2003). The reduction in the melatonin level is proportional to the intensity of the light pulse (Max & Menaker, 1992; Zachmann *et al*, 1992b; Bolliet *et al*, 1995) implying regulation by a light intensity detector. Therefore, OPN4 is a primary candidate in this process (Wong *et al*, 2005; Mure *et al*, 2016). Several human studies have shown that bright light during daytime increases activity and vigilance (Cajochen *et al*, 2000; Phipps‐Nelson *et al*, 2003; Lockley *et al*, 2006; Smolders *et al*, 2012; Smolders & de Kort, 2014; Knaier *et al*, 2016). These data suggest that in diurnal vertebrates, suppression of melatonin production by light may also play a role in regulating activity levels during the wake state. To investigate how zebrafish adapt to ambient light conditions and the role of Opn4 in this process, a double knockout (dko) was generated of *opn4.1* and *opn4xb* which are coexpressed in the pineal. This study implies that locomotor activity during the wake state is regulated by an Melanopsin‐driven mechanism that is common between mammals and zebrafish despite the differences in light input and chronotypes. The Opn4‐dependent transcriptome also suggests that Opn4 influences the immune system and the cell cycle in addition to the control it exerts over genes that encode melatonin synthesis enzymes. These findings alter the perspective of a photoreceptor that has until now solely been associated with behaviour to one that adapts diverse functions to the environment.

## Results

### *opn4* expression in the brain and knockout strategy

**Figure EV1:**
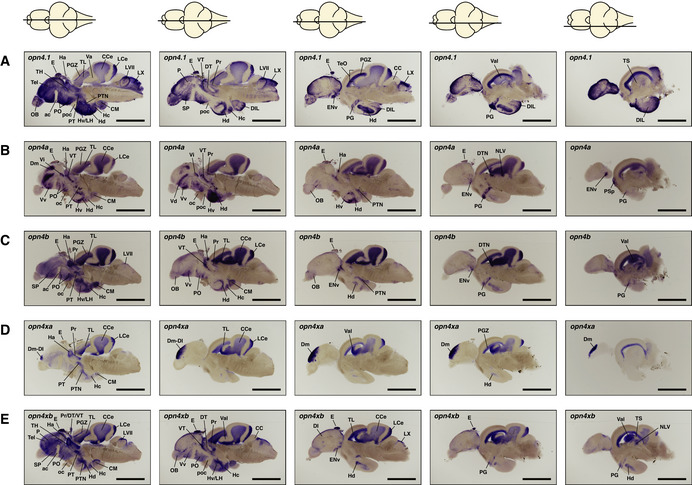
*ishs* on brain sections reveal broad expression of all five zebrafish *melanopsins* in the mature brain (A–E) *opn4.1* (A) *opn4a* (B) *opn4b* (C) *opn4xa* (D) and *opn4xb* (E). A horizontal line through a representation of the brain, above the section, indicates the location of the section within the brain. The names and abbreviations of all brain domains in which *opn4* is expressed are listed in Table EV1. Note that the duration of the colorimetric development of the *ishs* presented in this figure is not exactly the same. Scale bar: 1.0 mm.

**Figure 1:**
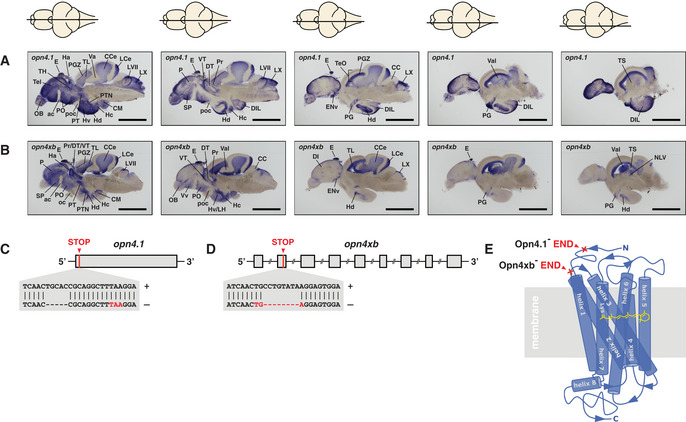
*melanopsin* expression in the brain and knockout strategy A, B
*In situs* on adult brain sections reveal that (A) *opn4.1* and (B) *opn4xb* are coexpressed in many brain domains including the epiphysis cerebri (indicated with E) or pineal. A horizontal line through a representation of the brain, above the section, indicates the location of the section within the brain. Scale bar: 1.0 mm. The brain domains in which the five *melanopsins* are expressed are presented in Fig EV1 and Table EV1.CThe TALEN genome‐editing technique was applied for site‐directed mutagenesis. A 5 bp deletion was introduced proximal to the start codon of *opn4.1*, resulting in a frame shift and stop codon. The grey box indicates the coding sequence, the red line indicates the location of the premature stop codon and the grey triangle dispays the wild‐type sequence aligned with the mutated sequence.DAn 8 bp deletion was introduced in the second exon (grey box) of the *opn4xb* gene resulting in a premature stop codon.EModel of the seven transmembrane G‐protein‐coupled photoreceptor shows the light absorbing molecule, the chromophore retinal (yellow), bound to helix 7 (blue). The red crosses indicate where the Melanopsins in the mutants are truncated: the mutated Opn4.1 has lost all transmembrane helices and the mutated Opn4xb ends after the second transmembrane helix. From the structure–function relationship of the photoreceptor, one can deduce that neither of the mutant Melanopsins is functional.

The expression patterns of all five *opn4* homologs were characterised in the adult zebrafish brain by *in situ* hybridisation (*ish*). The *opn4* genes show broad expression in the adult brain (Fig EV1A–E, Table EV1), which we documented in accordance with the neuroanatomical terminology established by Wullimann (Wullimann *et al*, 1996; Baeuml *et al*, 2019). *opn4.1* and *opn4xb* stand out as the *opn4* genes that are strongly expressed in the pineal (Fig 1A and B). To knockout *opn4.1* and *opn4xb* the transcription activator‐like effector nuclease (TALEN) genome editing technique (Cermak *et al*, 2011; Bedell *et al*, 2012) was applied. A premature stop codon was introduced close to the start codon in each gene (Fig 1C and D, Table 1). Opn4 is a seven transmembrane G‐protein coupled receptor with a light absorbing moiety, the chromophore retinal, bound to helix seven (Fig 1E). The mutated Opn4.1 has lost all transmembrane helices, and only the first and second transmembrane helices remain in the mutated Opn4xb. Thus, from the structure–function relationship, it can be deduced that neither of the mutated *opn4* genes encodes a functional photoreceptor. A homozygous double knockout (dko) was generated, as both Melanopsins are likely to have redundant functions given their conspicuous coexpression.

**Table 1:** Transcription activator‐like effector DNA‐binding domains.

Ensembl ID	Gene	Target Site	TAL1 DNA Binding Domain	TAL2 DNA Binding Domain
ENSDARG00000007553	*opn4.1*	proximal to START codon	tcactgtgcccctggagacat	ttcccaaagattccttaaag
ENSDARG00000103259	*opn4xb*	exon 2	tctccaatcttcttcatca	ttctccaaacatccact

### Expression profiling reveals pathways downstream of Opn4

**Figure 2:**
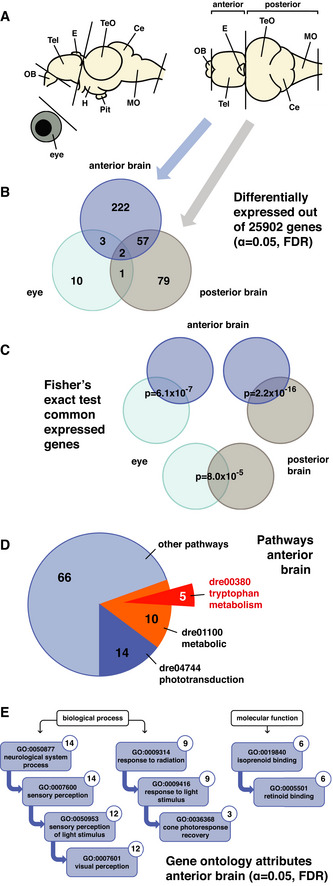
Expression profiling reveals pathways downstream of Opn4 Representation of the zebrafish adult brain shows the lateral and dorsal view with abbreviations for the following domains: olfactory bulb [OB], telencephalon [Tel], epiphysis cerebri or pineal [E], optic tectum [TeO], cerebellum [Ce], medulla oblongata [MO], hypothalamus [H] and hypophysis or pituitary [Pit]. Transcriptome sequencing was performed on cDNA from wild‐type and *opn4* dko eyes and brains, sampled in the light phase (ZT4‐6). The brains were separated in an anterior part, which includes part of the forebrain and the pineal, and a posterior part, which includes part of the forebrain and the whole mid‐ and hindbrain. The cut lines through the depicted brain indicate where the brain was partitioned and which parts were subjected to transcriptome sequencing. The blue (anterior brain) and grey (posterior brain) arrows connect the brain parts in (A) with the differentially expressed genes in (B).Venn diagram shows the number of differentially expressed genes in the *opn4* dko anterior brain (dark blue circle), posterior brain (grey circle) and eye (light blue circle). The cutoff for differential expression was set at the significance level of α = 0.05. Heat maps of all the differentially expressed genes are presented in Appendix Fig S1A–C. The number of differentially expressed genes that are shared between the data sets is indicated where the datasets overlap.Fisher's exact test shows that the shared differentially expressed genes between data sets are unlikely to be the result of coincidence. *P*‐values for the common differentially expressed genes are indicated where the datasets overlap. The large number of common differentially expressed genes between the data sets derived from the anterior and posterior brain parts is likely due to forebrain regions that are shared between these parts.Pie chart shows the number of differentially expressed genes in the *opn4* dko anterior brain that were assigned with KEGG software to phototransduction (dark blue), metabolic (orange) and other pathways. Of the metabolic pathway, 5 genes were assigned to the subcategory tryptophan metabolism (red), which encode all the enzymes that convert tryptophan into melatonin. Note that 189 genes were not assigned to a pathway.Ancestor charts show the gene ontology attributes to which the differentially expressed genes in the *opn4* dko anterior brain were assigned with FuncAssociate software (cutoff: α = 0.05). The number of genes assigned to an attribute is indicated in the upper right corner. All retrieved biological processes and molecular functions are associated with light, consistent with knockout of a photoreceptor. Source data are available online for this figure.

**Figure 3:**
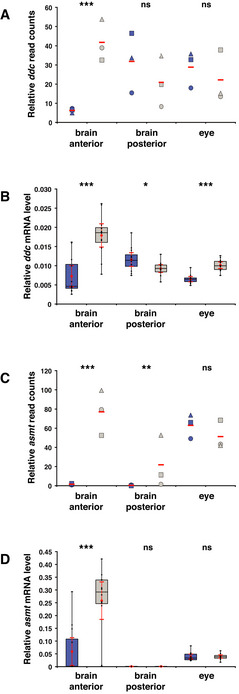
Melatonin pathway genes are overexpressed in the *opn4* dko anterior brain Transcriptome sequencing reveals a significant higher number of *ddc* reads in the *opn4* dko than in the wild‐type anterior brain, and no difference in the posterior brain and eyes. *ddc* reads were normalised to 1,000 *actb1* reads.qPCR confirms a significant higher *ddc* transcript level in the *opn4* dko (grey box) than in the wild‐type (blue box) anterior brain. In contrast to the transcriptome data, a significant higher level of *ddc* was detected by qPCR in the *opn4* dko eyes. Quantification of the *ddc* mRNA level is relative to the *actb1* mRNA measured in the sample.Chart shows normalised read counts for *asmt*. A significant higher number of *asmt* reads is detected in the *opn4* dko than in the wild‐type anterior brain. Note that *asmt* mRNA is detected in the posterior brain because the pineal stalk is most likely included in this part.qPCR confirms a significant higher *asmt* transcript level in the *opn4* dko than in the wild‐type anterior brain. Quantification of the *asmt* mRNA level is relative to the *actb1* mRNA measured in the sample. Data information: In (A) and (C), blue markers indicate wild‐type, grey markers indicate *opn4* dko and red bar indicates mean. Biological replicates are indicated with round, triangular and square markers (*n* = 3). Asterisks indicate significance (0.01 < *P*(*) < 0.05, 0.001 < *P*(**) < 0.01, *P*(***) < 0.001, ns = not significant). In (B) and (D), boxplot divides the data in quartiles: the box indicates the interquartile range, with the horizontal line in the box denoting the median of the data set, the whiskers extend to the minimum and maximum, and meet the box at the median of the lower (quartile 1) and median of the upper (quartile 3) half of the dataset. Black dots indicate biological replicates (*n* = 12), the red dot indicates the mean and red error bars indicate the confidence interval (95%).

Transcriptome sequencing was applied on cDNA from eyes and brain parts of wild‐type and *opn4* dko to reveal genes that act downstream of Opn4. For this purpose mature fish were used, as it is not possible to dissect larval brains due to their small size. The fish were entrained for 14 days to a 12:12 h LD regime, followed by dissection of the eyes and brains in the light phase at ZT4‐6 (Zeitgeber Time). The brains were separated in an anterior and posterior part (Fig 2A) to reduce complexity. The anterior brain consists of a part of the forebrain including the pineal. The posterior brain consists of the mid‐ and hindbrain and the remaining part of the forebrain, which comprises the whole hypothalamus and pretectum, and part of the epithalamus (i.e. habenula), thalamus and posterior tuberculum. The olfactory bulbs and pituitary were omitted. Following transcriptome sequencing the read counts were analysed with edgeR software in which the cutoff for differential expression was set at a significance level of α = 0.05 and the false discovery rate (FDR) was applied (Benjamini & Hochberg, 1995). Most differentially expressed genes are in the brain: 284 in the anterior brain, 139 in the posterior brain and 16 in the eye (Fig 2B, Appendix Fig S1A–C). Next, the differentially expressed genes were analysed with KEGG software to identify clusters of gene products that act in the same pathway. This associated the differentially expressed genes from the *opn4* dko anterior brain with several pathways (Source Data). The largest clusters of genes were assigned to phototransduction (dre04744: 14 genes) and metabolic (dre01100: 15 genes) pathways (Fig 2D, Appendix Fig S1A). Of the latter, 5 genes were assigned to the subcategory tryptophan metabolism (dre00380): *tph1a* (*P* = 7.89 × 10^−7^), *tph2* (*P* = 1.89 × 10^−13^), *ddc* (*P* = 4.05 × 10^−11^), *aanat2* (*P* = 0.04) and *asmt* (*P* = 4.74 × 10^−38^). These encode all the enzymes required for melatonin synthesis (Fig 3A and C, Appendix Fig S1D–F). Many of the differentially expressed genes that were assigned to the phototransduction pathway have been reported to be expressed in the zebrafish pineal: *saga* (*P* = 2.46 × 10^−97^), *gngt1* (*P* = 1.56 × 10^−32^), *pde6gb* (*P* = 3.71 × 10^−11^) [Thisse *et al*, ZFIN direct data submission], *grk7a* (*P* = 6.32 × 10^−11^) (Rinner *et al*, 2005), *grk7b* (*P* = 0.047) (Rinner *et al*, 2005), *rcvrna* (*P* = 2.32 × 10^−11^) (Zang *et al*, 2015), *rcvrn2* (*P* = 1.21 × 10^−5^) (Zang *et al*, 2015) and *gnat1* (*P* = 8.52 × 10^−22^) (Lagman *et al*, 2015). In the posterior brain, two differentially expressed genes are linked to phototransduction: *gnat1* (*P* = 0.005) and *saga* (*P* = 0.004), and one to tryptophan metabolism: *asmt* (*P* = 0.001) (Appendix Fig S1B), which are also overexpressed in the anterior brain. Because the stalk of the pineal extends into the posterior brain, a small part of its specific transcriptome could be included in this data set. Fisher's exact test (Fisher, 1922) indicates that differential expression of the same genes in the anterior and posterior brain is unlikely due to coincidence (Fig 2C). The large number of common differentially expressed genes may well be the result of shared forebrain regions between these data sets. All data sets were also analysed for functional association with FuncAssociate software, applying a cutoff at the significance level α = 0.05. This revealed gene ontology (GO) attributes for differentially expressed genes of the eye and anterior brain (Source Data). The GO attributes of the latter are all related to the effect of light on biological processes (Fig 2E). Most of the genes that were associated with light‐dependent processes are expressed in the zebrafish pineal. These are *rbp4l* (*P* = 3.85 × 10^−60^), *arr3a* (*P* = 1.02 × 10^−26^), *rpe65a* (*P* = 9.44 × 10^−15^), *crx* (*P* = 2.69 × 10^−13^) (Gamse *et al*, 2002), *stra6* (*P* = 1.14 × 10^−5^), *rlbp1a* (*P* = 0.001), *aanat2*, *pde6gb* [Thisse *et al*, ZFIN direct data submission], *grk7a* (Rinner *et al*, 2005), *grk7b* (Rinner *et al*, 2005), *rcvrna* (Zang *et al*, 2015), *rcvrn2* (Zang *et al*, 2015), *rcvrn3* (*P* = 1.28 × 10^−45^) (Zang *et al*, 2015), *gnat1* (Lagman *et al*, 2015), *rbp3* (*P* = 6.87 × 10^−15^) (Nickerson *et al*, 2006), *irbpl* (*P* = 0.012) (Nickerson *et al*, 2006), *unc119.2* (*P* = 0.0003) (Toyama *et al*, 2009) and *pp2* (*P* = 1.23 × 10^−6^) (Koyanagi *et al*, 2015). Also *otx5* (*P* = 0.001) and *lhx4* (*P* = 0.003) (Appendix Fig S1A,G,I) have been demonstrated to be expressed in the pineal (Gamse *et al*, 2002; Weger *et al*, 2016). Note that all the genes that are associated with the pineal are overexpressed in the *opn4* dko anterior brain (Appendix Fig S1A). As the pineal produces melatonin and detects light (Klein, 2004; Sapède & Cau, 2013), the differentially expressed genes assigned to the melatonin and phototransduction pathways in the *opn4* dko anterior brain (Fig 2D and E, Appendix Fig S1A) point to a defect in this gland. In addition to these pathways, five differentially expressed genes in the *opn4* dko anterior brain were assigned to the mitogen‐activated protein kinase (MAPK) pathway: *ngfra* (*P* = 0.042), *dusp7* (*P* = 0.002), *ppm1na* (*P* = 1.92 × 10^−6^), *daxx* (*P* = 0.006), *jund* (*P* = 0.048) and several genes were assigned to a range of pathways with diverse biological functions (Source Data). Two differentially expressed genes in the eye: *mhc1uba* (*P* = 7.06 × 10^−96^) and *mhc1uka* (*P* = 3.05 × 10^−7^) (Appendix Fig S1C,P and Q) were both associated with the GO attributes of antigen processing and presentation of peptide antigen (GO:0048002) and antigen binding (GO:0003823). Importantly, a substantial number of differentially expressed genes in the *opn4* dko function in the immune response: *mhc1uba* (*P*_a_ = 3.46 × 10^−185^, *P*_p_ = 7.89 × 10^−210^), *mhc1uka* (*P*_a_ = 0.04), *caspb* (*P*_a_ = 0.0005, *P*_p_ = 4.89 × 10^−9^), *caspbl* (*P*_a_ = 0.046), *ly6pge* (*P*_a_ = 3.10 × 10^−9^), *nrp1b* (*P*_a_ = 0.027), *mpeg1.1* (*P*_a_ = 0.049), *mrc1* (*P*_a_ = 0.012), *pigrl3.5* (*P*_a_ = 0.042), *dicp3.3* (*P*_p_ = 0.0008), *tap2a* (*P*_p_ = 0.037), *timd4* (*P*_p_ = 0.002), *cd74b* (*P*_p_ = 0.015), *b2m* (*P*_p_ = 0.047) and cell division: *mcm7* (*P*_a_ = 2.15 × 10^−13^, *P*_p_ = 2.30 × 10^−31^) *cdk9* (*P*_a_ = 3.45 × 10^−11^, *P*_p_ = 2.47 × 10^−5^), *ccna2* (*P*_p_ = 0.040), *cdk20* (*P*_p_ = 0.038) (Appendix Fig S1A–C, K–Q), implying that Opn4 controls diverse processes and confirming previous reports of cell cycle regulation by light (Dekens *et al*, 2003; Kowalska *et al*, 2013), regulation of the immune/inflammation response by the rat pineal (Bailey *et al*, 2009) and the role of melatonin in buffering the immune system (Carrillo‐Vico *et al*, 2013).

### *asmt* is overexpressed in the *opn4* dko pineal

**Figure EV2:**
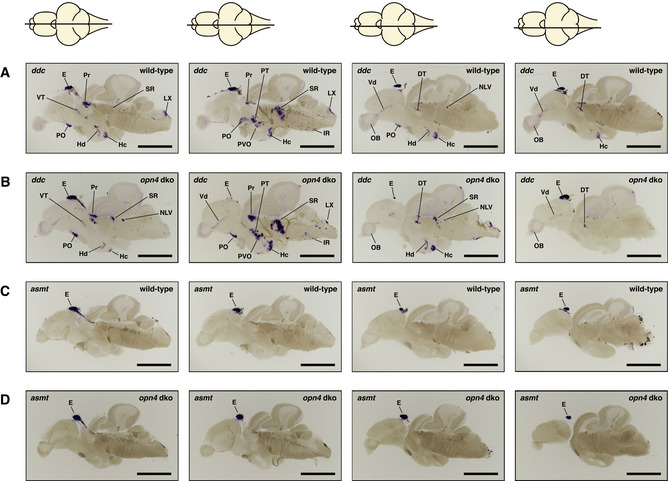
*ish* with *ddc* or *asmt* probe on *opn4* dko mature brains show wild‐type expression patterns A, BSame *ddc* expression pattern was observed in wild‐type (A) and *opn4* dko (B) brains (pineal lost from the brain slices in (B) centre panels).C, D
*asmt* expression in wild‐type (C) and *opn4* dko (D) adult brains show that *asmt* is not ectopically expressed. Thus, the higher *asmt* transcript level measured with qPCR in the *opn4* dko anterior brain can be attributed to the pineal. Data information: The horizontal line through a representation of the brain, above the section, indicates the location of the section within the brain. Abbreviations: epiphysis cerebri (pineal) [E], caudal zone of periventricular hypothalamus [Hc], inferior raphe [IR], periventricular pretectum [Pr], preoptic area [PO], posterior tuberculum [PT], paraventricular organ [PVO], suprachiasmatic nucleus [SCN], superior raphe [SR]. Note that the duration of the colorimetric development of the *ishs* in this figure is not exactly the same. Scale bar: 1.0 mm.

To validate the transcriptome sequencing data, we repeated the same entrainment and sampling procedure and determined the levels of *ddc* and *asmt* mRNA by qPCR, which confirmed significant overexpression of *ddc* (Fig 3B *U*‐test: *P* = 4.42 × 10^−5^) and *asmt* (Fig 3D *U*‐test: *P* = 0.0004) in the mature *opn4* dko anterior brain. Both *ddc* and *asmt* transcripts are expressed in the mature wild‐type and *opn4* dko pineals, as demonstrated by *ish* (Fig EV2A–D). The elevated *asmt* mRNA levels detected in the *opn4* dko brain (Fig 3C and D) can be attributed to the pineal, as *asmt* is solely expressed in this gland and not ectopically expressed in the *opn4* dko brain. Note that *asmt* is not overexpressed in the eye (Fig 3C and D).

### *opn4* dko larvae show attenuated locomotor activity in the wake state

**Figure 4:**
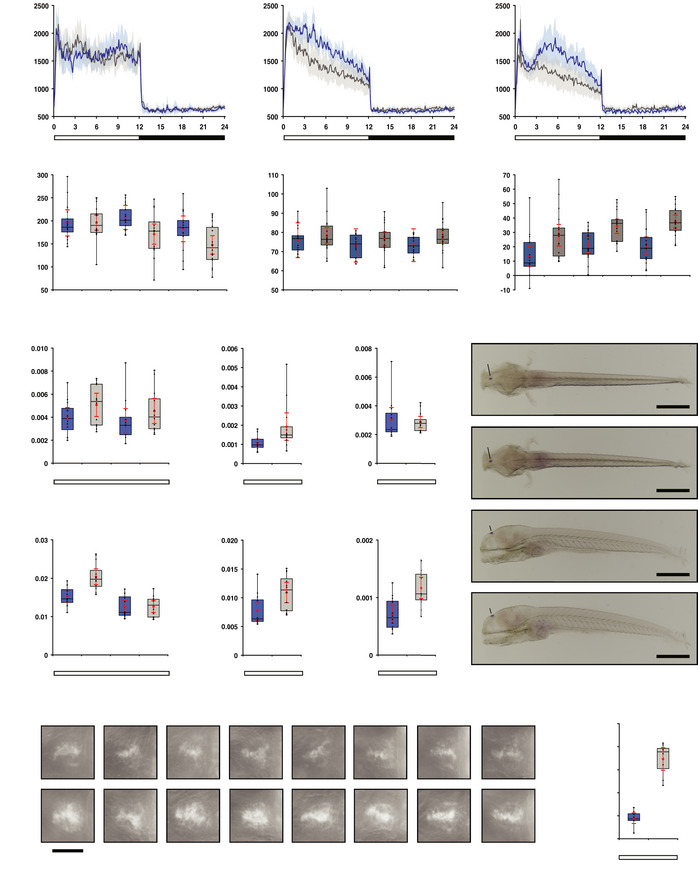
*opn4* dko larvae display attenuated locomotor activity in the wake state A–CActograms show mean locomotor activity of wild‐type (blue line) and *opn4* dko (grey line) larvae at (A) 5 dpf, (B) 6 dpf and (C) 7 dpf. The band on each side of the mean indicates the confidence interval (95%). The bar under the chart indicates the 12:12 h LD regime.DBox plot shows reduced locomotor activity during the wake state in *opn4* dko larvae (grey box) when compared to wild‐type (blue box) at 6 and 7 dpf. All activity bins in the light phase of each larva were pooled separately, thus the aggregated activity data of each larva on one particular day is in this case one single observation in the test statistics. As the consecutive days are presented separately, the assumption of independent identical observations is fulfiled.EPlot as in (D) for the rest state, shows no difference at 5 and 6 dpf and a significantly raised activity of the *opn4* dko at 7 dpf.F
*opn4* dko larvae show significant more displacement in large‐angle turns at the onset of the dark interval than wild‐type on the 5^th^, 6^th^ and 7^th^ dpf (see also Fig EV3A–C).GPlot shows *asmt* mRNA levels, detected by qPCR, in whole wild‐type (blue box) and whole *opn4* dko (grey box) larvae at 6 dpf.HPlot shows a significant higher *asmt* mRNA level in the ophthalmectomised *opn4* dko larvae than ophthalmectomised wild‐type larvae at ZT3 on the 6^th^ dpf.IPlot shows similar *asmt* mRNA levels between *opn4* dko eyes and wild‐type eyes at ZT3 on the 6^th^ dpf.J
*ish* with *asmt* probe on larvae sampled on the 6^th^ dpf at ZT3 shows that *asmt* mRNA is confined to the pineal and indicates that the *asmt* gene is not ectopically expressed in *opn4* dko larvae. The higher *asmt* mRNA level measured by qPCR in the *opn4* dko therefore points at a defect in the pineal. Note that the eyes were removed after the *ish* for optimal visibility of expression in the brain. Scale bar: 0.5 mm.KPlot as in (G), shows significant higher levels of *ddc* mRNA in whole *opn4* dko larvae at ZT3.LPlot as in (H), shows significant higher levels of *ddc* mRNA in ophthalmectomised *opn4* dko at ZT3.MPlot as in (I), shows significant higher levels of *ddc* mRNA in *opn4* dko eyes at ZT3.N
*ish* with *ddc* probe on wild‐type and *opn4* dko larvae sampled on the 6^th^ dpf represents *ddc* expression in pineals. Whole larvae are presented in Appendix Fig S2. Biological replicates indicated with BR. Scale bar: 0.05 mm.OQuantification of the adjusted intensity in the pineals from larvae presented in (N), implies a significant higher *ddc* transcript level in the *opn4* dko pineal. Data information: In (D–I), (K–M) and (O), boxplot divides the data in quartiles: the box indicates the interquartile range, with the horizontal line in the box denoting the median of the data set, the whiskers extend to the minimum and maximum, and meet the box at the median of the lower (quartile 1) and median of the upper (quartile 3) half of the dataset. Black dots indicate biological replicates (D–F: *n* = 18, G–I and K–M: *n* = 12, O: *n* = 8), the red dot indicates the mean, red error bars indicate the confidence interval (95%), asterisks indicate significance (0.01 < *P*(*) < 0.05, 0.001 < *P*(**) < 0.01, *P*(***) < 0.001, ns = not significant).

**Figure 5:**
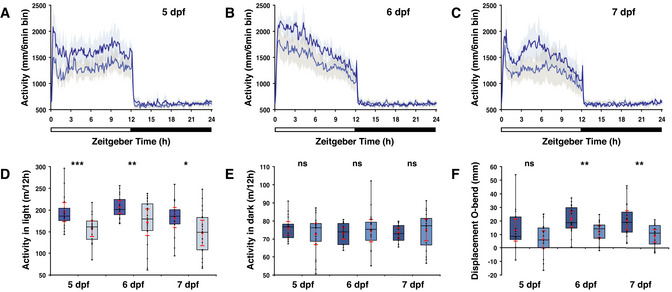
Larvae adapt their locomotor activity to the ambient light level A–CActograms show mean locomotor activity in wild‐type under a medium (dark blue line) and low (light blue line) intensity LD regime at (A) 5 dpf, (B) 6 dpf and (C) 7 dpf. Spectrum and photoperiod is the same under both conditions. The band on each side of the mean indicates the 95% confidence interval. The bar under the chart indicates the 12:12 h LD interval.DBox plot shows a significant reduction in locomotor activity during the wake state under a low (light blue box) than a medium (dark blue box) intensity LD regime on the 5^th^, 6^th^ and 7^th^ dpf.EAs in (D) for the rest state (dark phase). No difference in locomotor activity was detected.FLarvae show significant less displacement in large‐angle turns at the onset of the dark interval under a low intensity LD regime than under a medium intensity LD regime on the 6^th^ and 7^th^ dpf (see also Fig EV3D–F and I). Data information: In (D–F), boxplot divides the data in quartiles: the box indicates the interquartile range, with the horizontal line in the box denoting the median of the data set, the whiskers extend to the minimum and maximum, and meet the box at the median of the lower (quartile 1) and median of the upper (quartile 3) half of the dataset. Black dots indicate biological replicates (*n* = 18), the red dot indicates the mean, red error bars indicate the confidence interval (95%) and asterisks indicate significance (0.01 < *P*(*) < 0.05, 0.001 < *P*(**) < 0.01, *P*(***) < 0.001, ns = not significant).

**Figure EV3:**
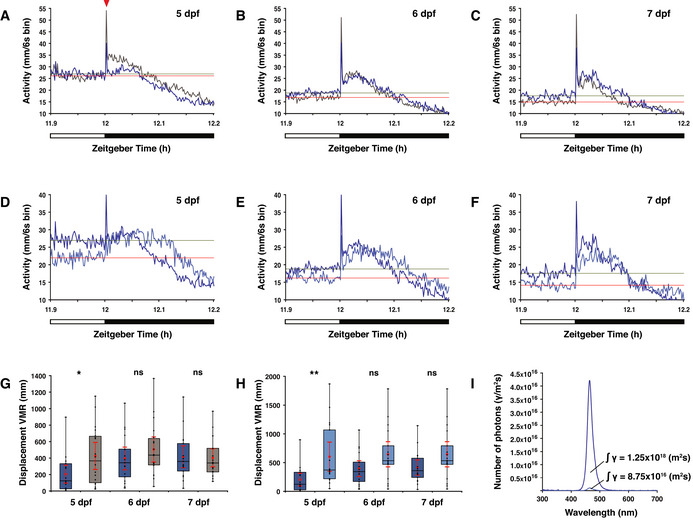
Overview of visual motor response (VMR) data A–CActograms show the mean activity of wild‐type (dark blue line) and *opn4* dko (grey line) under a medium intensity LD 12:12 h regime at the light to dark boundary on the (A) 5^th^ dpf, (B) 6^th^ dpf and (C) 7^th^ dpf. O‐bend spike indicated with red arrowhead. The bar under the chart indicates the last 6 min of the light phase and the first 12 min of the dark phase. Solely for estimation, the mean activity over the last 6 min in the light phase is plotted as a horizontal line for wild‐type (green) and *opn4* dko (red).D–FActogram shows mean activity of wild‐type larvae under a medium (dark blue line) or low (light blue line) intensity LD 12:12 h regime at the light to dark boundary on the (D) 5^th^ dpf, (E) 6^th^ dpf and (F) 7^th^ dpf. Solely for estimation, the mean activity over the last 6 min in the light phase is plotted as a horizontal line for larvae under medium light intensity (green) and low light intensity (red).G
*opn4* dko larvae (grey box) show similar displacement in routine turns after transition from light to darkness as wild‐type (blue box) on the 6^th^ and 7^th^ dpf, but not on the 5^th^ dpf (*U*‐test, *P* = 0.0413). Note that displacement of each individual larva was calculated separately by comparison with its own baseline activity in the light phase. Thus the displacement of each larva on one particular day is in this case one single observation in the test statistics.HWild‐type larvae show similar displacement in routine turns after transition from light to darkness under a low (light blue box) as under a medium (dark blue box) intensity LD regime on the 6^th^ and 7^th^ dpf, but not on the 5^th^ dpf (*U*‐test, *P* = 0.0029).IPlot of the photon flux at medium (dark blue line) and low (light blue line) light intensities. For biological processes, the photon flux has a higher relevance than the irradiance (power of electromagnetic radiation) because a photoreceptor is activated by a photon. The photon flux is defined as the number of photons (γ) per unit area (m^2^/s). Data information: In (G) and (H), boxplot divides the data in quartiles: the box indicates the interquartile range, with the horizontal line in the box denoting the median of the data set, the whiskers extend to the minimum and maximum, and meet the box at the median of the lower (quartile 1) and median of the upper (quartile 3) half of the dataset. Black dots indicate biological replicates (*n* = 18), the red dot indicates the mean, red error bars indicate the 95% confidence interval and asterisks indicate significance (0.001 < *P*(**) < 0.01, *P*(***) < 0.001, ns = not significant).

As larvae are routinely subjected to behaviour and neuropharmacology studies (Basnet *et al*, 2019) and the commercially available behaviour systems have been designed for larvae, we investigated locomotor activity at the larval stage. The characteristics of mature swimming emerge on the 4^th^ day post fertilisation (dpf) when the swim bladder becomes functional. Thereafter, larvae synchronise locomotor activity to the light‐dark phases (Hurd & Cahill, 2002). Locomotor activity or velocity is defined as the rate of change of a larva's position in a unit time. Wild‐type and *opn4* dko larvae were entrained to a 12:12 h LD regime in the DanioVision automated observation chamber from the 1^st^ dpf (~30 hpf) onwards, and recorded and tracked on the 5^th^, 6^th^ and 7^th^ dpf. *opn4* dko larvae display significantly lower locomotor activity than wild‐type during the light phase (wake state) at 6 dpf (Fig 4B and D, *P* = 0.009, *d* = −0.938) and 7 dpf (Fig 4C and D, *P* = 0.017, *d* = −0.851), but not at 5 dpf (Fig 4A and D), and activity is slightly raised during the night at 7 dpf (Fig 4E, *P* = 0.047, *d* = 0.699). *opn4* dko activity is reduced by 27% at 6 dpf when comparing the mean activity between wild‐type and the *opn4* dko in the light phase and setting the baseline to the mean activity of wild‐type in the dark phase. To determine if the difference between the wild‐type and *opn4* dko locomotor activity is due to interindividual variability or is the result of the mutant phenotype, we calculated Cohen's effect size (*d*), which evaluates the difference between the means by comparison with the pooled standard deviation. The effect size at 6 dpf (*d* = −0.938) shows a difference of 94% of the pooled standard deviation between the means of the wild‐type and *opn4* dko locomotor activity in the light phase. At 7 dpf the effect size (*d* = −0.851) shows a difference between the means in the light phase of 85% of the pooled standard deviation. Cohen defined an effect size of |*d*| > 0.8 as large (Cohen, 1988). Note that when we set the baseline at 0 we get similar statistical outcomes (6 dpf: *P* = 0.009, *d* = −0.932, 7 dpf: *P* = 0.020, *d* = −0.828). The significance and effect size independently indicate a very high probability that the observed differences in locomotor activity between wild‐type and *opn4* dko are the result of the phenotype. We also compared the visual motor response (VMR), which is defined as the brief increase in activity after loss of illumination. The VMR consists of two components: large‐angle turns (O‐bend spike) followed by routine turns (R‐turns) (Burgess & Granato, 2007; Fernandes *et al*, 2012). The *opn4* dko showed significant more movement in the large‐angle turns compared to wild‐type (Fig 4, 5, 5 dpf: *P* = 0.008, *d* = 0.955, 6 dpf: *P* = 0.001, *d* = 1.216, 7 dpf: *P* = 8.93 × 10^−5^, *d* = 1.509), while the routine turns were not affected on the 6^th^ and 7^th^ dpf (Fig EV3A–C and G).

**Figure EV4:**
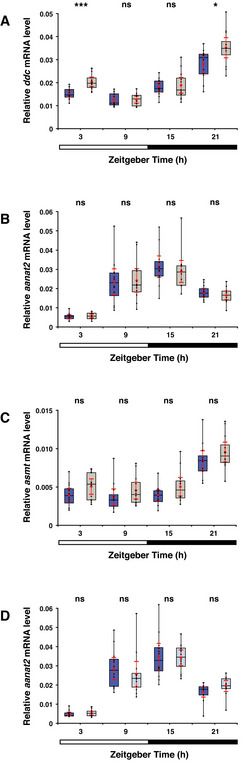
Expression of genes encoding melatonin synthesis enzymes on the 6^th^ dpf Box plot shows significant higher *ddc* mRNA levels measured by qPCR in whole *opn4* dko larvae (grey box) at ZT3 and ZT21 (*P* = 0.0240) than in wild‐type (blue box). Bar under the chart indicates the 12:12 h LD interval.As in (A) for the *aanat2* mRNA level, shows no significant difference between wild‐type and *opn4* dko whole lavae.As in (A) for the *asmt* mRNA level, shows no significant difference between wild‐type and *opn4* dko whole lavae.Box plot shows *aanat2* mRNA. The difference in mRNA levels between wild‐type larvae under a medium (dark blue box) or low (light blue box) intensity LD regime is not significant (ZT21: *U*‐test, *P* = 0.0684). Data information: boxplot divides the data in quartiles: the box indicates the interquartile range, with the horizontal line in the box denoting the median of the data set, the whiskers extend to the minimum and maximum, and meet the box at the median of the lower (quartile 1) and median of the upper (quartile 3) half of the dataset. Black dots indicate biological replicates (*n* = 12), the red dot indicates the mean, red error bars indicate the confidence interval (95%), asterisks indicate significance (0.001 < *P*(**) < 0.01, *P*(***) < 0.001, ns = not significant).

We further investigated *opn4* dko larvae on the molecular level. Larvae were entrained to a 12:12 h LD regime and *asmt* transcript levels were measured by qPCR in whole wild‐type and *opn4* dko larvae sampled on the 6^th^ dpf. The *asmt* mRNA levels did not show a significant difference (Figs 4G and EV4C). However, the expression of *asmt* in the eye and pineal returns the sum of the dissimilar relative expression levels. If a much higher *asmt* transcript level is present in the eye than in the pineal, an unaffected *asmt* transcript level in *opn4* dko eyes could potentially mask an increase in the *asmt* transcript level in the *opn4* dko pineal when comparing RNA extracts from whole wild‐type with *opn4* dko larvae. Therefore, wild‐type and *opn4* dko larvae were entrained to the LD regime, sampled and ophthalmectomised at 6 dpf between ZT3 and ZT4, and the *asmt* transcript levels were determined by qPCR in ophthalmectomised larvae and their eyes separately. This showed significant overexpression of *asmt* in ophthalmectomised *opn4* dko larvae compared to wild‐type larvae (Fig 4H, *U*‐test: *P* = 0.017) and similar *asmt* expression levels in the eyes (Fig 4I). Whole mount *ish* for *asmt* in *opn4* dko 6 dpf larvae revealed that *asmt* is not ectopically expressed (Fig 4J), demonstrating that the increase in *asmt* transcript level in the ophthalmectomised *opn4* dko larvae can be solely attributed to the pineal. qPCR to detect *ddc* mRNA in the same samples showed that this transcript is significantly overexpressed in whole *opn4* dko larvae (Fig 4K, ZT3: *P* = 0.0005, Fig EV4A), ophthalmectomised *opn4* dko larvae (Fig 4L, *U*‐test: *P* = 0.0045) and their eyes (Fig 4M, *P* = 0.0005). However, the higher *ddc* mRNA level in ophthalmectomised *opn4* dko larvae does not give insight into the expression level in the pineal, as *ddc* is expressed in multiple brain areas. Thus, we performed an *ish* for *ddc*, and assayed the *ddc* transcript levels in the pineals of individual 6 dpf larvae by measuring the adjusted volume intensity. This experiment revealed significant higher *ddc* transcript levels in the *opn4* dko than in the wild‐type pineal (Fig 4N and O, *P* = 2.12 × 10^−7^, Appendix Fig S2). *tyrosine hydroxylases* (*th1, th2*), which encode essential enzymes for dopamine synthesis, and the dopamine transporter (*slc6a3*) are not expressed in the pineal (Filippi *et al*, 2010), implying that dopamine is not produced by this gland. Thus, the overexpression of *ddc* in the pineal predicts a higher serotonin level. As *aanat2* is only expressed in the pineal the transcript was measured in whole larvae, and similar *aanat2* mRNA levels were detected in wild‐type and *opn4* dko (Fig EV4B, ZT21: *P* = 0.318). The overexpression of multiple genes that encode melatonin synthesis enzymes in the pineal of the *opn4* dko larva suggests a higher melatonin level, which would provide a causal explanation for its attenuated locomotor activity during the wake state.

### Larvae adapt their locomotor activity to the light level

**Figure 6:**
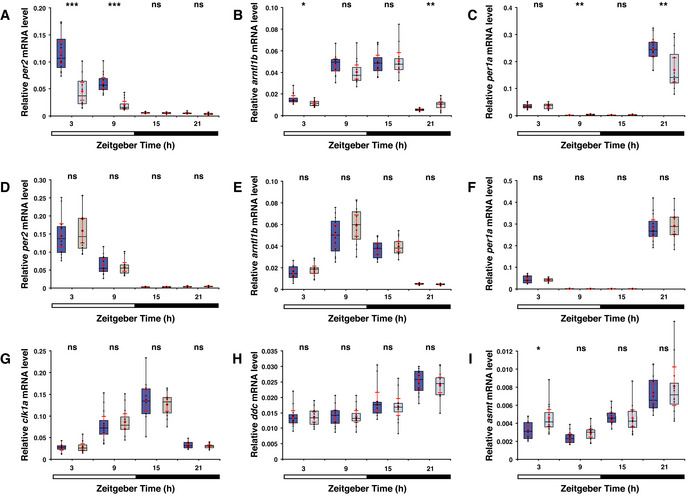
The light level is correlated with *per2* and *asmt* transcript levels Per2 is known to act as an intermediary between the light detector and the melatonin synthesis pathway. Box plot shows *per2* mRNA levels measured by qPCR in 6 dpf larvae placed under a low (light blue box) or medium intensity (dark blue box) LD regime. Under the low intensity light phase a significant reduction in the *per2* transcript levels is detected. Bar under the chart indicates the 12:12 h LD interval.Box plot as in (A) shows mRNA levels of *arntl1b*, which is in part regulated by *per2*. Under low intensity light a significant reduction in the *arntl1b* transcript level is detected at ZT3 and an increase at ZT21.Plot as in (A) shows mRNA levels of *per1a*, which is regulated by the Arntl‐Clk heterodimer. Under low intensity light a significant reduction in the *per1a* transcript level is detected at ZT21 and an increase at ZT9.Box plot shows similar *per2* mRNA levels between wild‐type (blue box) and *opn4* dko (grey box) under a medium intensity LD regime at 6^th^ dpf.Box plot as in (D), shows similar *arntl1b* mRNA levels.Box plot as in (D), shows similar *per1a* mRNA levels.Box plot as in (D), shows similar *clk1a* mRNA levels.Box plot as in (A) shows *ddc* mRNA levels. Similar levels were detected between wild‐type larvae under a low or medium intensity LD regime.Box plot as in (A) shows *asmt* mRNA levels. Under a low intensity LD regime a significant increase in the *asmt* transcript level is detected at ZT3. Data information: boxplot divides the data in quartiles: the box indicates the interquartile range, with the horizontal line in the box denoting the median of the data set, the whiskers extend to the minimum and maximum, and meet the box at the median of the lower (quartile 1) and median of the upper (quartile 3) half of the dataset. Black dots indicate biological replicates (*n* = 12), the red dot indicates the mean, red error bars indicate the 95% confidence interval and asterisks indicate significance (0.01 < *P*(*) < 0.05, 0.001 < *P*(**) < 0.01, *P*(***) < 0.001, ns = not significant).

Light has been demonstrated to suppress the rest state in diurnal vertebrates (Lowrey & Takahashi, 2000; Yokogawa *et al*, 2007). Thus, the intensity of light may also affect locomotor activity during the wake state. To test this, wild‐type larvae exposed to medium light intensity were compared with wild‐type exposed to more than an order of magnitude reduced light intensity (Fig EV3I), both under the same photoperiod and spectrum. Larvae under a low intensity LD regime were significantly less active in the light phase than under a medium intensity LD regime at 5 dpf (*P* = 0.001, *d* = −1.213), 6 dpf (*P* = 0.015, *d* = −0.933) and 7 dpf (*P* = 0.011, *d* = −0.887) (Fig 5A–D), and showed no difference in activity during the dark phase (Fig 5E). The significance and effect size independently indicate a very low probability that the observed differences in locomotor activity are the result of interindividual variability. To determine whether the light intensity also affects the expression of genes that encode melatonin synthesis enzymes, we compared *ddc*, *aanat2* and *asmt* transcript levels at 6 dpf under the same conditions. A significant increase in the *asmt* transcript level was detected in the light phase of the low intensity LD regime (Fig 6I, *U*‐test at ZT3: *P* = 0.0284), but *ddc* (Fig 6H, ZT21: *P* = 0.538, ZT3: *P* = 0.953) and *aanat2* (Fig EV4D, *U*‐test at ZT21: *P* = 0.068, ZT3: *P* = 0.553) mRNA levels did not show significant differences. Thus, solely reducing the light intensity without changing the photoperiod and spectrum induces *asmt* transcription and reduces locomotor activity during the wake state. In addition, a reduction in large‐angle turns was observed under the low intensity LD regime (Fig 5F 6 dpf: *P* = 0.005, *d* = −1.024, 7 dpf: *P* = 0.004, *d* = −1.083), which further demonstrates that zebrafish can detect differences in the light level.

### Light intensity and core clock gene transcription are correlated

Per2 functions as an intermediary between the light signal and the melatonin synthesis pathway, as light‐dependent onset of *aanat2* transcription in the pineal requires Per2 (Ziv *et al*, 2005; Ziv & Gothilf, 2006) and as *per2* transcription has been shown to be directly light induced (Cermakian *et al*, 2002; Ziv *et al*, 2005; Dekens & Whitmore, 2008). Therefore, we measured the transcript levels of *per2* in 6 dpf larvae at ZT3, 9, 15 and 21 under the stated conditions, which revealed a significant reduction in the *per2* transcript level under the low intensity light phase (Fig 6A, ZT3: *P* = 2.16 × 10^−5^, *U*‐test at ZT9: *P* = 1.16 × 10^−6^). Per2 has been reported to regulate the core clock gene *arntl1b* (Wang *et al*, 2015). Consistent with this report, we detected a significant reduction in the *arntl1b* mRNA level (Fig 6B, *U*‐test at ZT3: *P* = 0.0205, ZT21: *P* = 0.0065) under the low intensity LD regime. Next, we measured the expression of *per1a*, which is under the control of the Clk‐Arntl heterodimer, and detected a significant difference in *per1a* mRNA levels between the low and medium intensity LD regimes (Fig 6C, ZT9: *P* = 0.0015, ZT21: *P* = 0.0095). As mouse OPN4 also functions in clock resetting, we compared the transcript levels of *per2* (Fig 6D), *arntl1b* (Fig 6E), *per1a* (Fig 6F) and *clk1a* (Fig 6G) on the 6^th^ dpf at ZT3, 9, 15 and 21 between wild‐type and *opn4* dko larvae. We detected no significant differences in transcript levels, suggesting that during this larval stage *opn4.1* and *opn4xb* do not play a role in clock resetting or are redundant in this process. Transcriptome sequencing on cDNA of adult posterior brains that were sampled in the light phase showed a significant reduction in the transcript level of *per1a* (*P* = 0.0153), *roraa* (*P* = 0.0365) and *cdk5* (*P* = 0.0093), and a similar trend in *per2* (Appendix Fig S1B,S–V) in the *opn4* dko, implying that Opn4.1‐Opn4xb input into the clock when the fish has matured. Furthermore, several MAPK pathway genes are differentially expressed, which may point to an altered clock amplitude and/or rhythm, as this pathway has been reported to input into the circadian clock (Cermakian *et al*, 2002).

## Discussion

### A comparison between mammals and zebrafish

The traditionally studied vertebrate genetic model systems in chronobiology are nocturnal rodents. The zebrafish offers insight in the diurnal chronotype, a perspective from a different class of vertebrates and suitability for genetic manipulation. Whereas the function of OPN4 in the rodent ipRGCs has been extensively studied, the function of Opn4 in the zebrafish pineal is unknown. In addition, the zebrafish is relevant for studies on the regulation of the melatonin pathway (Gandhi *et al*, 2015), while genes encoding melatonin synthesis enzymes are nonfunctional in most laboratory mouse strains (Ebihara *et al*, 1987; Roseboom *et al*, 1998).

A common function for Opn4/OPN4 in zebrafish and rodents is not evident, given the drastically different connection of the pineal with its light input and as the zebrafish expresses several distinct non‐visual photoreceptors. For instance, Exo‐rhodopsin (Exorh) has been proposed as the primary candidate for regulating the melatonin pathway in the zebrafish pineal, as it has been shown to induce *aanat2* during embryonic development (Pierce *et al*, 2008; Ben‐Moshe *et al*, 2014). Furthermore, the transcriptome data presented here shows overexpression of *parapinopsin* (*pp2*) in the *opn4* dko pineal, which could be due to a compensatory mechanism and therefore Pp2 may be involved in the same process (Koyanagi *et al*, 2015). The suppression of melatonin synthesis by red light in zebrafish (Ziv *et al*, 2007) is another indication that different photoreceptors input in the melatonin pathway. However, these data do not exclude that one or more of the zebrafish Opn4 photoreceptors have a similar function as reported in mammals.

We demonstrate that adult zebrafish *opn4* dkos express in the light phase significantly higher levels of the transcripts: *tph1*, *tph2*, *ddc, aanat2* and *asmt* than wild‐type. These transcripts encode all the enzymes required for the conversion of tryptophan into melatonin. In addition, significantly higher transcript levels were detected of: *crx*, *otx2*, *otx5* and *lhx4* transcripts (Appendix Fig S1A,H,I,J), which encode homeodomain transcription factors that have been shown to regulate genes that encode melatonin pathway enzymes. CRX (Rohde *et al*, 2014b, 2019) and OTX2 (Rohde *et al*, 2019) regulate *Tph1*, *Aanat* and *Asmt* in the rat pineal, and LHX4 (Hertz *et al*, 2020) regulates *Aanat* in rats. Otx5 has been demonstrated to regulate *aanat2* in the zebrafish pineal (Gamse *et al*, 2002). These reports are consistent with the data presented here, and suggest a common mechanism for the regulation of melatonin synthesis through homeodomain transcription factors. Furthermore, the Opn4‐dependent transcriptome shows that *creb3l1* (Appendix Fig S1A,R, *P* = 0.028), which product may also be involved in the regulation of the melatonin pathway, is differentially regulated in the *opn4* dko. Its underexpression may predict a decrease in the transcription of CRE‐regulated genes. However, transcriptome sequencing does not reveal all regulatory processes, such as post‐translational modifications. *adenylate cyclase* (*adcy1* in Appendix Fig S1A, *P* = 0.012) is overexpressed in the *opn4* dko anterior brain, implying an increase in pCreb and the expression of the putative genes under its control, consistent with the observed higher *aanat2* transcript level and the presence of CRE elements in the *aanat2* promoter (Falcon *et al*, 2007). Interestingly, *aanat2* mRNA levels show no difference between wild‐type and *opn4* dko larvae, suggesting that at this stage Opn4.1‐Opn4xb are redundant or may not regulate *aanat2*. The reported role for Exorh in the regulation of *aanat2* raises the question if distinct photoreceptors could directy or via the clock control different steps of the melatonin pathway and/or if distinct photoreceptors could perform the same function at different stages of development. The appearance of the *opn4* dko locomotor activity phenotype on the 6^th^ dpf, after larvae can already adapt their locomotor activity to the light level, suggests that a developmental aspect plays a role. In addition to the discussed analogy on the molecular level between zebrafish and rodents, *opn4* dko larvae exhibit attenuated locomotor activity during the wake state in line with the mouse *Opn4* knockout wake state phenotype (Mrosovsky & Hattar, 2003; Lupi *et al*, 2008; Tsai *et al*, 2009). The zebrafish *opn4* dko phenotype also resembles the light‐dependent wakefulness observed in humans (Cajochen *et al*, 2000; Phipps‐Nelson *et al*, 2003; Lockley *et al*, 2006; Smolders *et al*, 2012; Smolders & de Kort, 2014; Knaier *et al*, 2016). Thus pointing to a similar function for Melanopsin in distant vertebrates.

### Locomotor activity in the diurnal and nocturnal wake states

We assessed the effect of light on locomotor activity in the diurnal zebrafish by double knockout of *melanopsins*, and demonstrate that Opn4.1‐Opn4xb elevate locomotor activity during the wake state. In nocturnal mice, OPN4 controls the light‐induced sleep state, as exposure to light during the night inhibits activity in wild‐type while *Opn4* mutants remain active (Mrosovsky & Hattar, 2003; Altimus *et al*, 2008; Lupi *et al*, 2008). This phenotype is the opposite of that observed in the zebrafish *opn4* dko, and is consistent with activity being induced by light in diurnal vertebrates (Shapiro & Hepburn, 1976; Tobler & Borbely, 1985; Yokogawa *et al*, 2007) and suppressed in nocturnal vertebrates (Campbell & Tobler, 1984; Mrosovsky & Hattar, 2003). Interestingly, mice with retinal degeneration (*rd/rd cl*) still show a reduction in melatonin levels after exposure to a light pulse in the night (Lucas *et al*, 1999). However, *rd/rd cl* mice that also lack OPN4 display a complete loss in responses to light (Hattar *et al*, 2003; Panda *et al*, 2003). Therefore, it is thought that mouse OPN4 plays a role in the light‐dependent suppression of melatonin.

The daily sleep‐wake rhythm is reversed between nocturnal and diurnal vertebrates (Shapiro & Hepburn, 1976; Campbell & Tobler, 1984; Yokogawa *et al*, 2007). Nevertheless, the higher level oscillators of diurnal and nocturnal vertebrates have a similar phase and function resulting in the melatonin level reaching its peak during the night (Challet, 2007). A common light signalling mechanism can be postulated as light represses, through Opn4, melatonin synthesis in both chronotypes. This repression brings about the opposite effect on the activity of diurnal and nocturnal vertebrates, suggesting that activity is inversely regulated downstream of the melatonin receptor. As the efficacy of melatonin receptors depends on receptor‐associated partners, cell‐dependent receptor expression and other context‐dependent factors (Cecon *et al*, 2018), system bias could be at the root of the opposite light‐dependent behaviour between diurnal and nocturnal animals.

### Input from the pineal versus the eye

The *opn4* dko displays a large‐angle turns phenotype, while a role for Opn4a has been reported in the routine turns of the VMR (Fernandes *et al*, 2012). The defect in large‐angle turns indicates a function for Opn4.1‐Opn4xb in the adaptation to loss of illumination. Large‐angle turns have been demonstrated to be regulated by the eye (Fernandes *et al*, 2012), thus pointing to a defect in the *opn4* dko eye. This can be explained by the expression of *opn4.1* in horizontal cells and single cone photoreceptors and *opn4xb* in bipolar cells (Davies *et al*, 2011), and overexpression of *ddc* in *opn4* dko eyes. The expression of *opn4.1* and *opn4xb* in the eyes raises the question if the eyes may also contribute to the *opn4* dko locomotor activity phenotype. However, the zebrafish pineal is not connected to the retinotectal projection (Robles *et al*, 2014) and so far sympathetic innervation could not be demonstrated. Importantly, zebrafish pineal function is not affected by catecholamine receptor agonists including NE (Cahill, 1997), thus excluding NE innervation as well as NE released in the circulation by chromaffin cells. Furthermore, the zebrafish pineal functions autonomously as shown by *in vitro* studies. Zebrafish pineals *in vitro* synthesise melatonin rhythmically in synchrony with the LD regime and continue rhythmic melatonin production under constant conditions (Cahill, 1996). The melatonin synthesised in the eyes has been shown in over 30 fish species to act as a paracrine signal and is not released in the blood (Zachmann *et al*, 1992a; Iigo *et al*, 1997; Iuvone *et al*, 2005; Falcon *et al*, 2007). Together, these data indicate that the reduced locomotor activity in the *opn4* dko wake state ought to be due to a defect in the pineal. It is plausible that the difference between rodents and zebrafish in indirect versus directly light regulation of the pineal is due to nocturnal versus diurnal niche adaptation.

### Locomotor activity, light intensity and Opn4 function

As light represses the rest state in diurnal vertebrates (Campbell & Tobler, 1984; Yokogawa *et al*, 2007), we investigated whether the intensity of light alone could affect locomotor activity during the wake state. We reasoned that reducing the light intensity may partially lift the inhibition on the expression of genes encoding melatonin synthesis enzymes and thereby raise the melatonin level (Max & Menaker, 1992; Zachmann *et al*, 1992b; Bolliet *et al*, 1995). A reduction in locomotor activity was measured during the wake state under a low intensity LD regime when compared to medium intensity with the same photoperiod and spectrum. This result is in accordance with a significant higher *asmt* transcript level detected in the light phase of larvae exposed to a low intensity LD regime. Thus, locomotor activity depends on light intensity and is correlated with the *asmt* transcript level. These data suggest that sustained repression of the melatonin pathway by light is required to elevate locomotor activity during the diurnal wake state. Importantly, light intensity defines the amplitude of *per2* transcription. The reduced *per2* transcript level and locomotor activity under a low intensity light phase is supported by the reduced locomotor activity of the *per2* mutant (Wang *et al*, 2015) and the role of *per2* in regulating *aanat2* transcription in the pineal (Ziv *et al*, 2005). While the circadian clock has been reported to control the melatonin pathway by regulating *aanat2* transcription (Klein, 2007), *aanat2* transcript levels between the medium and low intensity LD regimes did not reach a difference that is significant (Fig EV4D, ZT21: *U*‐test *P* = 0.068). However, qPCR does not reveal all regulatory processes, so that post‐transcriptional regulation cannot be excluded. Experimenting with light intensities does demonstrate the strong effect of short wavelength light on behaviour, as even dim light induces considerable locomotor activity in wild‐type. In contrast to the effect of light intensity on wild‐type, the *opn4* dko larvae do not show altered expression levels of the core clock genes: *per2*, *arntl1b* and *per1*, suggesting that at this stage Opn4.1‐Opn4xb may not function in resetting the circadian clock or are redundant. However, *opn4* dko larvae do not show a true block wave function as locomotion (Fig 4E) and the *ddc* mRNA level (Fig EV4A) are also affected in the dark phase. The data may suggest that Opn4.1‐Opn4xb exert more control over the clock in the adult. A comparison between the *opn4* dko and the light intensity experiments suggests that in zebrafish multiple distinct photoreceptors have the property to regulate locomotor activity. We did not knock out all *opn4* genes, which could potentially have a more profound effect on locomotor activity, as expression of the other *opn4* genes in the pineal cannot be excluded. The data collected in this study may well support future research to further our understanding of how light affects several non‐visual light‐dependent processes.

## Materials and Methods

### Animal husbandry

Animal procedures were conducted according to Austrian (BMWFW‐66.006/0012‐WF/II/3b/2014) and European (Directive 2010/63/EU) legislation covering the use of animals for scientific purposes. Zebrafish (*Danio rerio*) strains were cared for and bred as previously described (Mullins *et al*, 1994; Westerfield, 2007).

### *In situ* hybridisation

Labelled antisense RNA probes were synthesised following standard protocol. The probe was diluted in hybridisation buffer and heated for 2 min at 90°C. Mature fish were anaesthetised with MS‐222 (tricaine methane‐sulphonate, pH7) and then euthanised by decapitation. After removal of the eyes and lifting the skullcap, the head was fixed overnight (O/N) in formaldehyde (4% FA, pH7.5). Following careful brain dissection, the brains were washed in Phosphate Buffered Saline with 0.1% Tween‐20 (PBST, pH7.4) and permeabilised in 100% methanol (MeOH) O/N at −20°C. After removing MeOH through washes with successively lower percentage MeOH, the brains were permeabilised with proteinase K (10 μg/ml in PBST) for 30 min at room temperature (RT) followed by rinsing with glycine (10 mg/ml in PBST) to inactivate proteinase K, fixation and PBST washes. Brains were prehybridised for 2–4 h at 64°C, then hybridised with probe for ~40 h at 64°C, followed by multiple washes with successively lower formamide/SSC (Saline Sodium Citrate, pH6.0) concentrations and final washes with Maleic Acid Buffer (MABT, pH7.5) to remove unbound probe. Next, brains were embedded in 3% agarose in PBS and cut with the Leica VT1000S vibrating blade microtome in 100 μm sagittal sections. Note that sagittal sections show labelling of the pineal clearer than coronal sections. The sections were blocked for 2 h at RT (2% blocking reagent in MABT, Roche) and thereafter incubated with 1:2000 anti‐DIG‐AP (anti‐Digoxigenin coupled to Alkaline Phosphatase, Roche) in blocking buffer O/N at 4°C, followed by washes for several hours with MABT at RT and a final O/N wash at 4°C. For colorimetric development, the substrate BM‐Purple (Roche) was applied followed by fixation of the dye. Brain sections were mounted in Dako medium (Agilent). Larvae were fixed O/N in 4% FA followed by PBST washes and depigmentation with 100% acetone O/N at −20°C and treatment for 5 min at RT with 0.1 M KOH/ 3% H_2_O_2_. Larvae that had gone through the *in situ* procedure were mounted in 3% methylcellulose and dorsal and lateral images were captured with the Nikon SMZ18 microscope. To analyse the expression levels in larval pineals, the raw tif files of unsaturated and standardised exposure were converted to 8‐bit grey scale with Adobe Photoshop. Next, intensities were determined with ImageLab™ software from Bio‐Rad using volume tools and the same volume was applied to all replicates for calculating the adjusted volume intensity with the local background subtraction method.

### Site‐directed mutagenesis

Knockouts were generated with Transcription Activator‐Like Effectors fused to FokI Nuclease (TALEN), as previously described (Cermak *et al*, 2011; Bedell *et al*, 2012). The TALEs were designed with online TALEN targeter software (https://tale‐nt.cac.cornell.edu/) to bind exclusively to the target genes. The following settings were applied: spacer length of 15–20bp and guanine bound by the Repeat Variable Diresidue (RVD) NN, cytosine by HD, thymine by NG and adenine by NI. The different RVD sequences were subcloned in their order into the pFUS‐A and B plasmids with the GoldenGate method (TALEN assembly kit, Addgene). Next, TALE1 was subcloned into pCS2TAL3DD and TALE2 into the pCS2TAL3RR plasmid, each carrying the sequence of one of the two domains of the heterodimeric FokI nuclease (Miller *et al*, 2011). The intermediate and final constructs were transformed, screened by colony PCR, colonies grown O/N, plasmids isolated and sequenced for quality control. The final sequences were checked against the sequence of the full RVD arrays generated with the TAL plasmids sequence assembly tool (http://bao.rice.edu/). Thereafter the plasmids were linearised with KpnI, *in vitro* transcribed (SP6 mMessage mMachine kit, Invitrogen), mRNA purified (RNeasy kit, Qiagen) and a range of transcript concentrations was microinjected in zygotes. A target site in the *opn4* gene was selected with a unique restriction recognition sequence, which is lost when a mutation is introduced. For genotyping, the target site was amplified with PCR followed by a restriction digest on the amplicon (Table EV2). PCR was performed on a dilution (20x) of 10 μg/ml of proteinase K‐treated (2 h at 60°C) tail fin clips or 2 dpf offspring with primers designed to bind in introns (or the 5'‐UTR) to specifically amplify genomic DNA. When the restriction recognition site in the amplicon was absent, the type of mutation was identified by sequencing. TALENs were selected that generate premature STOP codons which are transmitted through the germline. The DNA‐binding domains of these TALENs are presented in Table 1 and their RVD sequences in Table EV3.

### Transcriptome sequencing

Photobiology protocols were followed as previously described (Dekens *et al*, 2017). Animals were exposed to blue light (peak wavelength LED: ~470 nm, Fig EV3I) covering the spectral sensitivity of Melanopsin. With monochromatic light we aim to reduce the number of activated photoreceptors. Even though zebrafish exhibits multiple blue light photoreceptors, we may to some extent minimise the effect of redundant photoreceptors on the analysis of the phenotype (Ziv *et al*, 2007). In addition, applying monochromatic light when comparing different light intensities has the advantage that the observed effect can be solely attributed to the reduction of the light level instead of a change in the spectrum (Dekens *et al*, 2017). Mature wild‐type and *opn4* dko fish of the same strain and age were entrained to a blue LD 12:12 h regime for 14 days in a synchronisation instrument at constant temperature (28 ± 0.2°C), and subsequently anaesthetised between ZT4 and ZT6. After decapitation, the head was pinned on silicone in a Petri dish filled with PBS under blue light (LED connected to fibre optic of dissection scope). The eyes were removed by cutting the optic nerves with surgical scissors (Fine Science Tools, FST) and the brain was carefully removed after lifting the skullcap with surgical forceps (FST) and cutting the spinal cord. The brain was separated in an anterior and posterior part with a surgical blade. For each biological replicate, the same tissue parts of three individuals were pooled and frozen in liquid nitrogen to obtain sufficient mRNA. A total of three biological replicates were processed. RNA isolation for sequencing was executed according to a modified version of an existing protocol (Mortazavi *et al*, 2008; Schenk *et al*, 2019). Total RNA was isolated by adding RNAzol (Sigma‐Aldrich) and a steal bead to the frozen sample, followed by disruption in a bead mill for 3 min at 30 Hz (TissueLyser II, Qiagen) and extraction of total RNA (Direct‐zol RNA kit, Zymo Research). mRNA was isolated from total RNA by selective binding to oligo‐dT coupled beads (Dynabeads mRNA purification kit, Invitrogen) followed by washes and elution. To increase purity the eluate was bound, washed and eluted for a second round, and thereafter quality checked (Bioanalyzer RNA 6000 Pico assay, Agilent). Next, mRNA was fragmented for 3 min at 75°C (fragmentation reagents, Invitrogen) and the fragment size distribution was quality checked (Bioanalyzer RNA 6000 Pico assay, Agilent). Subsequently, the mRNA was transcribed (SuperScript VILO cDNA synthesis Kit, Invitrogen) and libraries were constructed (ultra II kit, NEB), followed by adding barcodes with PCR (multiplex sequencing). The samples were single read 100bp high throughput sequenced with the Illumina HiSeq 2500 v4 chemistry. Reads were mapped to the reference genome (assembly GRCz10.86) with NextGenMap software and the number of reads per transcript was counted with the featureCounts tool. To assign genes to pathways, the Ensembl IDs of the differentially expressed genes were converted to KEGG IDs (https://biodbnet‐abcc.ncifcrf.gov/db/db2db.php) and input into KEGG software (https://www.genome.jp/kegg/tool/map_pathway2.html), and Ensembl gene IDs were input into FuncAssociate software (http://llama.mshri.on.ca/funcassociate) to reveal gene ontology attributes.

### Quantitative PCR

For the first ~30 h of development, embryos were raised in an incubator under a white LD regime. Thereafter the larvae were divided over two synchronisation instruments (Dekens *et al*, 2017), one set at a medium and the other at a low intensity blue LD 12:12 h regime (Fig EV3I) and constant temperature (28 ± 0.2°C), or wild‐type and *opn4* dko were placed in the same synchronisation instrument under a medium blue LD regime. The ILT950 spectrometer (International Light Technologies) was applied for light measurements. On the 6^th^ dpf at ZT3, 9, 15 and 21, 12 biological replicates were harvested at each time point for both conditions and placed in liquid nitrogen. Each replicate consists of 10 whole larvae. To determine transcript levels in eyes and ophthalmectomised larvae separately, the larvae were euthanised on the 6^th^ dpf between ZT3 and ZT4 and pinned with 0.1 mm insect pins on silicone in a Petri dish filled with PBS. One pin was placed in the trunk and another between eye and brain, followed by removal of the eyes with surgical forceps (FST) under blue light. The eyes and ophthalmectomised larvae were pooled separately, and 10 larvae were sampled for each biological replicate. In all experiments, a total of 12 biological replicates were harvested for each wild‐type and *opn4* dko. qPCRs for eyes and ophthalmectomised larvae were performed using the same amount of cDNA transcribed from the same amount of total RNA. The transcript levels in the eyes were normalised by the ratio of the yield in total RNA between eye and ophthalmectomised larval extracts. For whole larvae, ophthalmectomised larvae, mature brain parts and eyes, total RNA was isolated as described for transcriptome sequencing. Next, genomic DNA was eliminated for 2 min at 42°C and 1 μg total RNA was transcribed for 30 min at 42°C using random hexamers (QuantiTect reverse transcriptase kit, Qiagen) followed by inactivation for 3 min at 95°C. ProbeFinder software from Roche was used to design primers (Table EV4) that bind to neighbouring exons spanning the exon–exon junction to avoid amplification of genomic DNA, and generate a short amplicon to increase the overall efficiency of amplification. Note that the applied *aanat2* forward and reverse primers do not target *aanat1*. qPCR was performed with SYBR^®^ Green PCR master mix (Applied Biosystems) and 500 nM final concentration of each primer in the StepOnePlus™ thermocycler from AB. Amplification was executed with the thermocycling protocol: 10 min at 95°C followed by 40 cycles of 15 sec denaturation/annealing at 95°C and 1 min extension at 60°C. All wild‐type and mutant biological replicates that were harvested at the same ZT time were analysed together on the same 96 well plate. For each sample, a technical replicate was included. To allow comparison between data sets, the threshold to determine the quantification cycle (Cq) was set at the same relative fluorescence units (RFU) for all reactions detecting a particular transcript and all qPCR plates were run on the same machine. After each run, the specificity of the qPCR was determined by dissociation curve analysis. The relative transcript level in each sample was determined by normalising to the *actb1* transcript level in that sample.

### Behaviour assay

For the first ~30 h of development, embryos were raised in an incubator under a white LD regime. Thereafter, the larvae were placed in the DanioVision^®^ automated observation chamber (Noldus) under a medium or low intensity blue LD 12:12 h regime with illumination from the top (Fig EV3I) and constant temperature (28°C). One larva was placed in each well (Ø35 mm wells, 8 ml of Aqua Condition Plus KH+ water) of a 6 well plate on the 1st dpf and entrained for 8 days. Note that small wells result in unreliable locomotor activity readout (Wolter & Svoboda, 2020). In each experiment, 3 wild‐type and 3 *opn4* dko larvae were placed next to each other. The larvae were recorded and tracked with EthoVision^®^ software during the 5^th^, 6^th^ and 7^th^ dpf. Their activity or velocity was measured as the rate of change of a larva's position in a unit time. The distance covered by a larva during 6 min was binned. Locomotor activity was measured of 18 larvae (biological replicates) from spawnings of different parents for each experiment. To determine if the activity of wild‐type and mutant larvae in the light or dark phase on a particular day shows a difference that is significant, we pooled all activity bins in the respective phase of each larva separately and then applied the test statistics on the data. The aggregated 12 h activity data of each larva on one particular day is in this case one single observation in the test statistics. As the consecutive days were analysed and presented separately, the assumption of independent identical observations is fulfiled. For the analysis of large‐angle turns, the distance covered in 6 sec was binned for each larva, and the mean distance covered of each larva over the last 6 min in the light phase was used as a baseline to calculate the displacement in the first 6 sec of that larva in darkness (Fig EV3A–F). For routine turns, the mean distance covered of each larva over the last 6 min in the light phase was used as a baseline to calculate the displacement of that larva above this level in the following 12 min of darkness (Fig EV3A–H).

### Test statistics

Statistical analysis of the transcriptome data was implemented with edgeR software (version3.9). For differential expression, edgeR applies an exact test analogous to Fisher’s exact (Fisher, 1922) with consideration of overdispersion and adjusts the *P*‐values for the expected proportion of type I errors (false discovery rate) with the Benjamini‐Hochberg procedure (Benjamini & Hochberg, 1995). The cutoff for differential expression was set at a significance level of α = 0.05. Fisher's exact test was applied to determine if the common differentially expressed genes among two data sets are shared due to coincidence or dependence (https://www.r‐project.org/). For analysis of behaviour and qPCR data, the distribution in a data set was calculated with the Shapiro–Wilk test (Shapiro & Wilk, 1965). For normally distributed data, the unpaired Student *t*‐test (Student, 1908) was applied. To determine if a significant difference between the variances of two normally distributed data sets exists, the *F*‐test was applied. When *H*_0_ is accepted (*P* > 0.05), a *t*‐test with equal variance was applied and when *H*_0_ is rejected, a *t*‐test with Welch's correction was applied (unequal variance). In all cases we analysed if there is a difference between the data sets (two‐tailed). Hypothetically one could apply for the locomotor activity a one‐tailed test, as the aggregated velocity in the light phase of a diurnal vertebrate can be assumed to be less when less photons are detected. In this case, the *P*‐values for locomotor activity in the wake state, which follows a normal distribution, are half of the stated *P*‐values in this report. To determine if the difference between the wild‐type and *opn4* dko locomotor activity is due to interindividual variability or the result of the phenotype, we calculated Cohen's effect size (*d*), which is a comparison of the difference between the means with the pooled standard deviation (Cohen, 1988). Note that this calculation of the effect size can only be applied on data that follows a normal distribution. Cohen defined descriptors for magnitude of effect size as medium: 0.5≤|*d*|≤0.8, or large: |*d*|>0.8. For non‐normally distributed data, the nonparametric Wilcoxon rank‐sum test/Mann–Whitney *U*‐test (Mann & Whitney, 1947) was applied. The latter distribution is indicated with *U*‐test in front of the stated *P*‐value. The significance level was set in all cases at α = 0.05, following standard practice in this field.

## Author contributions

**Marcus P S Dekens:** Conceptualization; Resources; Data curation; Formal analysis; Supervision; Funding acquisition; Validation; Investigation; Visualization; Methodology; Writing—original draft; Project administration; Writing—review & editing. **Bruno M Fontinha:** Investigation. **Miguel Gallach:** Formal analysis. **Sandra Pflügler:** Investigation. **Kristin Tessmar‐Raible:** Conceptualization; Resources; Supervision; Funding acquisition; Project administration; Writing—review & editing.

In addition to the CRediT author contributions listed above, the contributions in detail are:

MPSD and KT‐R conceived and designed the study. MPSD generated the *opn4* knockouts, conducted KEGG and GO analysis on the differentially expressed genes, executed the photobiology experiments and conducted quantitative and statistical analysis of gene expression and behaviour. MPSD and SP performed the *in situ* hybridisations on adult brains. MPSD and BMF executed the transcriptome sequencing in collaboration with the Vienna BioCenter Core Facilities (VBCF). MG processed the raw sequence data. MPSD wrote, and KT‐R reviewed the manuscript. All authors approved the manuscript.

## Supporting information



Appendix

Expanded View Figures PDF

Table EV1

Table EV2

Table EV3

Table EV4

Source Data for Figure 2

## Data Availability

RNA‐Seq data: NCBI Gene Expression Omnibus GSE189906 (https://www.ncbi.nlm.nih.gov/geo/query/acc.cgi?acc=GSE189906).
