# Supplementary material for: Melanopsin elevates locomotor activity during the wake state of the diurnal zebrafish
Source: EMBO Rep. 2022 Mar 1;23(5):e51528. doi: 10.15252/embr.202051528 (PMC9066073; doi:10.15252/embr.202051528)
Supplement: Supplementary file 1 — Appendix [file EMBR-23-e51528-s006.pdf]

Table of contents

Appendix Figure S1

Expression profiling reveals genes downstream of Melanopsin.

(A) Annotated heat map of all over- (red) and underexpressed (green) genes in the *opn4* dko anterior brain with 3 biological replicates for wild-type and 3 for the *opn4* dko. For significance testing an exact test analogous to Fisher's exact with consideration of overdispersion and adjusted p-values for the false discovery rate was applied. The cutoff for differential expression was set at a significance level of α=0.05. Pathways indicated with a blue dot for phototransduction, red for metabolism, orange for melatonin synthesis, green for MAPK, and grey for cAMP signalling. Genes assigned to light-dependend processes (GO) are indicated with a double dagger. Homeobox genes are indicated with komejirushi (※), genes with a function in the immune response with triangle, genes with a function in cell division with a circle and core clock genes with a square.

(B) Heat map of the differentially expressed genes in the *opn4* dko posterior brain.

(C) Heat map of the differentially expressed genes in the *opn4* dko eyes.

(D-V) Plots with read counts normalised to *actb1* for (D) *tph1a,* (E) *tph2,* (F) *aanat2,* (G) *otx5,* (H) *crx,* (I) *lhx4,* (J) *otx2,* (K) *mcm7,* (L) *cdk9,* (M) *caspb,* (N) *ly6pge,* (O) *pigrl3.5,* (P) *mhc1uba,* (Q) *mhc1uka,* (R) *creb3l1,* (S) *cdk5,* (T) *roraa,* (U) *per1a,* and (V) *per2*.

Data information: In D-V, blue markers indicate wild-type, grey markers indicate *opn4* dko and red bar indicates mean. Biological replicates are indicated with round, triangular, and square markers (n=3). Asterisks indicate significance (0.01<p[*]<0.05, 0.001<p[**]<0.01, p[***]<0.001, ns = not significant).

Appendix Figure S2

ddc transcription in wild-type and *opn4* dko larvae.

The *ddc* transcription pattern is unchanged between wild-type and *opn4* dko, but the *ddc* expression level is affected in the *opn4* dko pineal. Dorsal and lateral view of each biological replicate from Figure 4N is shown. *ddc* is expressed in the epiphysis cerebri [E], pretectum [PT], diencephalic catecholaminergic cluster [DCA], raphe nuclei [RN], locus coeruleus [LC], area postrema [AP], and gut. Larvae were raised under a 12:12 hour LD regime and fixed on the 6th dpf at ZT4. Eyes were removed after completion of the *ish* for optimal visibility of expression in the brain.

Appendix Figure S1

A

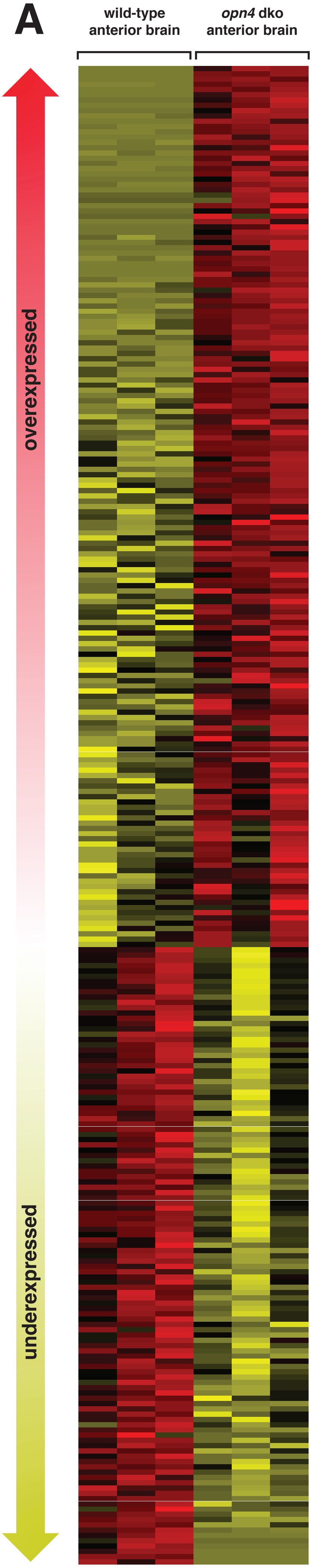

Ensembl gene ID	log2 FC	FDR *p*	Gene name	Gene description
ENSDARG00000020602	9.092376758	6.32E-11	*grk7a* ● ‡	G protein-coupled receptor kinase 7a
ENSDARG00000010958	9.021105055	1.42E-14	*glmnb*	glomulin, FKBP associated protein b
ENSDARG00000010454	8.977883406	2.63E-12	*guca1a* ●	guanylate cyclase activator 1A
ENSDARG00000044684	8.933744322	3.85E-60	*rbp4l* ‡	retinol binding protein 4, like
ENSDARG00000052223	8.686503613	2.32E-11	*rcvrna* ● ‡	recoverin a
ENSDARG00000029898	8.592737258	2.53E-08	*cnga1a*	cyclic nucleotide gated channel alpha 1a
ENSDARG00000011671	8.418096095	1.09E-06	*pde6b* ●	phosphodiesterase 6B, cGMP-specific, rod, beta
ENSDARG00000076512	8.317523362	1.39E-07	-	-
ENSDARG00000019902	8.298781622	1.21E-05	*rcvrn2* ● ‡	recoverin 2
ENSDARG00000008306	8.241282420	9.98E-17	*rdh5*	retinol dehydrogenase 5 (11-cis/9-cis)
ENSDARG00000056938	8.119983448	2.07E-06	*kera* ‡	keratocan
ENSDARG00000037941	7.997407803	1.33E-22	*syt5a*	synaptotagmin Va
ENSDARG00000009637	7.957940174	1.28E-45	*rcvrn3* ‡	recoverin 3
ENSDARG00000017634	7.957166319	4.05E-06	*pdcb*	phosducin b
ENSDARG00000075963	7.841964471	3.46E-185	*mhc1uba* ▲	major histocompatibility complex class I UBA
ENSDARG00000069627	7.675777090	0.000497548	-	si:ch211-255g12.8
ENSDARG00000012610	7.671818838	2.46E-97	*saga* ●	S-antigen; retina and pineal gland (arrestin) a
ENSDARG00000010680	7.533117145	0.000829859	*gngt2a*	guanine nucleotide binding protein (G protein), gamma transducing activity polypeptide 2a
ENSDARG00000041379	7.532100930	1.15E-13	-	si:ch211-245j22.3
ENSDARG00000035798	7.507498235	1.56E-32	*gngt1* ●	guanine nucleotide binding protein (G protein), gamma transducing activity polypeptide 1
ENSDARG00000061044	7.494438118	0.000859675	-	si:dkey-72l14.3
ENSDARG00000004643	7.419025060	0.003061051	*cdhr1a*	cadherin-related family member 1a
ENSDARG00000057239	7.338202057	1.89E-13	*tph2* ●	tryptophan hydroxylase 2 (tryptophan 5-monooxygenase)
ENSDARG00000078244	7.248861565	0.002705424	-	-
ENSDARG00000021959	7.197831115	0.019256362	-	zgc:101100
ENSDARG00000001976	7.187552101	0.043926085	*fkbp16*	FKBP prolyl isomerase 16
ENSDARG00000100858	7.141490770	0.002790205	-	zgc:171695
ENSDARG00000057427	7.132778949	0.010690162	*sv2ba*	synaptic vesicle glycoprotein 2Ba
ENSDARG00000104302	7.069445267	0.042178234	-	-
ENSDARG00000032838	6.905019878	0.011832327	*adcy1* ●	adenylate cyclase type 1 (si:dkey-206f10.1)
ENSDARG00000102288	6.868911565	0.013657151	*pcare*	photoreceptor cilium actin regulator
ENSDARG00000005776	6.735573800	0.026054735	*guk1b* ●	guanylate kinase 1b
ENSDARG00000044199	6.720208461	8.52E-22	*gnat1* ● ‡	guanine nucleotide binding protein (G protein), alpha transducing activity polypeptide 1
ENSDARG00000012126	6.718696856	2.06E-06	-	zgc:109965
ENSDARG00000057064	6.547765410	0.034500938	*enpep*	glutamyl aminopeptidase
ENSDARG00000038587	6.499969748	0.034312044	*LOC100004199*	uncharacterized LOC100004199
ENSDARG00000011989	6.499100266	2.69E-13	*crx* ‡ ※	cone-rod homeobox
ENSDARG00000087349	6.364975166	0.043409069	*reep6*	receptor accessory protein 6
ENSDARG00000092731	6.364483245	0.041724532	*mhc1uka* ▲	major histocompatibility complex class I UKA
ENSDARG00000101485	6.345959109	0.043409069	-	-
ENSDARG00000010231	6.211003730	1.92E-06	*ppm1na* ●	protein phosphatase, Mg2+/Mn2+ dependent, 1Na (putative)
ENSDARG00000056511	5.878932674	1.02E-26	*arr3a* ● ‡	arrestin 3a, retinal (X-arrestin)
ENSDARG00000039458	5.875749383	0.003400434	*lhx4* ※	LIM homeobox 4
ENSDARG00000052039	5.824896766	0.000490293	*caspb* ▲	caspase b
ENSDARG00000098249	5.762725944	4.74E-38	*asmt* ●	acetylserotonin O-methyltransferase
ENSDARG00000098392	5.757760065	3.10E-09	*ly6pge* ▲	lymphocyte antigen 6 family member pge
ENSDARG00000059257	5.573027035	1.29E-46	*rcvrnb*	recoverin b
ENSDARG00000093753	5.572591741	3.33E-34	-	-
ENSDARG00000077622	5.568627518	6.99E-13	*LOC101883296*	solute carrier family 35 member F4-like
ENSDARG00000038378	5.360155584	2.09E-09	*sagb*	S-antigen; retina and pineal gland (arrestin) b
ENSDARG00000095802	5.351370750	4.79E-10	-	si:ch1073-155h21.2
ENSDARG00000025504	5.173217269	5.98E-07	*gucy2f* ● ‡	guanylate cyclase 2F, retinal
ENSDARG00000102393	4.982321783	0.005990909	*arl3l1*	ADP-ribosylation factor-like 3, like 1
ENSDARG00000095831	4.858497707	0.043409069	*LOC556653*	uncharacterized LOC556653
ENSDARG00000076371	4.612152650	0.002974990	*shroom2b*	shroom family member 2b
ENSDARG00000098890	4.558438888	0.042297452	*cyp2aa9*	cytochrome P450, family 2, subfamily AA, polypeptide 9
ENSDARG00000097813	4.494908999	0.029068968	-	-
ENSDARG00000003899	4.437082479	0.034721319	*zdhhc23b*	zinc finger, DHHC-type containing 23b
ENSDARG00000007715	4.421506137	0.034613085	*lgsn*	lengsin, lens protein with glutamine synthetase domain
ENSDARG00000025076	4.349170241	0.018530842	*marveld2a* ‡	MARVEL domain containing 2a
ENSDARG00000091834	4.349146061	9.51E-09	-	si:ch211-198m17.1
ENSDARG00000029432	4.251315202	7.89E-07	*tph1a* ●	tryptophan hydroxylase 1 (tryptophan 5-monooxygenase) a
ENSDARG00000000380	4.183743863	3.25E-18	*pde6a* ●	phosphodiesterase 6A, cGMP-specific, rod, alpha
ENSDARG00000044672	4.151774636	1.23E-06	*pp2* ‡	parapinopsin b
ENSDARG00000101828	4.129519529	0.006455874	-	-
ENSDARG00000045898	3.990238598	0.033380002	-	-
ENSDARG00000059163	3.980038388	6.87E-15	*rbp3* ‡	retinol binding protein 3
ENSDARG00000033760	3.910634917	1.61E-07	*pmelb*	premelanosome protein b
ENSDARG00000012504	3.784354661	0.000829859	*rlbp1a* ‡	retinaldehyde binding protein 1a
ENSDARG00000094433	3.755197872	0.046162231	*caspbl* ▲	caspase b, like
ENSDARG00000096519	3.748625187	0.011915855	*irbpl* ‡	interphotoreceptor retinoid-binding protein like
ENSDARG00000043483	3.683294302	0.001199014	*otx5* ※	orthodenticle homolog 5
ENSDARG00000004459	3.669862442	0.000326388	*unc119.2* ‡	unc-119 lipid binding chaperone B homolog 2
ENSDARG00000007682	3.629096299	5.07E-16	*ppdpfa*	pancreatic progenitor cell differentiation and proliferation factor a
ENSDARG00000086077	3.618926473	0.031788424	-	-
ENSDARG00000096964	3.534655012	0.014648844	-	-
ENSDARG00000101984	3.443198024	3.71E-11	*pde6gb* ● ‡	phosphodiesterase 6G, cGMP-specific, rod, gamma, paralog b
ENSDARG00000055534	3.342074515	0.047132677	*grk7b* ● ‡	G protein-coupled receptor kinase 7b
ENSDARG00000041431	3.204089995	6.47E-09	*slc24a1* ●	solute carrier family 24 member 1
ENSDARG00000076547	3.120524809	7.57E-08	-	wu:fb13g09
ENSDARG00000074518	3.001427532	0.011082065	*tmem17*	transmembrane protein 17
ENSDARG00000007382	2.999487460	0.005990909	*ubtd1a*	ubiquitin domain containing 1a
ENSDARG00000099406	2.955712811	6.27E-05	*lrit1b*	leucine-rich repeat, immunoglobulin-like and transmembrane domains 1b
ENSDARG00000104007	2.941032718	7.70E-05	*pcdh1g1*	protocadherin 1 gamma 1
ENSDARG00000103324	2.831363497	8.93E-06	-	-
ENSDARG00000088795	2.830708085	0.029068968	*engl*	endoglin, like
ENSDARG00000105592	2.805290466	9.87E-05	-	LincRNA type
ENSDARG00000016494	2.785037644	4.05E-11	*ddc* ●	dopa decarboxylase
ENSDARG00000007480	2.783574503	9.44E-15	*rpe65a* ‡	retinoid isomerohydrolase RPE65 a
ENSDARG00000078522	2.758063402	3.28E-07	-	si:ch211-80h18.1
ENSDARG00000007099	2.746840850	0.043409069	*cx43.4*	connexin 43.4
ENSDARG00000076972	2.599451258	0.004345547	*plvapa*	plasmalemma vesicle associated protein a
ENSDARG00000057169	2.544855273	0.001992180	*abca4a*	ATP-binding cassette, sub-family A (ABC1), member 4a
ENSDARG00000076134	2.494478716	0.043649291	-	-
ENSDARG00000051874	2.412104856	1.14E-05	*stra6* ‡	stimulated by retinoic acid 6
ENSDARG00000026820	2.400931333	0.003360770	*gucy2d* ●	guanylate cyclase 2D, retinal
ENSDARG00000039490	2.337923911	0.002729044	*pitpnaa*	phosphatidylinositol transfer protein, alpha a
ENSDARG00000038703	2.200530326	0.003546941	*hkdc1* ●	hexokinase domain containing 1
ENSDARG00000102176	2.176678444	0.000299249	*pla1a*	phospholipase A1 member A
ENSDARG00000022832	2.154516939	0.007237316	*bnip4*	BCL2 interacting protein 4
ENSDARG00000069478	2.132944041	0.000154932	*slc13a3*	solute carrier family 13 member 3
ENSDARG00000027495	2.072174537	0.042279940	*elovl4b* ●	ELOVL fatty acid elongase 4b
ENSDARG00000100277	2.039694153	0.002079135	-	-
ENSDARG00000103256	2.034741564	0.002921616	*pcdh2g1*	protocadherin 2 gamma 1
ENSDARG00000011235	1.985424111	0.004462324	*otx2b* ※	orthodenticle homeobox 2b
ENSDARG00000054343	1.952134493	6.27E-05	*slc7a8b*	solute carrier family 7 member 8b
ENSDARG00000098587	1.883524395	0.042279940	*LOC101885013*	uncharacterized LOC101885013
ENSDARG00000070683	1.867583978	0.042297452	*dkk3b*	dickkopf WNT signaling pathway inhibitor 3b
ENSDARG00000079802	1.863957090	0.044872776	*aanat2* ● ‡	arylalkylamine N-acetyltransferase 2
ENSDARG00000090942	1.863090802	0.034500938	*LOC101885996*	gastrula zinc finger protein XlCGF26.1-like
ENSDARG00000045638	1.757696037	0.004981932	*slc13a1*	solute carrier family 13 member 1
ENSDARG00000038713	1.753107448	0.011915855	*tep1*	telomerase-associated protein 1
ENSDARG00000028053	1.739310658	4.27E-06	*bmp1b*	bone morphogenetic protein 1b
ENSDARG00000032849	1.723673848	4.25E-09	*ndrg1a*	N-myc downstream regulated 1a
ENSDARG00000069583	1.709983787	0.004981932	-	zgc:114181
ENSDARG00000010729	1.677669723	0.011029121	*LOC795887*	interferon-induced protein 44
ENSDARG00000034650	1.673884709	0.028946021	*fabp7b*	fatty acid binding protein 7, brain, b
ENSDARG00000074248	1.652770790	0.035055543	*tmem237b*	transmembrane protein 237b
ENSDARG00000019713	1.645581925	0.000757057	*oatx*	organic anion transporter X
ENSDARG00000091756	1.586063529	0.004129805	*insm1a*	insulinoma-associated 1a
ENSDARG00000003251	1.552900838	0.011029121	*zbtb22b*	zinc finger and BTB domain containing 22b
ENSDARG00000069525	1.537466058	0.033778139	*gpn3*	GPN-loop GTPase 3
ENSDARG00000000729	1.407615256	0.005944664	*daxx* ●	death-domain associated protein
ENSDARG00000104387	1.362564202	0.000568806	*slc4a5*	solute carrier family 4 member 5
ENSDARG00000099108	1.352663407	0.021938659	*LOC100333907*	chloride intracellular channel protein 4-like
ENSDARG00000102818	1.310254218	0.014459057	-	LincRNA type
ENSDARG00000045611	1.299762053	0.000757057	*nfybb*	nuclear transcription factor Y, beta b
ENSDARG00000070919	1.229979699	0.048603079	*cpne5b*	copine Vb
ENSDARG00000101481	1.226223245	0.005487110	*rbp5*	retinol binding protein 1a, cellular
ENSDARG00000061140	1.225030508	0.008179205	*colec12*	collectin sub-family member 12
ENSDARG00000055290	1.223632730	0.048738782	*mpeg1.1* ▲	macrophage expressed 1, tandem duplicate 1
ENSDARG00000099781	1.183757850	0.001206738	*asip2b*	agouti signaling protein, nonagouti homolog (mouse) 2b
ENSDARG00000103957	1.151783812	0.042044649	*aamdc*	adipogenesis associated, Mth938 domain containing
ENSDARG00000061417	1.151330108	0.003546941	*nub1*	negative regulator of ubiquitin-like proteins 1
ENSDARG00000077023	1.134834200	0.000108163	*pcdhb*	protocadherin b
ENSDARG00000078322	1.095858496	0.012456350	*col12a1a*	collagen, type XII, alpha 1a
ENSDARG00000056510	1.083479856	0.043409069	*gstk1*	glutathione S-transferase kappa 1
ENSDARG00000101199	1.043131856	0.000131708	*rbp4* ‡	retinol binding protein 4, plasma
ENSDARG00000059400	1.033033391	0.026054735	*tmem167b*	transmembrane protein 167B
ENSDARG00000037403	1.032737329	0.003543224	-	si:dkey-4p15.3
ENSDARG00000063466	1.021036670	4.38E-09	*isy1*	ISY1 splicing factor homolog
ENSDARG00000005191	1.019413576	0.018084503	*ahcy* ●	adenosylhomocysteinase
ENSDARG00000071437	1.018455231	0.019256362	*ptprc*	protein tyrosine phosphatase receptor type C
ENSDARG00000001015	0.943350582	0.002119858	*znf384l*	zinc finger protein 384 like
ENSDARG00000058966	0.938173950	0.001700520	-	zgc:112332
ENSDARG00000021383	0.920667766	0.032111151	*naalad2*	N-acetylated alpha-linked acidic dipeptidase 2
ENSDARG00000004211	0.898069724	0.042297452	*ccdc106a*	coiled-coil domain containing 106a
ENSDARG00000076473	0.887248274	6.97E-05	*trim37*	tripartite motif containing 37
ENSDARG00000001710	0.881172763	2.32E-06	*flot1a*	flotillin 1a
ENSDARG00000073881	0.877727567	0.042297452	*slc41a2b*	solute carrier family 41 member 2b
ENSDARG00000069194	0.864694275	0.038194161	*mycbpap*	mycbp associated protein
ENSDARG00000026383	0.804096892	0.015850732	-	si:dkey-27j5.5
ENSDARG00000010434	0.794661987	0.001464344	*clu*	clusterin
ENSDARG00000010008	0.787195540	0.00478598	*vim*	vimentin
ENSDARG00000016141	0.786804752	0.016594269	*slc6a2*	solute carrier family 6 member 2
ENSDARG00000027290	0.763733347	0.027297066	*nrp1b* ▲	neuropilin 1b
ENSDARG00000001929	0.732206150	0.049556653	*pink1*	PTEN induced kinase 1
ENSDARG00000077737	0.730186719	0.029848134	*spsb3a*	splA/ryanodine receptor domain and SOCS box containing 3a
ENSDARG00000056080	0.723309655	0.044599810	-	si:dkey-191g9.5
ENSDARG00000013144	0.718259675	0.043941165	*atp1b1a* ●	ATPase Na+/K+ transporting subunit beta 1a
ENSDARG00000097196	0.686649465	0.000118429	-	LincRNA type
ENSDARG00000015128	0.652914251	0.005438357	*rpl27*	ribosomal protein L27
ENSDARG00000014817	0.647203015	0.014621129	*ranbp1*	RAN binding protein 1
ENSDARG00000018637	0.642030301	0.025957342	*sec61g*	Sec61 translocon gamma subunit
ENSDARG00000099005	0.637195222	0.027297066	*palm2*	paralemmin 2
ENSDARG00000044062	0.492147198	0.026676976	*ctbp2a*	C-terminal binding protein 2a
ENSDARG00000095556	0.478488974	0.044599810	*LOC795545*	uncharacterized LOC795545
ENSDARG00000062056	-0.479855776	0.030378228	*elmod1*	ELMO/CED-12 domain containing 1
ENSDARG00000074521	-0.485304446	0.042297452	*map6a*	microtubule-associated protein 6a
ENSDARG00000079000	-0.496786632	0.012922230	*tp53bp1*	tumor protein p53 binding protein, 1
ENSDARG00000057484	-0.516541126	0.017708538	*srsf2a*	serine and arginine rich splicing factor 2a
ENSDARG00000038442	-0.518671381	0.018535586	*plcg1* ●	phospholipase C, gamma 1
ENSDARG00000101054	-0.523287895	0.011039021	*syn2b*	synapsin IIb
ENSDARG00000032865	-0.523691977	0.019829243	*pacsin1a*	protein kinase C and casein kinase substrate in neurons 1a
ENSDARG00000104279	-0.555868213	0.002287605	*appa*	amyloid beta (A4) precursor protein a
ENSDARG00000079949	-0.560909570	0.018856599	*supt16h*	SPT16 homolog, facilitates chromatin remodeling subunit
ENSDARG00000100487	-0.569279351	0.040003732	*nploc4*	NPL4 homolog, ubiquitin recognition factor
ENSDARG00000038228	-0.577061917	0.045307014	*selenow2a*	selenoprotein W, 2a
ENSDARG00000088708	-0.607294909	0.041724532	*ngfra* ●	nerve growth factor receptor a (tumor necrosis factor receptor superfamily, member 16)
ENSDARG00000074381	-0.639552288	0.008153483	*farp1*	FERM, RhoGEF (ARHGEF) and pleckstrin domain protein 1 (chondrocyte-derived)
ENSDARG00000057322	-0.665405616	0.033380002	*tmem47*	transmembrane protein 47
ENSDARG00000078113	-0.667283263	0.030209743	*atp5mea* ●	ATP synthase membrane subunit ea
ENSDARG00000100789	-0.669497834	0.020709637	*plgrkt*	plasminogen receptor, C-terminal lysine transmembrane protein
ENSDARG00000056877	-0.670449656	0.007424349	*vamp2*	vesicle-associated membrane protein 2
ENSDARG00000010031	-0.674018093	0.009997374	*ssh1b*	slingshot protein phosphatase 1b
ENSDARG00000102191	-0.675798116	0.026178983	*LOC101886030*	copine-8
ENSDARG00000004171	-0.685191705	0.029035855	*rabac1*	Rab acceptor 1 (prenylated)
ENSDARG00000062765	-0.699042676	0.001835344	*nsd3*	nuclear receptor binding SET domain protein 3
ENSDARG00000101413	-0.702186451	0.002060553	*dusp7* ●	dual specificity phosphatase 7
ENSDARG00000078133	-0.703119935	0.014386820	*kdm2ab*	lysine (K)-specific demethylase 2Ab
ENSDARG00000026519	-0.715634176	0.033418238	*ralgps2*	Ral GEF with PH domain and SH3 binding motif 2
ENSDARG00000104375	-0.728935767	0.047213520	*sec24a*	SEC24 homolog A, COPII coat complex component
ENSDARG00000103845	-0.729264063	0.001968719	-	zgc:55733
ENSDARG00000104665	-0.738767932	0.025701114	*kirrel3l*	kirre like nephrin family adhesion molecule 3, like
ENSDARG00000061752	-0.747178292	0.000778994	*ripor2* ‡	RHO family interacting cell polarization regulator 2
ENSDARG00000104582	-0.765161508	0.001700520	-	-
ENSDARG00000005397	-0.779505807	0.000207104	*trim3b*	tripartite motif containing 3b
ENSDARG00000031382	-0.780438207	0.000139309	*reep2*	receptor accessory protein 2
ENSDARG00000058256	-0.781581466	0.005944664	*draxin*	dorsal inhibitory axon guidance protein
ENSDARG00000057881	-0.783082076	0.002711323	-	si:dkey-33c12.3
ENSDARG00000077174	-0.790753864	0.016662075	*qsox2*	quiescin Q6 sulfhydryl oxidase 2
ENSDARG00000068434	-0.795063319	0.033195353	*h3f3b.1*	H3 histone, family 3B.1
ENSDARG00000067850	-0.799321267	0.048123907	*jund* ●	JunD proto-oncogene, AP-1 transcription factor subunit
ENSDARG00000104458	-0.809513816	3.81E-05	*rnasekb*	ribonuclease, RNase K b
ENSDARG00000058270	-0.813120256	0.038246367	-	si:ch211-114l13.7
ENSDARG00000077228	-0.813897445	0.002790205	*ntrk3a*	neurotrophic tyrosine kinase, receptor, type 3a
ENSDARG00000075385	-0.817770613	0.016398950	-	-
ENSDARG00000103333	-0.821336854	0.016782475	*baiap2b*	BAI1-associated protein 2b
ENSDARG00000105323	-0.823051541	8.53E-05	-	-
ENSDARG00000087364	-0.828706726	0.034500938	-	-
ENSDARG00000030665	-0.838854426	0.018414456	*tcerg1b*	transcription elongation regulator 1b
ENSDARG00000018653	-0.850764395	0.001245736	*acot7*	acyl-CoA thioesterase 7
ENSDARG00000061100	-0.851306335	0.000750159	*nars*	asparaginyl-tRNA synthetase
ENSDARG00000059150	-0.880042907	3.67E-05	*tmem107*	transmembrane protein 107
ENSDARG00000099240	-0.881748921	7.10E-08	*acap3b*	ArfGAP with coiled-coil, ankyrin repeat and PH domains 3b
ENSDARG00000105587	-0.902421932	0.030209743	-	LincRNA type
ENSDARG00000103844	-0.913482878	0.002119858	-	-
ENSDARG00000011921	-0.928432178	9.42E-05	*txnl1*	thioredoxin-like 1
ENSDARG00000098176	-0.944571969	0.000118663	-	si:rp71-1h20.5
ENSDARG00000074506	-0.959225423	9.47E-05	*tmem94*	transmembrane protein 94
ENSDARG00000011170	-0.973012054	2.43E-07	*ndrg2*	NDRG family member 2
ENSDARG00000063019	-0.991630598	0.016496134	*panx2*	pannexin 2
ENSDARG00000003835	-0.995156143	7.74E-05	*stom*	stomatin
ENSDARG00000097937	-1.007072526	0.013191071	-	-
ENSDARG00000104949	-1.010959481	0.001199014	*slc27a1b*	solute carrier family 27 member 1b
ENSDARG00000100409	-1.020945221	4.11E-05	*camlg*	calcium modulating ligand
ENSDARG00000103740	-1.025462712	1.48E-06	*fundc2*	fun14 domain containing 2
ENSDARG00000043905	-1.030878847	0.048663476	*rnf14*	ring finger protein 14
ENSDARG00000104280	-1.035891597	0.035559797	*bnip1b*	BCL2 interacting protein 1b
ENSDARG00000101334	-1.040089031	0.026054735	*ctsc*	cathepsin C
ENSDARG00000018825	-1.06453777	0.011744939	*tmem248*	transmembrane protein 248
ENSDARG00000008542	-1.090800809	0.021434308	*yod1*	YOD1 deubiquitinase
ENSDARG00000101243	-1.178650632	0.002838787	-	-
ENSDARG00000104808	-1.188166052	6.22E-05	*jak2a*	Janus kinase 2a
ENSDARG00000071086	-1.226241016	0.001367215	*gpr155b*	G protein-coupled receptor 155b
ENSDARG00000056566	-1.232570952	2.17E-05	*necab1*	N-terminal EF-hand calcium binding protein 1
ENSDARG00000090232	-1.252101557	0.000415813	*clpb*	ClpB homolog, mitochondrial AAA ATPase chaperonin
ENSDARG00000015793	-1.300570914	0.027626384	*creb3l1* ●	cAMP responsive element binding protein 3-like 1
ENSDARG00000090634	-1.312578812	0.043409069	*smim29*	small integral membrane protein 29
ENSDARG00000091956	-1.330099006	0.008153483	*tmem184bb*	transmembrane protein 184bb
ENSDARG00000077567	-1.332822886	0.016029342	*angel1*	angel homolog 1 (Drosophila)
ENSDARG00000103186	-1.336073084	0.014701302	-	si:ch73-389k6.1
ENSDARG00000052113	-1.350440666	0.003337709	*hexa* ●	hexosaminidase A (alpha polypeptide)
ENSDARG00000098860	-1.384070752	0.047132677	*fam57a*	family with sequence similarity 57 member A
ENSDARG00000076299	-1.385017209	0.025708049	-	-
ENSDARG00000036966	-1.397265357	0.003360770	*hsd3b7* ●	hydroxy-delta-5-steroid dehydrogenase, 3 beta- and steroid delta-isomerase
ENSDARG00000061024	-1.417142137	2.80E-07	*rabggta*	Rab geranylgeranyltransferase subunit alpha
ENSDARG00000099534	-1.471943877	0.000757057	*LOC100329344*	putative E3 ubiquitin-protein ligase UBR7
ENSDARG00000019001	-1.479183706	5.16E-05	*ak7a*	adenylate kinase 7a
ENSDARG00000076518	-1.515140594	0.011039021	*mettl1*	methyltransferase like 1
ENSDARG00000090202	-1.553508954	0.042297452	*rbm11*	RNA binding motif protein 11
ENSDARG00000032650	-1.592399043	0.038639414	*fuk* ●	fucokinase
ENSDARG00000089844	-1.609257330	0.042297452	*scarb2c*	scavenger receptor class B, member 2c
ENSDARG00000104217	-1.620063670	0.012456350	*gpc2*	glypican 2
ENSDARG00000077178	-1.630680987	0.007237316	-	zgc:152977
ENSDARG00000102067	-1.636839570	0.003360770	*grm5b*	glutamate receptor, metabotropic 5b
ENSDARG00000087762	-1.658543889	9.87E-08	*rab27b*	RAB27B, member RAS oncogene family
ENSDARG00000030964	-1.661351607	0.014976060	*pde12*	phosphodiesterase 12
ENSDARG00000044811	-1.681724930	3.45E-11	*cdk9* ○	cyclin-dependent kinase 9 (CDC2-related kinase)
ENSDARG00000004889	-1.777242779	0.011513646	*aga*	aspartylglucosaminidase
ENSDARG00000056871	-1.870201849	3.99E-10	*ap4m1*	adaptor related protein complex 4 subunit mu 1
ENSDARG00000104524	-1.963039127	0.012395041	*LOC101885237* ▲	macrophage mannose receptor 1-like (*mrc1*)
ENSDARG00000008142	-1.969800436	1.11E-05	*stxbp3*	syntaxin binding protein 3
ENSDARG00000089863	-1.995305413	0.018535586	*ccdc57*	coiled-coil domain containing 57
ENSDARG00000105450	-2.094475262	1.32E-17	-	si:ch211-63p21.1
ENSDARG00000070579	-2.112850040	0.000598624	*ggact.3*	gamma-glutamylamine cyclotransferase, tandem duplicate 3
ENSDARG00000101180	-2.139603694	2.15E-13	*mcm7* ○	minichromosome maintenance complex component 7
ENSDARG00000092208	-2.320260132	3.31E-10	-	LincRNA type
ENSDARG00000069463	-2.355760740	0.029776413	*alox12* ●	arachidonate 12-lipoxygenase
ENSDARG00000088689	-2.443598478	0.018856599	-	si:ch211-201o1.1
ENSDARG00000038475	-2.594766901	0.000300140	*acy1*	aminoacylase 1
ENSDARG00000093803	-2.781361039	0.000176437	-	-
ENSDARG00000101623	-3.194621774	4.60E-07	*znf1109*	zinc finger protein 1109
ENSDARG00000096000	-3.753344763	0.006371032	-	-
ENSDARG00000063186	-3.766762290	1.52E-16	-	si:ch211-220f12.4
ENSDARG00000022841	-3.876949550	3.15E-13	*ptk2ba*	protein tyrosine kinase 2 beta, a
ENSDARG00000102095	-4.325820152	0.012868912	-	-
ENSDARG00000103131	-4.385223079	1.33E-05	*VWA5A*	von Willebrand factor A domain containing 5A
ENSDARG00000103521	-6.364520309	0.047132677	*capsla*	calcyphosine-like a
ENSDARG00000099938	-6.641683553	0.042297452	*pigrl3.5* ▲	polymeric immunoglobulin receptor-like 3.5
ENSDARG00000097715	-6.652666065	0.026118315	-	-
ENSDARG00000068991	-6.673006351	0.016462320	-	si:dkey-23c22.6
ENSDARG00000098976	-6.967229243	0.004840575	*LOC100535115*	uncharacterized LOC100535115
ENSDARG00000098384	-7.123487234	0.005529361	*znf1101*	zinc finger protein 1101

B

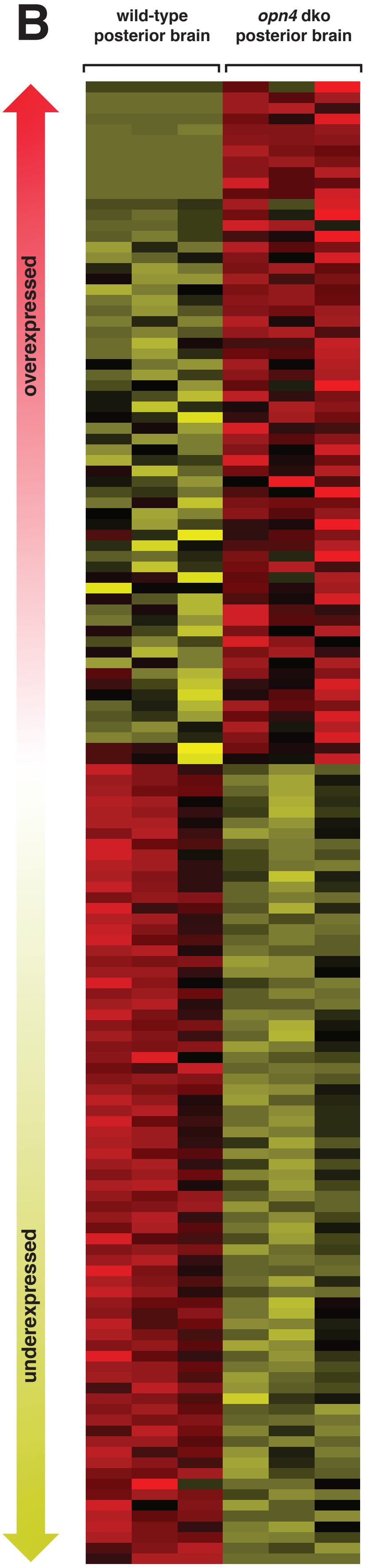

Ensembl gene ID	log2 FC	FDR *p*	Gene name	Gene description
ENSDARG00000044199	8.706654439	0.004842658	*gnat1* ●	guanine nucleotide binding protein (G protein)
ENSDARG00000052039	8.608328715	4.89E-09	*caspb* ▲	caspase b
ENSDARG00000101828	8.122752389	3.43E-05	-	-
ENSDARG00000087706	7.826723409	0.000767557	*dicp3.3* ▲	diverse immunoglobulin domain-containing protein 3.3
ENSDARG00000075963	7.639764509	7.89E-210	*mhc1uba* ▲	major histocompatibility complex class I UBA
ENSDARG00000091253	7.608120380	0.000433212	*smyd1b*	SET and MYND domain containing 1b (histone methyltransferase)
ENSDARG00000098890	7.472871083	0.001541569	*cyp2aa9*	cytochrome P450, family 2, subfamily AA, polypeptide 9
ENSDARG00000071667	7.329725612	0.003280076	-	si:dkey-222f2.7
ENSDARG00000004459	7.307417554	0.003962031	*unc119.2*	unc-119 lipid binding chaperone B homolog 2
ENSDARG00000063359	6.851564185	0.037668838	-	-
ENSDARG00000039422	6.660068170	0.048695324	*fuom*	fucose mutarotase
ENSDARG00000008306	6.400965862	0.017174023	*rdh5*	retinol dehydrogenase 5 (11-cis/9-cis)
ENSDARG00000098249	5.920533880	0.000544304	*asmt* ●	acetylserotonin O-methyltransferase
ENSDARG00000100900	5.787348793	0.015402583	-	-
ENSDARG00000039490	4.771756018	0.015402583	*pitpnaa*	phosphatidylinositol transfer protein, alpha a
ENSDARG00000045827	4.134316167	0.015140048	*lyrm5b*	LYR motif containing 5b
ENSDARG00000012610	3.924838962	0.004203160	*saga* ●	S-antigen; retina and pineal gland (arrestin) a
ENSDARG00000096906	3.699459296	0.046767231	-	si:dkey-7i4.24
ENSDARG00000095820	3.541120638	0.025823972	*LOC100536946*	uncharacterized LOC100536946, Gene Type: ncRNA
ENSDARG00000103324	3.346453079	0.004114475	-	-
ENSDARG00000001303	3.060215177	1.25E-06	*psmb8a*	proteasome subunit beta 8A
ENSDARG00000091754	2.619741936	0.020020466	*chchd5*	coiled-coil-helix-coiled-coil-helix domain containing 5
ENSDARG00000071212	2.510199809	0.013803804	*p3h1*	prolyl 3-hydroxylase 1
ENSDARG00000010729	2.146267362	9.14E-10	*LOC795887*	interferon-induced protein 44
ENSDARG00000089362	2.053886535	0.003274852	*grn1*	granulin 1
ENSDARG00000089043	1.794505544	0.000780670	*ptpn6*	protein tyrosine phosphatase non-receptor type 6
ENSDARG00000058966	1.682832794	2.90E-09	-	zgc:112332
ENSDARG00000071437	1.680832718	3.24E-07	*ptprc*	protein tyrosine phosphatase receptor type C
ENSDARG00000007682	1.569836060	0.048426581	*ppdpfa*	pancreatic progenitor cell differentiation and proliferation factor a
ENSDARG00000090194	1.528322052	0.019603602	*lamtor5* ●	late endosomal/lysosomal adaptor, MAPK and MTOR activator 5
ENSDARG00000036787	1.522567345	0.037383858	*tap2a* ▲	transporter associated with antigen processing, subunit type a
ENSDARG00000087012	1.459713622	0.031403242	-	-
ENSDARG00000011094	1.450303937	0.040492355	*ccna2* ○	cyclin A2
ENSDARG00000067797	1.447969703	0.000767557	*spi1a*	Spi-1 proto-oncogene a
ENSDARG00000045611	1.399277117	0.002015646	*nfybb*	nuclear transcription factor Y, beta b
ENSDARG00000043102	1.398655849	0.008868255	*lxn*	latexin
ENSDARG00000020232	1.381409300	0.016679396	*eif6*	eukaryotic translation initiation factor 6
ENSDARG00000105592	1.376007500	0.000675303	-	-
ENSDARG00000063466	1.174736198	0.000138914	*isy1*	ISY1 splicing factor homolog
ENSDARG00000061417	1.149253526	0.004494490	*nub1*	negative regulator of ubiquitin-like proteins 1
ENSDARG00000003867	1.147877678	0.037778287	*cdk20* ○	cyclin-dependent kinase 20
ENSDARG00000001015	1.117539648	0.003989232	*znf384l*	zinc finger protein 384 like
ENSDARG00000076473	1.063844034	0.010004031	*trim37*	tripartite motif containing 37
ENSDARG00000063624	1.009856366	0.023547397	*gfm1*	G elongation factor, mitochondrial 1
ENSDARG00000055504	0.996290297	0.045280108	-	si:ch211-212k18.7
ENSDARG00000023188	0.977178717	0.035981074	*lcp1* ▲	lymphocyte cytosolic protein 1 (L-plastin)
ENSDARG00000040528	0.974199146	0.028762901	*lgals3bpb*	lectin, galactoside-binding, soluble, 3 binding protein b
ENSDARG00000031776	0.877799708	0.047304254	*fthl27*	ferritin, heavy polypeptide-like 27
ENSDARG00000009753	0.869279911	0.015280802	*sf3b6*	splicing factor 3b, subunit 6
ENSDARG00000098293	0.868376593	0.017174023	-	si:dkey-27i16.2
ENSDARG00000095556	0.848931055	0.000361487	*LOC795545*	uncharacterized LOC795545, Gene Type: ncRNA
ENSDARG00000077257	0.836015260	0.002494086	*timd4* ▲	T cell immunoglobulin and mucin domain containing 4
ENSDARG00000036628	0.824859364	0.015107598	*cd74b* ▲	CD74 molecule, major histocompatibility complex, class II
ENSDARG00000069421	0.805961827	0.023008655	*grpel2*	GrpE-like 2, mitochondrial
ENSDARG00000055791	0.798344650	0.005479451	-	zgc:158423
ENSDARG00000042791	0.756737184	0.041779406	*git2b*	G protein-coupled receptor kinase interacting ArfGAP 2b
ENSDARG00000097196	0.731661684	0.001104878	-	-
ENSDARG00000100270	0.721214625	0.048426581	-	-
ENSDARG00000043697	0.642695907	0.032657951	*nefmb*	neurofilament, medium polypeptide b
ENSDARG00000006546	0.635805299	0.045589523	*ak4* ●	adenylate kinase 4
ENSDARG00000053136	0.625023034	0.047278309	*b2m* ▲	beta-2-microglobulin
ENSDARG00000007098	0.618458375	0.039201604	*itm2ba*	integral membrane protein 2Ba
ENSDARG00000071026	0.576363256	0.040434955	*mllt11*	MLLT11 transcription factor 7 cofactor
ENSDARG00000057853	0.533515417	0.028920891	*atp6v0ca* ●	ATPase H+ transporting V0 subunit ca
ENSDARG00000101054	-0.533110075	0.045280108	*syn2b*	synapsin Iib
ENSDARG00000104279	-0.549696566	0.037383858	*appa*	amyloid beta (A4) precursor protein a
ENSDARG00000079610	-0.569535859	0.027300965	*akap9*	A kinase (PRKA) anchor protein 9
ENSDARG00000005397	-0.611952534	0.027257238	*trim3b*	tripartite motif containing 3b
ENSDARG00000078904	-0.630256329	0.047304254	*brd4*	bromodomain containing 4
ENSDARG00000061066	-0.630796139	0.038926557	*c2cd2l*	c2cd2-like
ENSDARG00000104101	-0.640799750	0.041779406	-	si:ch73-287m6.1
ENSDARG00000089505	-0.663293482	0.023149737	*bod1l1*	biorientation of chromosomes in cell division 1-like 1
ENSDARG00000062346	-0.665637428	0.045280108	*cacna1e* ●	calcium voltage-gated channel subunit alpha1 E
ENSDARG00000100789	-0.670448766	0.023158171	*plgrkt*	plasminogen receptor, C-terminal lysine transmembrane protein
ENSDARG00000074381	-0.670793291	0.024751983	*farp1*	FERM, RhoGEF (ARHGEF) and pleckstrin domain protein 1
ENSDARG00000077228	-0.702465258	0.037383858	*ntrk3a*	neurotrophic tyrosine kinase, receptor, type 3a
ENSDARG00000061100	-0.705142497	0.016871964	*nars*	asparaginyl-tRNA synthetase
ENSDARG00000031768	-0.724617928	0.036478649	*roraa* ■	RAR-related orphan receptor A, paralog a
ENSDARG00000059093	-0.727193482	0.027610721	*ank1a*	ankyrin 1, erythrocytic a
ENSDARG00000090747	-0.732267905	0.046767231	-	si:ch73-269m14.2
ENSDARG00000062744	-0.735471136	0.004764247	*scn1lab*	sodium channel, voltage-gated, type I like, alpha b
ENSDARG00000061909	-0.761165390	0.021234302	-	si:ch73-233f7.1
ENSDARG00000078133	-0.790833671	0.037810491	*kdm2ab*	lysine (K)-specific demethylase 2Ab
ENSDARG00000056877	-0.811961777	0.004114475	*vamp2*	vesicle-associated membrane protein 2
ENSDARG00000068274	-0.835145349	0.017174023	-	si:ch211-203b8.6
ENSDARG00000104708	-0.852808995	0.045280108	*ddx24*	DEAD (Asp-Glu-Ala-Asp) box helicase 24
ENSDARG00000103740	-0.856902683	0.001609481	*fundc2*	fun14 domain containing 2
ENSDARG00000102171	-0.880704925	0.005108502	*fnip1*	folliculin interacting protein 1
ENSDARG00000103962	-0.882298159	0.001609481	*add1*	adducin 1 (alpha)
ENSDARG00000031382	-0.905206302	0.001731688	*reep2*	receptor accessory protein 2
ENSDARG00000097937	-0.934191335	0.019672072	-	-
ENSDARG00000100473	-0.941325221	0.047304254	-	-
ENSDARG00000057881	-0.947029724	0.009207545	-	si:dkey-33c12.3
ENSDARG00000068434	-0.957533869	0.000886027	*h3f3b.1*	H3 histone, family 3B.1
ENSDARG00000063250	-0.991580254	0.047304254	*spata2*	spermatogenesis associated 2
ENSDARG00000102755	-1.008199705	0.038926557	*eml6*	EMAP like 6
ENSDARG00000104582	-1.015504681	1.93E-05	-	-
ENSDARG00000104808	-1.041822279	0.000205290	*jak2a*	Janus kinase 2a
ENSDARG00000059549	-1.052988267	0.037778287	*akap10*	A kinase (PRKA) anchor protein 10
ENSDARG00000102769	-1.068303112	0.024502864	*LOC101886358*	cadherin-18
ENSDARG00000075754	-1.070850967	0.038926557	*mri1* ●	methylthioribose-1-phosphate isomerase 1
ENSDARG00000092987	-1.084883092	0.015280802	*zmat4a*	zinc finger, matrin-type 4a
ENSDARG00000104949	-1.091630292	0.000493516	*slc27a1b*	solute carrier family 27 member 1b
ENSDARG00000018773	-1.092571836	0.037810491	*hivep2b*	human immunodeficiency virus type I enhancer binding protein 2b
ENSDARG00000086326	-1.102249238	0.048102752	-	-
ENSDARG00000058256	-1.115998371	0.003345801	*draxin*	dorsal inhibitory axon guidance protein
ENSDARG00000059150	-1.135807356	9.24E-07	*tmem107*	transmembrane protein 107
ENSDARG00000062277	-1.139804425	0.015402583	*ppp2r2cb*	protein phosphatase 2, regulatory subunit B, gamma b
ENSDARG00000052648	-1.174760386	0.038280407	*hs3st4*	heparan sulfate (glucosamine) 3-O-sulfotransferase 4
ENSDARG00000016256	-1.195331820	0.000767557	*nudt3a*	nudix (nucleoside diphosphate linked moiety X)-type motif 3a
ENSDARG00000044811	-1.222116158	2.47E-05	*cdk9* ○	cyclin-dependent kinase 9 (CDC2-related kinase)
ENSDARG00000056885	-1.225438269	0.015280802	*per1a* ■	period circadian clock 1a
ENSDARG00000011170	-1.258652500	4.89E-09	*ndrg2*	NDRG family member 2
ENSDARG00000032865	-1.295903497	1.30E-08	*pacsin1a*	protein kinase C and casein kinase substrate in neurons 1a
ENSDARG00000090232	-1.410702507	0.013667181	-	-
ENSDARG00000103030	-1.446615950	0.039448547	-	-
ENSDARG00000056871	-1.510713428	0.025145625	*ap4m1*	adaptor related protein complex 4 subunit mu 1
ENSDARG00000056683	-1.576286458	0.009342008	*cdk5* ■	cyclin-dependent kinase 5
ENSDARG00000008142	-1.754995034	0.000767557	*stxbp3*	syntaxin binding protein 3
ENSDARG00000063186	-1.894334588	0.001992783	-	si:ch211-220f12.4
ENSDARG00000105450	-2.040191320	4.23E-16	-	si:ch211-63p21.1
ENSDARG00000092208	-2.073193562	3.55E-06	-	-
ENSDARG00000102959	-2.226186138	0.014307227	-	zgc:171220
ENSDARG00000032650	-2.234388304	0.003286201	*fuk* ●	fucokinase
ENSDARG00000087762	-2.363540523	4.70E-07	*rab27b*	RAB27B, member RAS oncogene family
ENSDARG00000076299	-2.397128824	0.001954266	-	-
ENSDARG00000057321	-2.416542965	0.040667513	*tut1*	terminal uridylyl transferase 1, U6 snRNA-specific
ENSDARG00000101180	-2.581257303	2.30E-31	*mcm7* ○	minichromosome maintenance complex component 7
ENSDARG00000023299	-2.712304463	0.004114475	*snu13b*	SNU13 homolog, small nuclear ribonucleoprotein b (U4/U6.U5)
ENSDARG00000022841	-2.918793651	0.000767557	*ptk2ba*	protein tyrosine kinase 2 beta, a
ENSDARG00000032426	-2.992428438	0.008690092	*coq8ab*	coenzyme Q8A, genome duplicate b
ENSDARG00000018744	-3.014377179	0.040434955	*prl2*	prolactin 2
ENSDARG00000038475	-3.205365098	1.47E-07	*acy1*	aminoacylase 1
ENSDARG00000093803	-3.500688613	0.047304254	-	-
ENSDARG00000075301	-3.515424703	0.002890406	*gsdf*	gonadal somatic cell derived factor
ENSDARG00000102858	-3.574549768	0.045280108	-	sc:d189
ENSDARG00000037813	-3.913667855	0.003358031	-	zgc:113278
ENSDARG00000088689	-4.110502985	0.002492631	-	si:ch211-201o1.1
ENSDARG00000075433	-6.852483543	0.046936979	*myom2a*	myomesin 2a

C

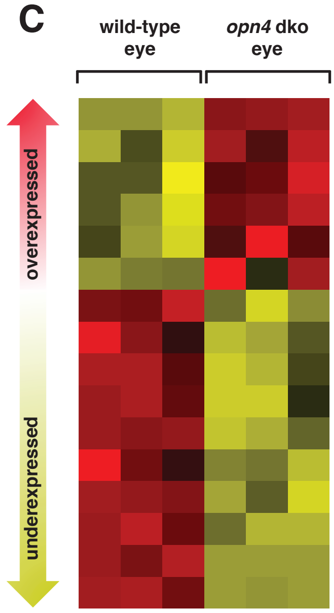

Ensembl gene ID	log2 FC	FDR *p*	Gene name	Gene description
ENSDARG00000075963	6.981133489	7.06E-96	*mhc1uba* ▲	major histocompatibility complex class I UBA
ENSDARG00000092731	4.399600875	3.05E-07	*mhc1uka* ▲	major histocompatibility complex class I UKA
ENSDARG00000059125	3.793755970	6.09E-08	*prkacbb*	protein kinase, cAMP-dependent, catalytic, beta b
ENSDARG00000001303	2.844547147	2.67E-06	*psmb8a*	proteasome subunit beta 8A
ENSDARG00000092191	2.192969929	2.06E-05	*LOC100535167*	uncharacterized LOC100535167, Gene Type: ncRNA
ENSDARG00000037140	1.772410972	0.043813899	*pfkfb1* ●	6-phosphofructo-2-kinase/fructose-2,6-biphosphatase 1
ENSDARG00000097738	-1.309274830	0.042206510	-	AL929222.2
ENSDARG00000074506	-1.677643698	0.002239404	*tmem94*	transmembrane protein 94
ENSDARG00000101984	-1.834283893	4.02E-10	*pde6gb* ●	phosphodiesterase 6G
ENSDARG00000105450	-1.990793723	5.49E-09	-	si:ch211-63p21.1
ENSDARG00000044627	-2.295374665	0.002086594	*alg8* ●	ALG8 alpha-1,3-glucosyltransferase
ENSDARG00000009386	-4.528742536	0.010565687	-	si:ch211-222n4.6
ENSDARG00000091996	-4.560888991	6.31E-07	*tcnbb*	transcobalamin beta b
ENSDARG00000098844	-4.868781347	0.011829014	-	-
ENSDARG00000026505	-7.671377409	0.041504311	-	si:ch211-285c6.3
ENSDARG00000092833	-10.07546429	5.72E-50	-	si:dkeyp-1h4.8, Gene Type: ncRNA

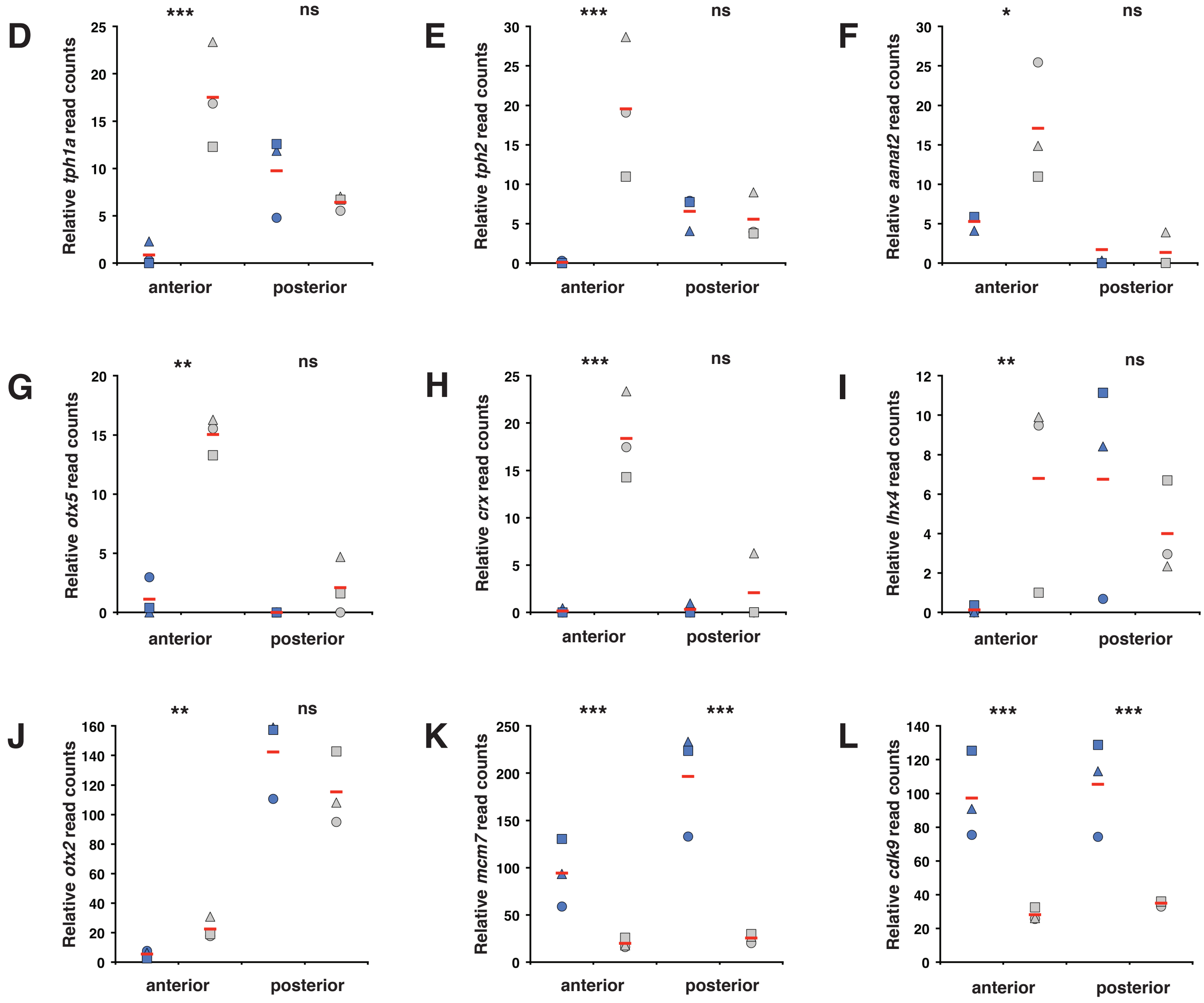

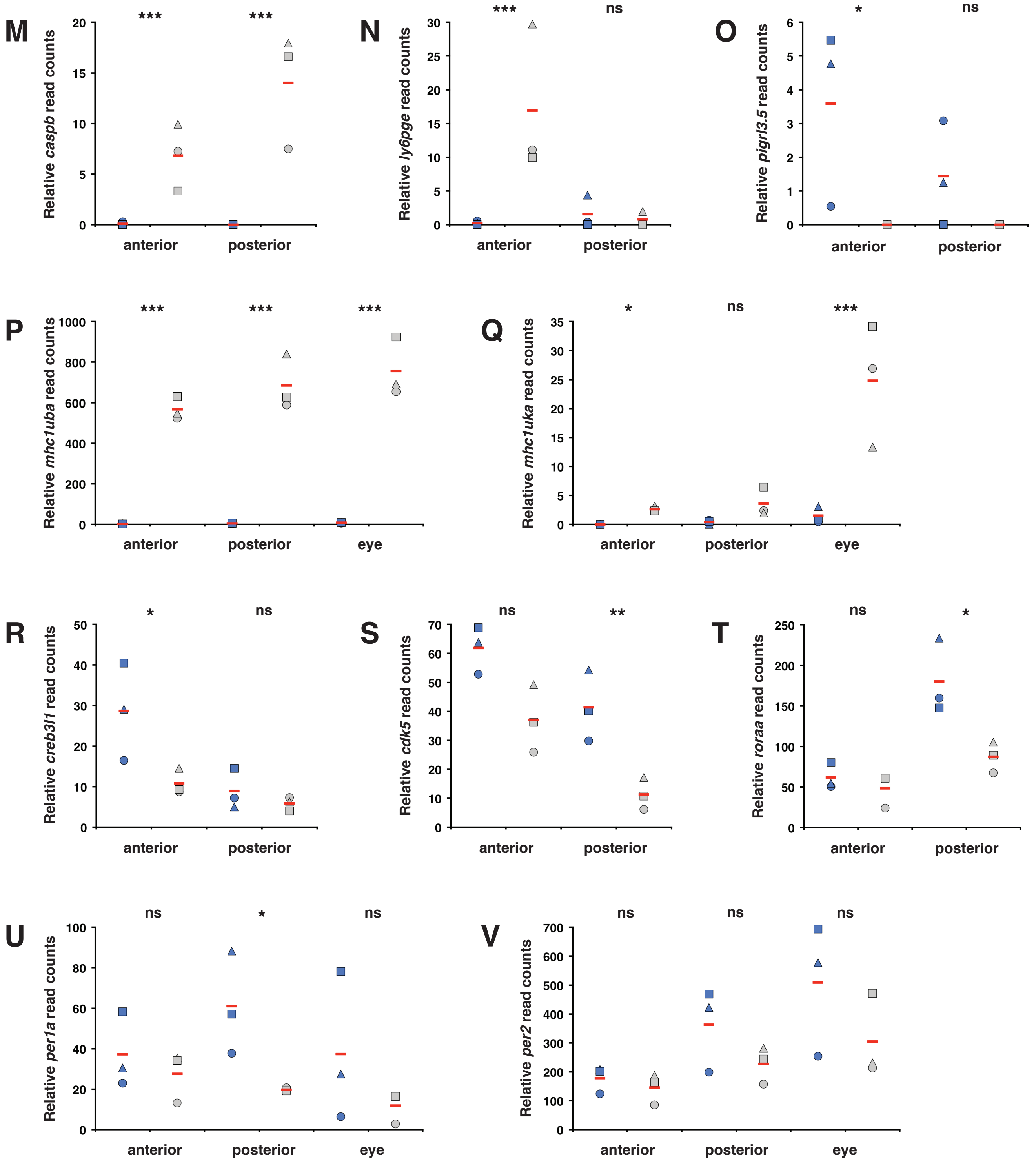
M
Relative caspb read counts
***
***
20
15
10
5
0
anterior
posterior
N
Relative ly6pge read counts
***
ns
30
25
20
15
10
5
0
anterior
posterior
O
Relative pigrl3.5 read counts
*
ns
6
5
4
3
2
1
0
anterior
posterior
P
Relative mhc1uba read counts
***
***
***
1000
800
600
400
200
0
anterior
posterior
eye
Q
Relative mhc1uka read counts
*
ns
***
35
30
25
20
15
10
5
0
anterior
posterior
eye
R
Relative creb3l1 read counts
*
ns
50
40
30
20
10
0
anterior
posterior
S
Relative cdk5 read counts
ns
**
70
60
50
40
30
20
10
0
anterior
posterior
T
Relative roraa read counts
ns
*
250
200
150
100
50
0
anterior
posterior
U
Relative per1a read counts
ns
*
ns
100
80
60
40
20
0
anterior
posterior
eye
V
Relative per2 read counts
ns
ns
ns
700
600
500
400
300
200
100
0
anterior
posterior
eye

Appendix Figure S2

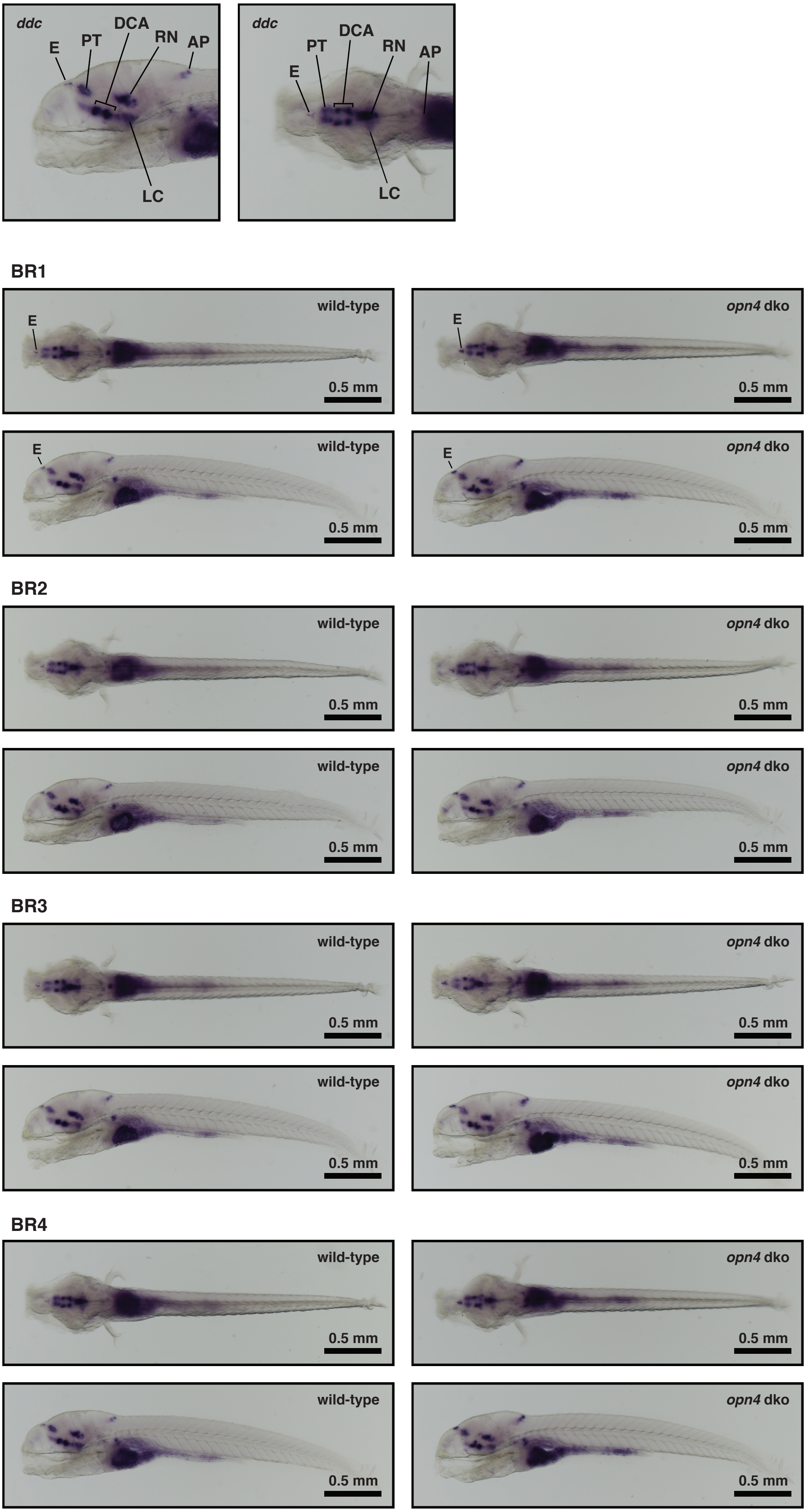

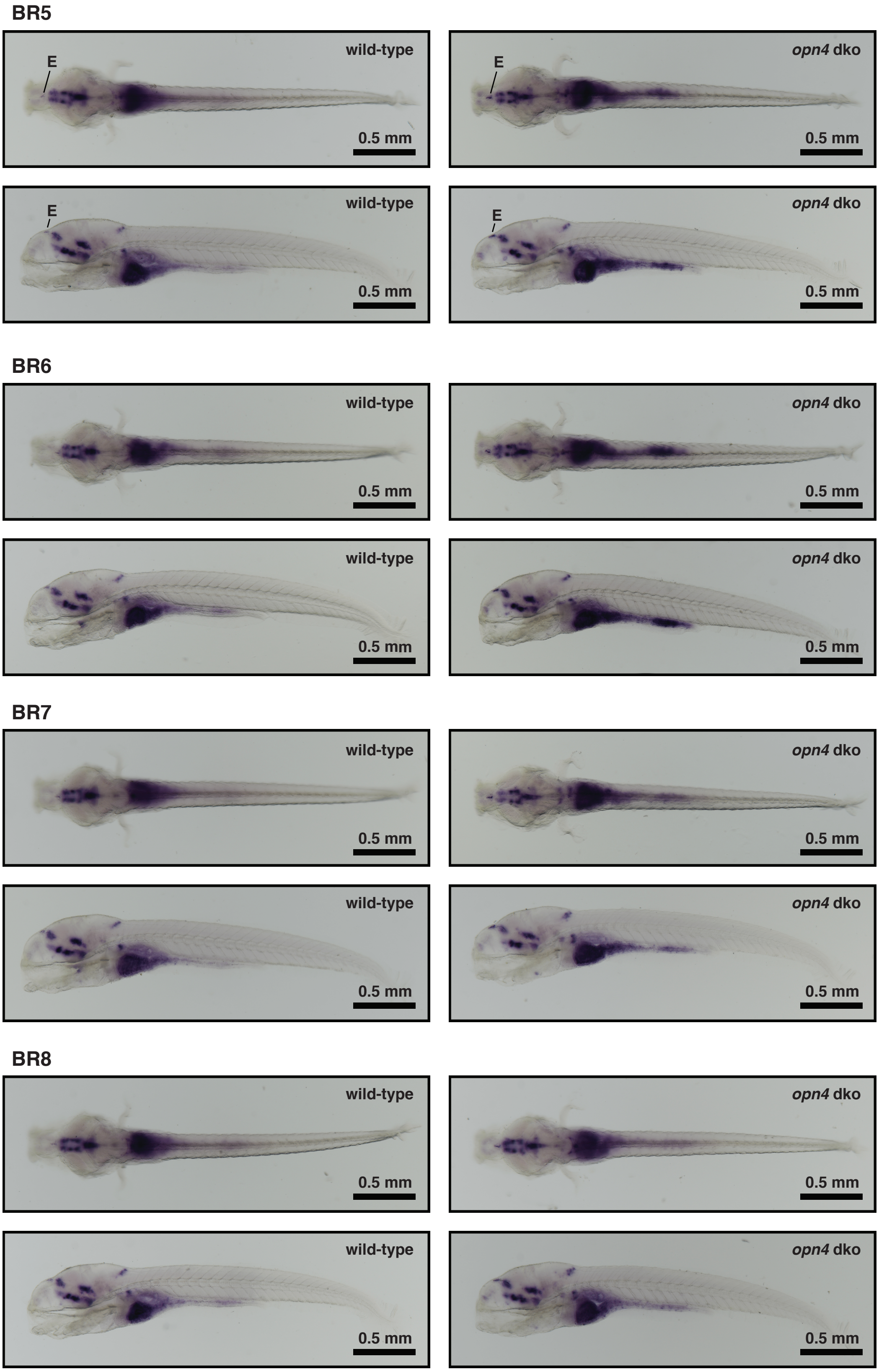
BR5
wild-type
opn4 dko
E
0.5 mm
BR6
wild-type
opn4 dko
0.5 mm
BR7
wild-type
opn4 dko
0.5 mm
BR8
wild-type
opn4 dko
0.5 mm
