# Supplementary material for: Melanopsin elevates locomotor activity during the wake state of the diurnal zebrafish
Source: EMBO Rep. 2022 Mar 1;23(5):e51528. doi: 10.15252/embr.202051528 (PMC9066073; doi:10.15252/embr.202051528)
Supplement: Supplementary file 2 — Expanded View Figures PDF [file EMBR-23-e51528-s004.pdf]

Expanded View Figures

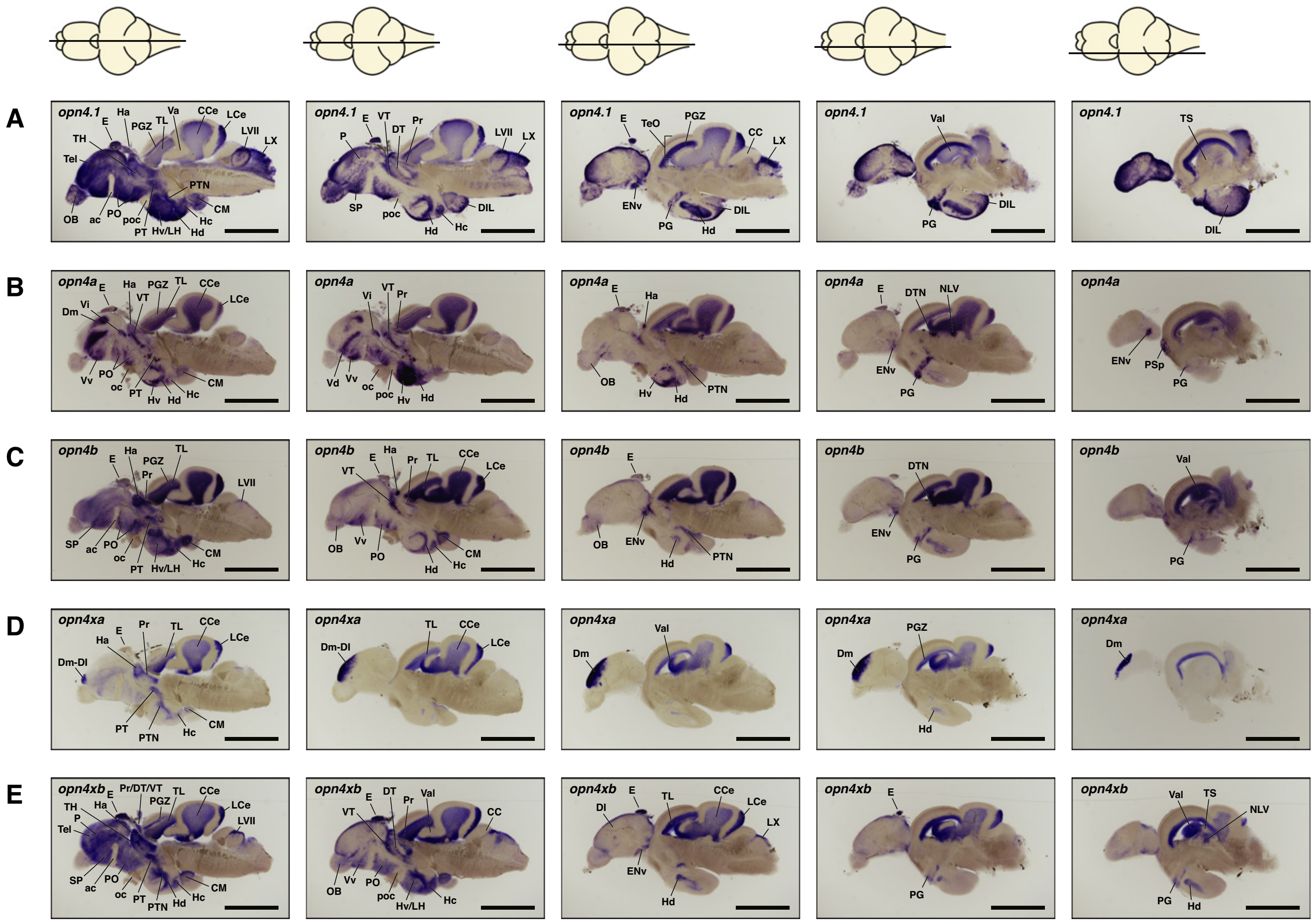

Figure EV1. *ishs* on brain sections reveal broad expression of all five zebrafish *melanopsins* in the mature brain.

(A–E) *opn4.1* (A) *opn4a* (B) *opn4b* (C) *opn4xa* (D) and *opn4xb* (E). A horizontal line through a representation of the brain, above the section, indicates the location of the section within the brain. The names and abbreviations of all brain domains in which *opn4* is expressed are listed in Table EV1. Note that the duration of the colorimetric development of the *ishs* presented in this figure is not exactly the same. Scale bar: 1.0 mm.

 © 2022 The Authors

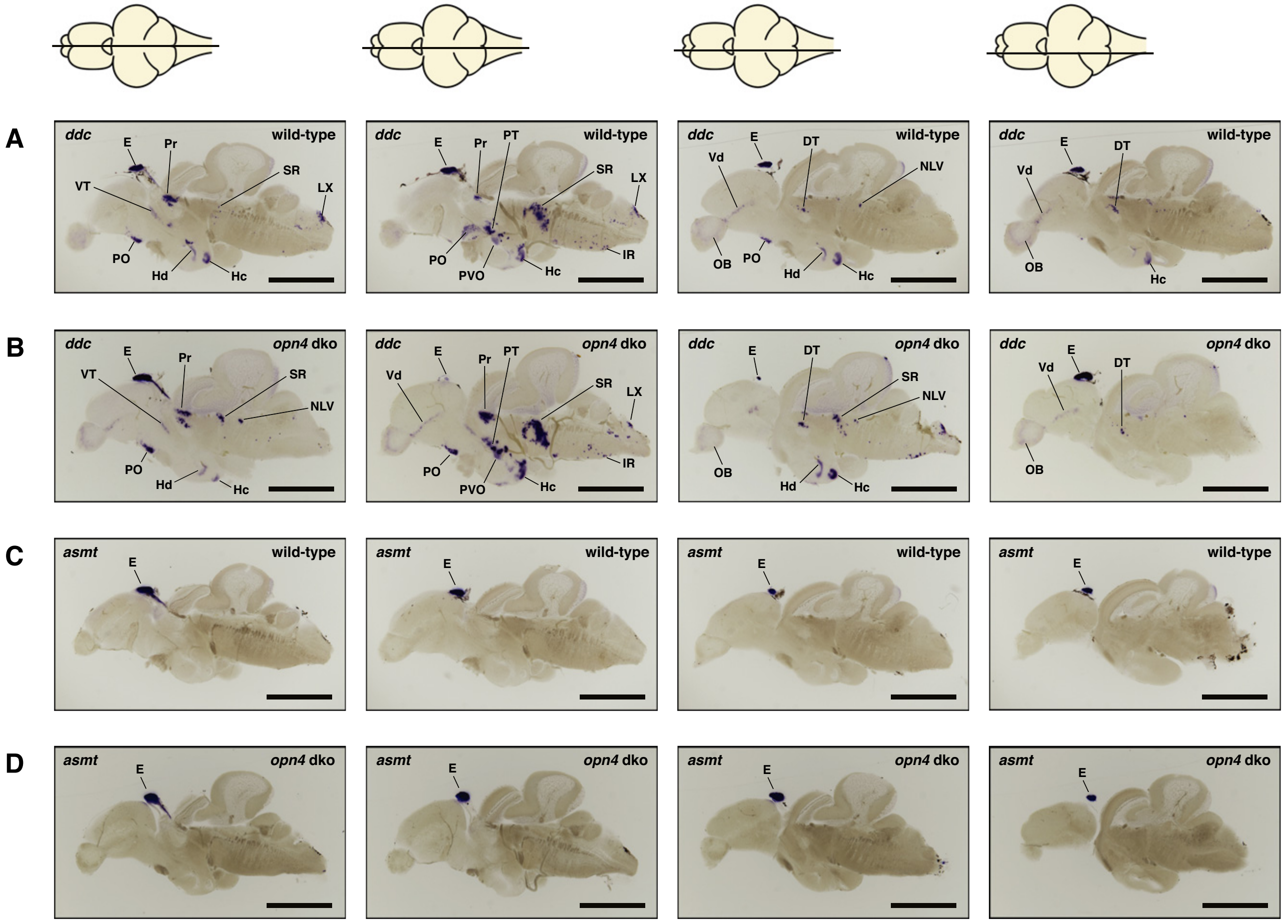

Figure EV2. ***ish* with *ddc* or *asmt* probe on *opn4* dko mature brains show wild-type expression patterns.**

A, B Same *ddc* expression pattern was observed in wild-type (A) and *opn4* dko (B) brains (pineal lost from the brain slices in (B) centre panels).

C, D *asmt* expression in wild-type (C) and *opn4* dko (D) adult brains show that *asmt* is not ectopically expressed. Thus, the higher *asmt* transcript level measured with qPCR in the *opn4* dko anterior brain can be attributed to the pineal.

Data information: The horizontal line through a representation of the brain, above the section, indicates the location of the section within the brain. Abbreviations: epiphysis cerebri (pineal) [E], caudal zone of periventricular hypothalamus [Hc], inferior raphe [IR], periventricular pretectum [Pr], preoptic area [PO], posterior tuberculum [PT], paraventricular organ [PVO], suprachiasmatic nucleus [SCN], superior raphe [SR]. Note that the duration of the colorimetric development of the *ishs* in this figure is not exactly the same. Scale bar: 1.0 mm.

© 2022 The Authors

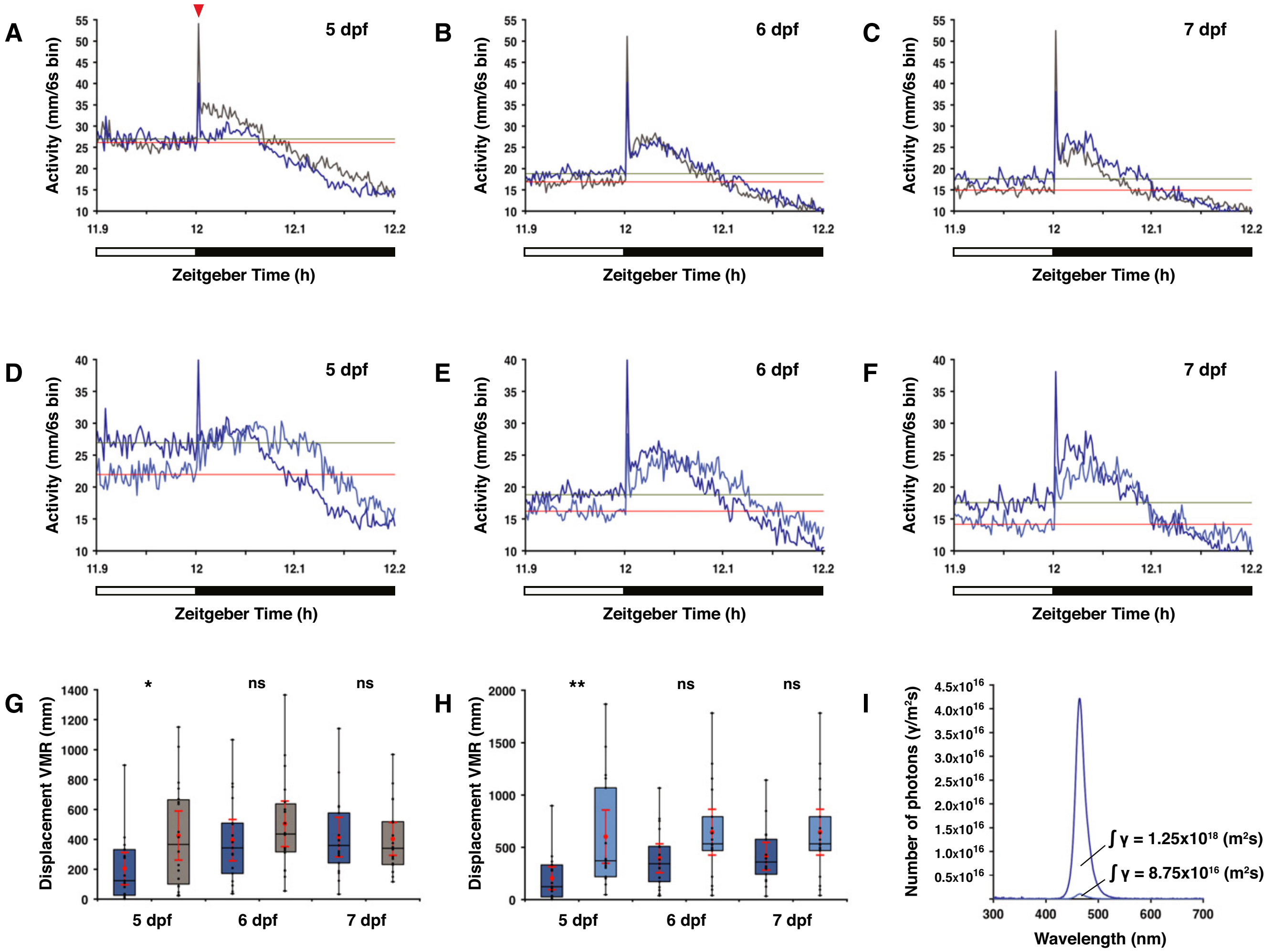

Figure EV3. Overview of visual motor response (VMR) data.

A–C Actograms show the mean activity of wild-type (dark blue line) and *opn4* dko (grey line) under a medium intensity LD 12:12 h regime at the light to dark boundary on the (A) 5th dpf, (B) 6th dpf and (C) 7th dpf. O-bend spike indicated with red arrowhead. The bar under the chart indicates the last 6 min of the light phase and the first 12 min of the dark phase. Solely for estimation, the mean activity over the last 6 min in the light phase is plotted as a horizontal line for wild-type (green) and *opn4* dko (red).

D–F Actogram shows mean activity of wild-type larvae under a medium (dark blue line) or low (light blue line) intensity LD 12:12 h regime at the light to dark boundary on the (D) 5th dpf, (E) 6th dpf and (F) 7th dpf. Solely for estimation, the mean activity over the last 6 min in the light phase is plotted as a horizontal line for larvae under medium light intensity (green) and low light intensity (red).

G *opn4* dko larvae (grey box) show similar displacement in routine turns after transition from light to darkness as wild-type (blue box) on the 6th and 7th dpf, but not on the 5th dpf (*U*-test, $P = 0.0413$). Note that displacement of each individual larva was calculated separately by comparison with its own baseline activity in the light phase. Thus the displacement of each larva on one particular day is in this case one single observation in the test statistics.

H Wild-type larvae show similar displacement in routine turns after transition from light to darkness under a low (light blue box) as under a medium (dark blue box) intensity LD regime on the 6th and 7th dpf, but not on the 5th dpf (*U*-test, $P = 0.0029$).

I Plot of the photon flux at medium (dark blue line) and low (light blue line) light intensities. For biological processes, the photon flux has a higher relevance than the irradiance (power of electromagnetic radiation) because a photoreceptor is activated by a photon. The photon flux is defined as the number of photons (γ) per unit area (m^2/s).

Data information: In (G) and (H), boxplot divides the data in quartiles: the box indicates the interquartile range, with the horizontal line in the box denoting the median of the data set, the whiskers extend to the minimum and maximum, and meet the box at the median of the lower (quartile 1) and median of the upper (quartile 3) half of the dataset. Black dots indicate biological replicates ($n = 18$), the red dot indicates the mean, red error bars indicate the 95% confidence interval and asterisks indicate significance ($0.001 < P(**) < 0.01$, $P(***) < 0.001$, ns = not significant).

 © 2022 The Authors

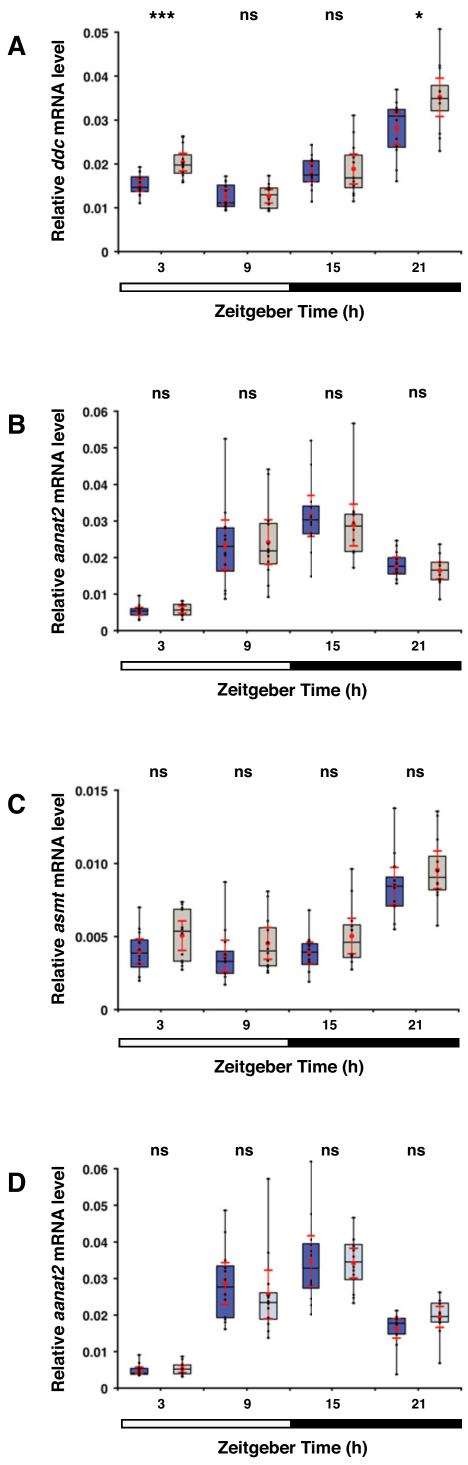

Figure EV4. Expression of genes encoding melatonin synthesis enzymes on the 6th dpf.

A Box plot shows significant higher *ddc* mRNA levels measured by qPCR in whole *opn4* dko larvae (grey box) at ZT3 and ZT21 ($P = 0.0240$) than in wild-type (blue box). Bar under the chart indicates the 12:12 h LD interval.

B As in (A) for the *aanat2* mRNA level, shows no significant difference between wild-type and *opn4* dko whole lavae.

C As in (A) for the *asmt* mRNA level, shows no significant difference between wild-type and *opn4* dko whole lavae.

D Box plot shows *aanat2* mRNA. The difference in mRNA levels between wild-type larvae under a medium (dark blue box) or low (light blue box) intensity LD regime is not significant (ZT21: *U*-test, $P = 0.0684$).

Data information: boxplot divides the data in quartiles: the box indicates the interquartile range, with the horizontal line in the box denoting the median of the data set, the whiskers extend to the minimum and maximum, and meet the box at the median of the lower (quartile 1) and median of the upper (quartile 3) half of the dataset. Black dots indicate biological replicates ($n = 12$), the red dot indicates the mean, red error bars indicate the confidence interval (95%), asterisks indicate significance ($0.001 < P(**) < 0.01$, $P(***) < 0.001$, ns = not significant).

© 2022 The Authors
