# Supplementary material for: Melanopsin elevates locomotor activity during the wake state of the diurnal zebrafish
Source: EMBO Rep. 2022 Mar 1;23(5):e51528. doi: 10.15252/embr.202051528 (PMC9066073; doi:10.15252/embr.202051528)
Supplement: Supplementary file 3 — Table EV1 [file EMBR-23-e51528-s001.doc]

###### Table EV1. *melanopsin* expression domains in the adult zebrafish brain

| **Gene Name^1^** | **Brain Domains^2^** | **Ensembl ID*** |
| --- | --- | --- |
| ***opn4.1*^*^**  (*opn4m2,*  *opn4l,*  *opn4c*) | **^*^**cerebellum (Ce: CCe?, LCe?, Val), mammillary body (CM), diffuse nucleus of the inferior lobe (DIL), epiphysis /pineal (E), entopeduncular nucleus ventral part (ENv), habenula (Ha)?, periventricular hypothalamus (H: Hc, Hd, Hv), lateral hypothalamic nucleus (LH), facial lobe (LVII), vagal lobe (LX), olfactory bulb (OB), pallium (P: Dl, Dm, Dp), preglomerular area (PG), periventricular grey zone of optic tectum (PGZ)?, preoptic area (PO: PPa PPp SC), periventricular pretectum (Pr), posterior tuberculum (PT), posterior tuberal nucleus (PTN), subpallium (SP: Vc, Vd, Vi, Vl, Vp, Vv), telencephalon (Tel), thalamus (Th: DT,VT), tuberal hypothalamus (TH), longitudinal torus (TL)? | **ENSDARG00000007553**^*^ **^*^** |
| ***opn4a*^*^**  (*opn4m1*) | **^*^**cerebellum (Ce: CCe?, LCe?), mammillary body (CM), dorsal tegmental nucleus (DTN), entopeduncular nucleus ventral part (ENv), habenula (Ha)?, periventricular hypothalamus (H: Hc, Hd, Hv), nucleus lateralis valvulae (NLV), olfactory bulb (OB: ICL), pallium (P: Dp), preglomerular area (PG), periventricular grey zone of optic tectum (PGZ)?, preoptic area (PO, SC), periventricular pretectum (Pr), parvocellular superficial pretectal nucleus (PSp), posterior tuberculum (PT), posterior tuberal nucleus (PTN), subpallium (SP: Vc, Vd, Vv, Vi), thalamus (Th: VT), longitudinal torus (TL)?, lateral torus (TLa) | **ENSDARG00000022098**^*^ |
| ***opn4b*^*^** ^*^  (*opn4m3,*  *opn4l2*) | **^*^**cerebellum (Ce: CCe?, LCe?, Val), mammillary body (CM), dorsal tegmental nucleus (DTN), entopeduncular nucleus ventral part (ENv), habenula (Ha)?, periventricular hypothalamus (H: Hc, Hd, Hv), lateral hypothalamic nucleus (LH), facial lobe (LVII), nucleus lateralis valvulae (NLV), olfactory bulb (OB: GL), pallium (P: Dc, Dl, Dm, Dp), preglomerular area (PG), periventricular grey zone of optic tectum (PGZ)?, perilemniscal nucleus (PL), preoptic area (PO: PPa, PPp, SC), periventricular pretectum (Pr), posterior tuberculum (PT: PVO, TPp), posterior tuberal nucleus (PTN), subpallium (SP: Vc, Vd, Vi, Vl, Vp, Vv), thalamus (Th: DT,VT), semicircular torus (TS), longitudinal torus (TL)?, lateral torus (TLa) | ENSDARG00000053929**^*^** |
| ***opn4xa*^*^**  (*opn4x-1*) | **^*^**cerebellum (Ce: CCe?, LCe?, Val), mammillary body (CM), entopeduncular nucleus ventral part (ENv), habenula (Ha)?, periventricular hypothalamus (H: Hc, Hd), pallium (P: Dl, Dm), preglomerular area (PG), periventricular grey zone of optic tectum (PGZ)?, periventricular pretectum (Pr), posterior tuberculum (PT: PVO, TPp), posterior tuberal nucleus (PTN), thalamus (Th: DT), longitudinal torus (TL)? | ENSDARG00000079129**^*^** |
| ***opn4xb*^*^**  (*opn4x-2,*  *opn4x1*) | **^*^**cerebellum (Ce: CCe?, LCe?, Val), mammillary body (CM), epiphysis /pineal (E), entopeduncular nucleus ventral part (ENv), habenula (Ha)?, periventricular hypothalamus (H: Hc, Hd, Hv), lateral hypothalamic nucleus (LH), facial lobe (LVII), vagal lobe (LX), nucleus lateralis valvulae (NLV), olfactory bulb (OB, ICL), pallium (P: Dl, Dm, Dp), preglomerular area (PG), periventricular grey zone of optic tectum (PGZ)?, preoptic area (PO: PPa, PPp, SC), periventricular pretectum (Pr), posterior tuberculum (PT: TPp, PVO), posterior tuberal nucleus (PTN), subpallium (SP: Vc, Vd, Vi, Vl, Vp, Vs, Vv), telencephalon (Tel), thalamus (Th: DT,VT), tuberal hypothalamus (TH), longitudinal torus (TL)?, semicircular torus (TS) | ENSDARG00000103259**^*^** |

1) Synonyms within parentheses (www.ensembl.org).

2) Terminology from Neuroanatomy of the Zebrafish Brain by Wullimann *et al*. Expression domains based on *in situs* on coronal and sagittal sections. Additional abbreviations: cerebellar corpus (CCe), central zone of dorsal telencephalic area (Dc), lateral zone of dorsal telencephalic area (Dl), medial zone of the dorsal telencephalic area (Dm), posterior zone of dorsal telencephalic area (Dp), dorsal thalamus (DT), glomerular layer of olfactory bulb (GL), inner cell layer of olfactory bulb (ICL), caudal lobe of cerebellum (LCe), parvocellular preoptic nucleus anterior part (PPa), parvocellular preoptic nucleus posterior part (PPp), paraventricular organ (PVO), suprachiasmatic nucleus (SC), periventricular nucleus of posterior tuberculum (TPp), lateral division of valvula cerebelli (Val), central nucleus of ventral telencephalic area (Vc), dorsal nucleus of ventral telencephalic area (Vd), intermediate nucleus of ventral telencephalic area (Vi), lateral nucleus of ventral telencephalic area (Vl), postcommissural nucleus of ventral telencephalic area (Vp), ventral thalamus (VT), ventral nucleus of ventral telencephalic area (Vv). ? = Background detected in sense control after prolonged colorimetric development of immunoassay. Note that *opn4a* and *opn4b* may also be expressed at low levels in the pineal, but due to moderately higher background levels in the pineal low expression therein cannot reliably be determined.
