# Supplementary material for: Melanopsin elevates locomotor activity during the wake state of the diurnal zebrafish
Source: EMBO Rep. 2022 Mar 1;23(5):e51528. doi: 10.15252/embr.202051528 (PMC9066073; doi:10.15252/embr.202051528)
Supplement: Supplementary file 4 — Table EV2 [file EMBR-23-e51528-s002.doc]

| **Ensembl ID^c^** | Gene^c^ | Endonuclease^c^ | **Forward Primer^c^** | **Reverse Primer^c^** |
| --- | --- | --- | --- | --- |
| ENSDARG00000007553 | *opn4.1* | BsgI | ggccaagctttagagtacttactga | ggctccggcagaatacatag |
| ENSDARG00000103259 | *opn4xb* | EcoNI | gcgtcaaagcatgattgttc | cactttctggaaacgatctttgc |

###### Table EV2. PCR primers and restriction endonucleases for genotyping *opn4* mutants ^1^

1) Genotyping primers were designed to amplify genomic DNA (bind intron and 5'-UTR sequences) containing the target site of the TALENs. A target site with a unique restriction recognition sequence was selected, which is lost when a mutation is introduced. The amplicon is digested with the indicated restriction endonuclease to identify the mutant.
