# Supplementary material for: Melanopsin elevates locomotor activity during the wake state of the diurnal zebrafish
Source: EMBO Rep. 2022 Mar 1;23(5):e51528. doi: 10.15252/embr.202051528 (PMC9066073; doi:10.15252/embr.202051528)
Supplement: Supplementary file 5 — Table EV3 [file EMBR-23-e51528-s007.doc]

###### Table EV3. Repeat Variable Diresidue sequences of the TALEs ^1^

| **Ensembl ID** | **Gene** | **TAL1 RVDs** | **TAL2 RVDs** |
| --- | --- | --- | --- |
| ENSDARG00000007553 | *opn4.1* | HD NI HD NG NN NG NN HD HD HD HD NG NN NN NI NN NI HD NI NG | NG HD HD HD NI NI NI NN NI NG NG HD HD NG NG NI NI NI NN |
| ENSDARG00000103259 | *opn4xb* | HD NG HD HD NI NI NG HD NG NG HD NG NG HD NI NG HD NI | NG HD NG HD HD NI NI NI HD NI NG HD HD NI HD NG |

1) TALEs that bind to the *opn4* domains presented in Table 1.
