# Supplementary material for: Melanopsin elevates locomotor activity during the wake state of the diurnal zebrafish
Source: EMBO Rep. 2022 Mar 1;23(5):e51528. doi: 10.15252/embr.202051528 (PMC9066073; doi:10.15252/embr.202051528)
Supplement: Supplementary file 6 — Table EV4 [file EMBR-23-e51528-s008.doc]

###### Table EV4. Exon junction crossing primers for quantitative PCR ^1^

| **Ensembl ID** | **Gene** | **Name** | **Forward primer** | **Tm (**°**C)** | **Reverse primer** | **Tm (**°**C)** | **Size (bp)** |
| --- | --- | --- | --- | --- | --- | --- | --- |
| ENSDARG00000079802 | *aanat2* | *arylalkylamine N-acetyltransferase 2* | ctggtggccttcatcattg | 60.1 | agagtccggcacgtgtgt | 60.3 | 81 |
| ENSDARG00000037746 | *actb1* | *actin beta 1* | tcactccccttgttcacaataa | 59.5 | ggcagcgatttcctcatc | 59.3 | 60 |
| ENSDARG00000035732 | *arntl1b* | *aryl hydrocarbon receptor nuclear translocator* | gaccgggacagtatggacac | 60.2 | gtgagcctctcttgcgttct | 59.8 | 81 |
| ENSDARG00000098249 | *asmt* | *acetylserotonin O-methyltransferase* | accagtcctgtagaccaggtg | 59.1 | gtcagaggaccagagtcatcc | 58.7 | 76 |
| ENSDARG00000011703 | *clk1a* | *clock circadian regulator a* | tcggaaactttaagtccctcaa | 60.1 | cactccctcaaagccgttt | 60.2 | 62 |
| ENSDARG00000016494 | *ddc* | *dopa decarboxylase* | gacatactgtgtggagccattg | 60.4 | gctccgtacaggcaggact | 60.4 | 67 |
| ENSDARG00000056885 | *per1a* | *period circadian clock 1a* | ggtcctcctgtgctggaac | 60.7 | ccggcaaaacattgactttt | 60.0 | 65 |
| ENSDARG00000034503 | *per2* | *period circadian clock 2* | ccaacgtggacgaagatgta | 59.6 | agcaccttctggatgtctcg | 60.4 | 64 |

1) ProbeFinder software from Roche's Universal ProbeLibrary Assay Design was used to design primers that bind to neighbouring exons spanning the exon-exon junction to avoid amplification of genomic DNA, and generate a small amplicon which increases the overall efficiency of amplification. The specificity of the qPCR was confirmed by dissociation curve analysis.
