# Supplementary material for: Melanopsin elevates locomotor activity during the wake state of the diurnal zebrafish
Source: EMBO Rep. 2022 Mar 1;23(5):e51528. doi: 10.15252/embr.202051528 (PMC9066073; doi:10.15252/embr.202051528)
Supplement: Supplementary file 8 [file EMBR-23-e51528-s005.pdf]

EMBO reports

Melanopsin elevates locomotor activity during the wake state of the diurnal zebrafish

Marcus Dekens, Bruno Fontinha, Miguel Gallach, Sandra Pflügler, and Kristin Tessmar-Raible
DOI: 10.15252/embr.202051528

Corresponding author(s): Marcus Dekens (marcus.dekens@gmail.com)

Review Timeline:

Submission Date:	16th Aug 20
Editorial Decision:	10th Sep 20
Revision Received:	10th Nov 21
Editorial Decision:	7th Jan 22
Revision Received:	24th Jan 22
Accepted:	4th Feb 22

Editor: Deniz Senyilmaz Tiebe

Transaction Report:

(Note: With the exception of the correction of typographical or spelling errors that could be a source of ambiguity, letters and reports are not edited. Depending on transfer agreements, referee reports obtained elsewhere may or may not be included in this compilation. Referee reports are anonymous unless the Referee chooses to sign their reports.)

1st Editorial Decision 10th Sep 2020

Dear Marcus,

Thank you for the submission of your research manuscript to our journal, which was now seen by two referees, whose reports are copied below.

Referees express interest in the analysis. However, they also raise overlapping important concerns that need to be addressed to consider publication here.

I find the reports informed and constructive, and believe that addressing the concerns raised will significantly strengthen the manuscript. As the reports are below, and I think all points need to be addressed, I will not detail them here.

Given these positive recommendations, we would like to invite you to revise your manuscript with the understanding that the referee concerns (as in their reports) must be fully addressed and their suggestions taken on board. Please address all referee concerns in a complete point-by-point response. Acceptance of the manuscript will depend on a positive outcome of a second round of review. It is EMBO reports policy to allow a single round of revision only and acceptance or rejection of the manuscript will therefore depend on the completeness of your responses included in the next, final version of the manuscript.

We generally allow three months as standard revision time. As a matter of policy, competing manuscripts published during this period will not negatively impact on our assessment of the conceptual advance presented by your study. However, we request that you contact the editor as soon as possible upon publication of any related work, to discuss how to proceed. Should you foresee a problem in meeting this three-month deadline, please let us know in advance and we may be able to grant an extension.

*** Temporary update to EMBO Press scooping protection policy:
We are aware that many laboratories cannot function at full efficiency during the current COVID-19/SARS-CoV-2 pandemic and have therefore extended our 'scooping protection policy' to cover the period required for a full revision to address the experimental issues highlighted in the editorial decision letter. Please contact the scientific editor handling your manuscript to discuss a revision plan should you need additional time, and also if you see a paper with related content published elsewhere.***

IMPORTANT NOTE: we perform an initial quality control of all revised manuscripts before re-review. Your manuscript will FAIL this control and the handling will be DELAYED if the following APPLIES:
1. A data availability section providing access to data deposited in public databases is missing (where applicable).
2. Your manuscript contains statistics and error bars based on n=2. Please use scatter plots in these cases.

Supplementary/additional data: The Expanded View format, which will be displayed in the main HTML of the paper in a collapsible format, has replaced the Supplementary information. You can submit up to 5 images as Expanded View. Please follow the nomenclature Figure EV1, Figure EV2 etc. The figure legend for these should be included in the main manuscript document file in a section called Expanded View Figure Legends after the main Figure Legends section. Additional Supplementary material should be supplied as a single pdf labeled Appendix. The Appendix includes a table of content on the first page with page numbers, all figures and their legends. Please follow the nomenclature Appendix Figure Sx throughout the text and also label the figures according to this nomenclature. For more details please refer to our guide to authors.

Please note that for all articles published beginning 1 July 2020, the EMBO Reports reference style will change to the Harvard style for all article types. Details and examples are provided at
https://www.embopress.org/page/journal/14693178/authorguide#referencesformat

When submitting your revised manuscript, please carefully review the instructions that follow below. Failure to include requested items will delay the evaluation of your revision.

1) a .docx formatted version of the manuscript text (including legends for main figures, EV figures and tables). Please make sure that the changes are highlighted to be clearly visible.

2) individual production quality figure files as .eps, .tif, .jpg (one file per figure).

3) a .docx formatted letter INCLUDING the reviewers' reports and your detailed point-by-point responses to their comments. As part of the EMBO Press transparent editorial process, the point-by-point response is part of the Review Process File (RPF), which will be published alongside your paper. For more details on our Transparent Editorial Process, please visit our website:
https://www.embopress.org/page/journal/14693178/authorguide#transparentprocess
You are able to opt out of this by letting the editorial office know (emboreports@embo.org). If you do opt out, the Review Process File link will point to the following statement: "No Review Process File is available with this article, as the authors have chosen not to make the review process public in this case."

4) a complete author checklist, which you can download from our author guidelines (<http://embor.embopress.org/authorguide>). Please insert information in the checklist that is also reflected in the manuscript. The completed author checklist will also be part of the RPF.

5) Please note that all corresponding authors are required to supply an ORCID ID for their name upon submission of a revised manuscript (<https://orcid.org/>). Please find instructions on how to link your ORCID ID to your account in our manuscript tracking system in our Author guidelines (<http://embor.embopress.org/authorguide>).

6) We replaced Supplementary Information with Expanded View (EV) Figures and Tables that are collapsible/expandable online. A maximum of 5 EV Figures can be typeset. EV Figures should be cited as 'Figure EV1, Figure EV2" etc... in the text and their respective legends should be included in the main text after the legends of regular figures.

- For the figures that you do NOT wish to display as Expanded View figures, they should be bundled together with their legends in a single PDF file called *Appendix*, which should start with a short Table of Content. Appendix figures should be referred to in the main text as: "Appendix Figure S1, Appendix Figure S2" etc. See detailed instructions regarding expanded view here: <http://embor.embopress.org/authorguide#expandedview>.

- Additional Tables/Datasets should be labeled and referred to as Table EV1, Dataset EV1, etc. Legends have to be provided in a separate tab in case of .xls files. Alternatively, the legend can be supplied as a separate text file (README) and zipped together with the Table/Dataset file.

7) We would also encourage you to include the source data for figure panels that show essential data.

Numerical data should be provided as individual .xls or .csv files (including a tab describing the data). For blots or microscopy, uncropped images should be submitted (using a zip archive if multiple images need to be supplied for one panel). Additional information on source data and instruction on how to label the files are available <http://embor.embopress.org/authorguide#sourcedata>.

8) Our journal encourages inclusion of *data citations in the reference list* to directly cite datasets that were re-used and obtained from public databases. Data citations in the article text are distinct from normal bibliographical citations and should directly link to the database records from which the data can be accessed. In the main text, data citations are formatted as follows: "Data ref: Smith et al, 2001" or "Data ref: NCBI Sequence Read Archive PRJNA342805, 2017". In the Reference list, data citations must be labeled with "[DATASET]". A data reference must provide the database name, accession number/identifiers and a resolvable link to the landing page from which the data can be accessed at the end of the reference. Further instructions are available at <http://embor.embopress.org/authorguide#datacitation>.

9) Please make sure to include a Data Availability Section before submitting your revision - if it is not applicable, make a statement that no data were deposited in a public database. Primary datasets (and computer code, where appropriate) produced in this study need to be deposited in an appropriate public database (see <http://embor.embopress.org/authorguide#dataavailability>).

Please remember to provide a reviewer password if the datasets are not yet public.

The accession numbers and database should be listed in a formal "Data Availability " section (placed after Materials & Method) that follows the model below. Please note that the Data Availability Section is restricted to new primary data that are part of this study.

Data availability

The datasets (and computer code) produced in this study are available in the following databases:

- RNA-Seq data: Gene Expression Omnibus GSE46843 (https://www.ncbi.nlm.nih.gov/geo/query/acc.cgi?acc=GSE46843)
- [data type]: [name of the resource] [accession number/identifier/doi] ([URL or identifiers.org/DATABASE:ACCESSION])

*** Note - All links should resolve to a page where the data can be accessed. ***

10) Regarding data quantification, please ensure to specify the name of the statistical test used to generate error bars and P values, the number (n) of independent experiments underlying each data point (not replicate measures of one sample), and the test used to calculate p-values in each figure legend. Discussion of statistical methodology can be reported in the materials and methods section, but figure legends should contain a basic description of n, P and the test applied.
Please note that error bars and statistical comparisons may only be applied to data obtained from at least three independent

biological replicates.
Please also include scale bars in all microscopy images.

We would also welcome the submission of cover suggestions, or motifs to be used by our Graphics Illustrator in designing a cover.

I look forward to seeing a revised version of your manuscript when it is ready. Please let me know if you have questions or comments regarding the revision.

Yours sincerely,

Deniz

Deniz Senyilmaz Tiebe, PhD
Editor
EMBO Reports

Referee #1:

Background: In mammals, melanopsin-expressing intrinsically photoreceptive retinal ganglion cells are essential for the synchronization of circadian clocks. In fact, they are the only retinal ganglion cells transmitting light information to the suprachiasmatic nucleus (SCN) in the hypothalamus. In many birds and poikilotherm vertebrates (like fish) the circadian master pacemaker resides in the pineal gland, rather than in the SCN. In contrast to the SCN, which receives light information solely via the retino-hypothalamic tract, the pineal gland is intrinsically photo-sensitive, as it contains various light receptors, including rhodopsin-like photoreceptors and melanopsins. The latter are particularly interesting, as they can act as both a photoreceptor and a photo-isomerase. Hence, melanopsins do not require another enzyme to recycle trans-retinal into 11-cis-retinal. While we have learned a lot on the function of mammalian melanopsins in nocturnal rodents during the past two decades, very little is known about these photoreceptors in diurnal poikilotherm vertebrates, such as fish.

In this study, Dekens et al. elucidated the role of Opn4.1 and Opn4xb, the two melanopsins that are most strongly expressed in the pineal gland of zebrafish, in the modulation of rest activity cycles. Using genetic loss-of-function experiments combined with the recording of spontaneous locomotor activity and transcriptome profiling (RNA-seq), they convincingly show that Opn4.1 and Opn4xb suppress the expression of genes involved in melatonin synthesis during the day. As suggested by the transcriptome analysis of wild-type and Opn4.1/Opn4xb null mice, the homeobox transcription factors CRX, OTX5, and LHX4 are candidates for mediating melanopsin-dependent expression of the genes involved in melatonin synthesis. In day-active mammals and fish melatonin is a sleep-inducing hormone that suppresses activity during the resting phase. As the pineal glands of double-knockout animals appear to produce more melatonin during the light phase, they display reduced locomotor activity during the say. Exposing wild-type zebrafish to a reduced light intensity during the day phenocopies the behavioral phenotypes of mutant animals, thereby suggesting that the melanopsin-dependent expression of melatonin involves light (as would be expected). The RNA-seq analysis also suggests that Opn4.1 and Opn4xb melanopsins affect metabolism, cell cycle progression, and immunity, but the significance of this has not been scrutinized by genetic loss-of-function experiments.

This is an interesting and original paper that, in principle, is suitable for publication in EMBO Reports. However, the study requires an extensive revision with re-review. The following points need to be addressed in the revised version.

Major issues

(1) A major problem with the paper is that the genome-wide transcriptome profiling (RNA-seq, was conducted with adults, whereas locomotor activity was determined in larvae. Why did the authors not perform behavioral studies with adults (see PMID: 9877412 for methods)? Or did I miss something? Clearly, it cannot be taken for granted that gene expression and locomotor activity rhythms are similar in larvae and adults.

(2) It may be difficult to determine circulating melatonin levels in the blood of zebrafish (and even more so in larvae). However, a simple and straightforward experiment might support the authors' hypothesis that melanopsins regulate locomotor activity via melanopsin. In 2001 Zhdanova et al. (Ref. cited, Fig. 4a) recorded a dose-response curve for the effect of melatonin on the locomotor activity of wild-type zebrafish larvae during the light phase, using melatonin concentrations between 10^-9 M and 10^-4 M. It would be interesting to repeat this experiment with wild-type- and Opn4.1/Opn4xb double knockout larvae. If the authors' conjecture were correct, one would expect that the behavior of mutant larvae (which already produce more melatonin than wild-type larvae) would be affected at lower external melatonin concentrations as compared to wild-type larvae.

Minor issues

(3) Abstract.
The abstract is very difficult to understand for non-specialists and must be rewritten. The authors should first point out that the SCN and the pineal gland harbor the central circadian pacemaker in mammals and fish, respectively. They then should mention that, while the SCN receives light information from the retina through the retino-hypothalamic tract, the pineal gland of fish is intrinsically photosensitive. Furthermore, it might be important to write that whereas the function of ipRGCs in the mammalian retina for the synchronization of the mammalian clock has been extensively studied, very little is known about the function of melanopsins expressed in zebrafish (and in particular in the pineal gland). Without these information, the statement "These observations suggest a conserved mechanism between zebrafish and diurnal mammals" is incomprehensible to readers outside the field. The claim ".....demonstrating that sustained repression of the melatonin pathway by Melanopsin through homeodomain transcription factors plays a key role in maintaining a high level of activity during the diurnal wake state" is an overstatement, as the function of the mentioned transcription factors in this process has not been scrutinized by loss-of-function studies. Hence, "demonstrated" should be replaced by "suggesting", and "we imply" should be replaced by "our transcriptome studies suggest that..."

(4) Introduction.
Page 3, first paragraph. "In nocturnal mouse" should read "In nocturnal mice". After the sentence "The indolamine melatonin is synthesized from the amino acid tryptophan in four consecutive steps by the enzymes Tryptophan hydroxylase (Tph, EC1.14.16.2), Dopa decarboxylase (Ddc, EC4.1.1.28), Arylalkylamine N-acetyltransferase (Aanat, EC2.3.1.87) and Acetylserotonin O-methyltransferase (Asmt, EC2.1.1.4) [26]" the authors should mention which of these enzymes is rate-limiting in the synthesis of melatonin. In the introduction, the authors should also refer to the massive amount of work showing that in zebrafish even peripheral organs (e.g. liver, kidney) and cultured cell lines are intrinsically photosensitive with regard to synchronizing their circadian clocks. Do these peripheral cell types express any of the five zebrafish melanopsin isoforms?

Results
(5) Page 4, first paragraph and Fig. S1. It appears that opn4a and opn4b are also expressed in the pineal gland, albeit to a lesser amount than opn4.1 and opn4xb. I am not asking for a quadruple knockout, but the possible contribution of opn4a and opn4b to the suppression of melatonin synthesis should be discussed more extensively.

(6) Page 7, first paragraph, Fig. S3. The FISH data for ddc expression in the brain of the double knockout animals are lacking.

(7) Page 7, second paragraph. The principle underlying the DanioVision tracking system should be briefly described.

(8) Page 9, second line. "adult data" should be replaced by "data obtained for adults".

Referee #2:

The manuscript "Melanopsin promotes locomotor activity during the wake state of the diurnal zebrafish" submitted to EMBO reports is an exciting paper that will help to elucidate the role of melanopsins that are localized to regions other than retinal ganglia on regulating locomotor activity. This is novel and significant considering humans have several domains of the brain that express melanopsin. However, some assumptions about melanopsin, locomotor activity and melatonin are drawn exclusively from mRNA expression data, are overstatements and need to be further supported through the following major revisions before publication.
Major revisions:
Quantification of melatonin was not provided and was simply assumed to be induced based on qPCR data for enzymes that synthesize melatonin. Protein expression data for Ddc and Asmt in the anterior brain is requested. Quantification of melatonin in anterior brain is also requested to support the claim that melatonin is induced and responsible for reduced locomotor activity. Melatonin is heavily implicated by the RNA seq dataset and qPCR data to be controlling the locomotor activity of the zebrafish. This implication could be validated by attempting to rescue the locomotor phenotype through knocking out or knocking down genes involved in melatonin biosynthesis such as Asmt. Assaying locomotor activity in dko zebrafish that also have Asmt knocked out is requested to further validate the hypothesis that melatonin is responsible for reduced locomotor activity.
Minor revisions:
Please discuss a potential explanation as to why a difference in locomotor activity is seen in dko zebrafish larvae at 6dpf and not at 5dpf. Is there a developmental aspect to melanopsin, what changes between day 5 and day 6?
Was locomotor activity reduced in adult dko zebrafish? At 7dpf, activity in the dark phase was increased in dko fish, does this persist and become more robust with age? Please provide locomotor activity for adult zebrafish at 14 days old, the same age that fish were sacrificed for RNA sequencing. Is daily total activity different in adult zebrafish? Please provide quantification for total daily activity in adults.
Furthermore, dko adult zebrafish have reduced levels of circadian transcripts based on RNA sequencing results. Does this affect their longevity? Is the lifespan of double knockout fish the same as wild type fish? Please provide this data if available.
In the introduction, please expand on the potential molecular mechanisms of melanopsin in regulating melatonin production. Also, please introduce and make connection between homeodomain TFs and melatonin production, what are the targets of

these transcription factors that are induced in RNA seq data set?
In the methods section under RNA sequencing, it was described that the three biological replicates for each tissue were pooled for sequencing. What was the rationale behind this? Was it to attain a sufficient quantity of RNA for sequencing? Why not just use more fish and have separate replicates?
What was the integrity score for RNA? Please provide bioanalyzer results before and after RNA fragmentation.
Please review sentence structure and grammar.

Response to reviewer #1

Hereby I would like to thank the reviewer for her/his time and effort, which greatly contributed to raising the quality of this manuscript.

(Comment 1:) A major problem with the paper is that the genome-wide transcriptome profiling (RNA-seq, was conducted with adults, whereas locomotor activity was determined in larvae. Why did the authors not perform behavioral studies with adults (see PMID: 9877412 for methods)? Or did I miss something? Clearly, it cannot be taken for granted that gene expression and locomotor activity rhythms are similar in larvae and adults.

(Reply 1:) We don't see this as a problem. RNA seq was performed on adult brains, as it is impossible to remove the brain from a larva due to its small size. As the commercially available tracking systems are designed for larvae, and not suitable for studying adult fish behaviour, we analysed larvae. Thus we optimally used the advantages of the adult to screen for candidate genes downstream of Opn4 and larval stages for locomotor activity. By showing that a few key differentially expressed genes in the adult *opn4* dko anterior brain are also differentially expressed in *opn4* dko larvae at the same stage when the larvae display the phenotype we were able to draw reliable conclusions. Thus as we analyzed the larvae on the molecular as well as behaviour level, our experimental design and conclusions are reliable.

Note that the behaviour setup the reviewer refers to has to be custom made, thus one has to build aquariums with infrared beams and write software, which requires a lot of expertise, time and money. Also, the DanioVision observation chamber from Noldus, that tracks video recorded larvae produces better quality data than the outdated tracking based on an animal interrupting an infrared beam.

(C2:) It may be difficult to determine circulating melatonin levels in the blood of zebrafish (and even more so in larvae). However, a simple and straightforward experiment might support the authors' hypothesis that melanopsins regulate locomotor activity via melanopsin. In 2001 Zhdanova et al. (Ref. cited, Fig. 4a) recorded a dose-response curve for the effect of melatonin on the locomotor activity of wild-type zebrafish larvae during the light phase, using melatonin concentrations between 10^{-9} M and 10^{-4} M. It would be interesting to repeat this experiment with wild-type- and Opn4.1/Opn4xb double knockout larvae. If the authors' conjecture were correct, one would expect that the behavior of mutant larvae (which already produce more melatonin than wild-type larvae) would be affected at lower external melatonin concentrations as compared to wild-type larvae.

(R2:) I fully agree that demonstrating a higher melatonin level in the *opn4* dko would reinforce the conclusions of the study. Measuring melatonin (over a range of time points) with MS/MS from a pineal perfusion system is the only experiment that can provide a conclusive result. The here proposed experiment does not prove that melatonin levels are increased in the *opn4* dko. Two different mechanisms that control the same process could also produce an additive effect. However, even without the melatonin measurement our data is still very convincing: We show that transcripts encoding all four melatonin synthesis enzymes as well as four homeodomain transcription factors, that are known to control this pathway, are upregulated in the mature *opn4* dko brain. In addition, we show upregulation of transcripts encoding melatonin synthesis enzymes in the pineals of larvae as well as a behaviour phenotype in larvae that is consistent with the molecular data. Moreover our data is strongly supported by other studies into how melatonin synthesis is regulated in the rat pineal as well as the mouse *opn4* knockout phenotype. Together all these experiments provide evidence that allows a reliable conclusion in this manuscript.

Note that I moved on to set up SARS-CoV-2 monitoring and fully relied on my former lab to maintain the strains. Unfortunately I found out that this lab lost the strain during the lockdown. A new colony of fish would have to be established. Recreating the *opn4* double knockout would require several generations of fish raising, which is under the current circumstances not feasible.

Minor issues:

(C3:) Abstract.
The abstract is very difficult to understand for non-specialists and must be rewritten. The authors should first point out that the SCN and the pineal gland harbor the central circadian pacemaker in mammals and fish, respectively. They then should mention that, while the SCN receives light information from the retina through the retino-hypothalamic tract, the pineal gland of fish is intrinsically photosensitive. Furthermore, it might be important to write that whereas the function of ipRGCs in the mammalian retina for the synchronization of the mammalian clock has been extensively studied, very little is known about the function of melanopsins expressed in zebrafish (and in particular in the pineal gland). Without this information, the statement "These observations suggest a conserved mechanism between zebrafish and diurnal mammals" is incomprehensible to readers outside the field. The claim ".....demonstrating that sustained repression of the melatonin pathway by Melanopsin through homeodomain transcription factors plays a key role in maintaining a high level of activity during the diurnal wake state" is an overstatement, as the function of the mentioned transcription factors in this process has not been scrutinized by loss-of-function studies. Hence, "demonstrated" should be replaced by "suggesting", and "we imply" should be replaced by "our transcriptome studies suggest that..."

(R3:) Thanks for pointing out how to improve the abstract, this is very useful. I have fully rewritten the abstract with these recommendations in mind. According to the dictionary "imply" (defined: suggest something as a logical consequence) has a very similar meaning as "suggest". Therefore, I believe that the use of "imply" in a statement is a careful way to draw a conclusion for which there is not a full 100% evidence. In the latter case I would use the word: "infer" (defined: deduce or conclude something from evidence and reasoning).

(C4a:) Introduction. Page 3, first paragraph. "In nocturnal mouse" should read "In nocturnal mice".

(R4a:) I have amended this text.

(C4b:) After the sentence "The indolamine melatonin is synthesized from the amino acid tryptophan in four consecutive steps by the enzymes Tryptophan hydroxylase (Tph, EC1.14.16.2), Dopa decarboxylase (Ddc, EC4.1.1.28), Arylalkylamine N-acetyltransferase (Aanat, EC2.3.1.87) and Acetylserotonin O-methyltransferase (Asmt, EC2.1.1.4) [26]" the authors should mention which of these enzymes is rate-limiting in the synthesis of melatonin.

(R4b:) The text now reads: The rate of melatonin synthesis mainly depends on AANAT. However, the melatonin level is effectively raised by inducing multiple components of the melatonin pathway.

(C4c:) In the introduction, the authors should also refer to the massive amount of work showing that in zebrafish even peripheral organs (e.g. liver, kidney) and cultured cell lines are intrinsically photosensitive with regard to synchronizing their circadian clocks. Do these peripheral cell types express any of the five zebrafish melanopsin isoforms?

(R4c:) We have not addressed this question, as it is outside of the scope of this study.

(C5:) Results
Page 4, first paragraph and Fig. S1. It appears that opn4a and opn4b are also expressed in the pineal gland, albeit to a lesser amount than opn4.1 and opn4xb. I am not asking for a quadruple knockout, but the possible contribution of opn4a and opn4b to the suppression of melatonin synthesis should be discussed more extensively.

(R5:) This point has been addressed in the footnotes of Table S1: Note that *opn4a* and *opn4b* may also be expressed at low levels in the pineal, but due to moderately higher background levels in the pineal low expression therein cannot reliably be determined. I have now also added it to the final paragraph of the discussion.

(C6:) Page 7, first paragraph, Fig. S3. The FISH data for ddc expression in the brain of the double knockout animals are lacking.

(R6:) At the request of the reviewer I have added the *in situ* for *ddc* in the *opn4* dko.

(C7:) Page 7, second paragraph. The principle underlying the DanioVision tracking system should be briefly described.

(R7:) Indeed this should be mentioned. I have added the following text: "Locomotor activity or velocity is defined as the rate of change of a larva's position in a unit time."

(C8:) Page 9, second line. "adult data" should be replaced by "data obtained for adults".

(R8:) I amended the text.

Response to reviewer #2

Hereby I would like to thank the reviewer for her/his time and effort, which contributed to raising the quality of this manuscript to a higher level.

(C1:) Quantification of melatonin was not provided and was simply assumed to be induced based on qPCR data for enzymes that synthesize melatonin. Protein expression data for Ddc and Asmt in the anterior brain is requested.

(R1:) The proposed experiment does not prove that melatonin levels are increased in the *opn4* dko. Measuring melatonin (over a range of time points) with MS/MS from a pineal perfusion system is the only experiment that can provide a conclusive result. However, even without the melatonin measurement our data is still very convincing: We show that transcripts encoding all four melatonin synthesis enzymes as well as four homeodomain transcription factors, that are known to control this pathway, are upregulated in the mature *opn4* dko brain. In addition, we show upregulation of transcripts encoding melatonin synthesis enzymes in the pineals of larvae as well as a behaviour phenotype in larvae that is consistent with the molecular data. Moreover our data is strongly supported by other studies into how melatonin synthesis is regulated in the rat pineal as well as the mouse *opn4* knockout phenotype. Together all these experiments provide evidence that allows a reliable conclusion in this manuscript.

Note that I moved on to set up SARS-CoV-2 monitoring and fully relied on my former lab to maintain the strains. Unfortunately I found out that this lab lost the strain during the lockdown. A new colony of fish would have to be established. Recreating the *opn4* double knockout would require several generations of fish raising, which is under the current circumstances not feasible.

(C2:) Melatonin is heavily implicated by the RNA seq dataset and qPCR data to be controlling the locomotor activity of the zebrafish. This implication could be validated by attempting to rescue the locomotor phenotype through knocking out or knocking down genes involved in melatonin biosynthesis such as Asmt. Assaying locomotor activity in dko zebrafish that also have Asmt knocked out is requested to further validate the hypothesis that melatonin is responsible for reduced locomotor activity.

(R2:) The reviewer requests a triple knockout of *opn4.1-opn4xb-asmt* to rescue the *opn4* dko phenotype. An interesting idea. This idea is most likely based on the assumption that Asmt only plays a role in the pineal. Asmt also plays a role in the eye, so that the suggested knockout will most likely lead to an additional phenotype that will make it difficult to interpret the data. Thus *asmt* will have to be knocked out in the pineal and not in the eye. This experiment requires an exceptional amount of work as a tissue-specific *asmt* knockout has to be created in the *opn4* double knockout. Moreover, even when such a triple knockout is generated, the removal of all functions of melatonin (in sleep, cell proliferation, growth, reproduction, appetite regulation, thermoregulation, osmoregulation and circadian clock) is more likely to produce a complex phenotype instead of rescuing the *opn4* dko phenotype.

Minor revisions:

(C3:) Please discuss a potential explanation as to why a difference in locomotor activity is seen in dko zebrafish larvae at 6dpf and not at 5dpf. Is there a developmental aspect to melanopsin, what changes between day 5 and day 6?

(R3:) Thanks for pointing this out. Much is unknown about the factors involved in the onset of locomotor activity (starting on the 4th dpf), so I can only speculate. I have now included a remark on the developmental aspect at the end of the first paragraph of the discussion: "The reported role for Exorh in the regulation of aanat2 raises the question if distinct photoreceptors could directy or via the clock control different steps of the melatonin pathway and/or if distinct photoreceptors could perform the same function at different stages of development. The appearance of the opn4 dko locomotor activity phenotype on the 6th dpf, after larvae can already adapt their locomotor activity to the light level, suggests that a developmental aspect plays a role."

(C4:) Was locomotor activity reduced in adult dko zebrafish? At 7dpf, activity in the dark phase was increased in dko fish, does this persist and become more robust with age?

(R4:) We did not investigate behaviour in adult fish, as this requires a totally different tracking setup. The currently commercially available tracking systems, such as the DanioVision setup from Noldus, which we used, has been designed for tracking larvae only. Note that the mouse *opn4* knockout shows a similar activity reversal phenotype (Mrosovsky & Hattar, 2005, J Comp Physiol A).

(C5:) Please provide locomotor activity for adult zebrafish at 14 days old, the same age that fish were sacrificed for RNA sequencing. Is daily total activity different in adult zebrafish? Please provide quantification for total daily activity in adults.

(R5:) This comment is based on a misunderstanding. Nowhere is written that adult fish are 14 days old or that RNA seq was done on 14 day old fish. Adult fish were placed for 14 days under a 12:12 hour LD regime and thereafter sacrificed in the middle of the light phase for RNA sequencing (see method section).

(C6:) Furthermore, dko adult zebrafish have reduced levels of circadian transcripts based on RNA sequencing results. Does this affect their longevity? Is the lifespan of double knockout fish the same as wild type fish? Please provide this data if available.

(R6:) We did not quantify the life span, however we did not notice an obvious higher mortality in *opn4* dko fish.

(C7:) In the introduction, please expand on the potential molecular mechanisms of melanopsin in regulating melatonin production. Also, please introduce and make connection between homeodomain TFs and melatonin production, what are the targets of these transcription factors that are induced in RNA seq data set?

(R7:) This is an excellent suggestion. I have rewritten the introduction and included these mechanisms.

(C8:) In the methods section under RNA sequencing, it was described that the three biological replicates for each tissue were pooled for sequencing. What was the rationale behind this? Was it to attain a sufficient quantity of RNA for sequencing? Why not just use more fish and have separate replicates?

(R8:) Indeed brain parts from three individuals were pooled to obtain sufficient quantities of mRNA for RNA sequencing. The main reason to process not more than 3 replicates was to reduce the RNA sequencing cost, and ethics plays a role too. Although 3 biological replicates may seem a rather low sample size, the key transcripts reveal highly significant differences (e.g. p-values: *tph2*: $p=1.89x10^{-13}$, *ddc*: $p=4.05x10^{-11}$, *asmt*: $p=4.74x10^{-38}$, *crx*: $p=2.69x10^{-13}$, *otx2*: p=0.004, *otx5*: p=0.001, *lhx4*: p=0.003), thus more biological replicates are not necessary. Note that key candidates were also validated in adults and larvae by qPCR.

(C9:) What was the integrity score for RNA? Please provide bioanalyzer results before and after RNA fragmentation.

(R9:) Performing RNA seq with suboptimal quality RNA makes no sense, as the data would not have any value. Importantly, the key candidates were validated with qPCR on adults as well as larvae, so that the conclusion is not solely based on the RNA seq data. The KEGG and FuncAssociate analysis on the RNA seq data clearly point at a defect in light regulated processes, consistent with the knockout of a photoreceptor. Furthermore, many of the differentially expressed genes from the RNA seq are confirmed by previous reports. The bioanalyzer data is in the lab journal which I left behind when I left the lab. I requested a copy of the data but have not received it.

(C10:) Please review sentence structure and grammar.

(R10:) I have fully rewritten the manuscript to improve clarity.

1st Revision - Editorial Decision **7th Jan 2022**

Dear Marcus,

Thank you for submitting your revised manuscript. It has now been seen by both of the original referees.

The referees find that the study is significantly improved during revision and recommends publication. However, referee #2 has remaining concerns. I have discussed these concerns with referee #1, and we concluded that these do not need to be addressed experimentally for publication here. Please address these concerns textually.

Moreover, I need you to address the editorial points below before I can accept the manuscript.

• Please address the remaining minor concerns of referee #1.
• As per our format requirements, in the reference list, citations should be listed in alphabetical order and then chronologically, with the authors' surnames and initials inverted; where there are more than 10 authors on a paper, 10 will be listed, followed by 'et al.'. Please see https://www.embopress.org/page/journal/14693178/authorguide#referencesformat
• We note that the panels of Fig S1 are not called out in the text.
• Please submit the figures as one file per figure.
• All figures need to be in portrait format.
• We note that Figures S2 and S6 run over 4 pages, therefore, these need to be converted to Appendix figures. Please see https://www.embopress.org/page/journal/14693178/authorguide#expandedview for more information.
• Please rename the Figure Ss as Expanded View Figures and update the callouts accordingly.
• The figure legends should come after the Reference list. Please add a heading as 'Figure Legends'.
• As per our format requirements, the EV Tables cannot be in jpg format. Please provide the EV Tables in excel or word format.
• Please remove the ORCID information from the manuscript text.
• Papers published in EMBO Reports include a 'synopsis' and 'bullet points' to further enhance discoverability. Both are displayed on the html version of the paper and are freely accessible to all readers. The synopsis includes a short standfirst summarizing the study in 1 or 2 sentences that summarize the paper and are provided by the authors and streamlined by the handling editor. I would therefore ask you to include your synopsis blurb and 3-5 bullet points listing the key experimental findings.
• In addition, please provide an image for the synopsis. This image should provide a rapid overview of the question addressed in the study but still needs to be kept fairly modest since the image size cannot exceed 550x400 pixels.

Thank you again for giving us to consider your manuscript for EMBO Reports, I look forward to your minor revision.

Kind regards,

Deniz

--
Deniz Senyilmaz Tiebe, PhD
Editor
EMBO Reports

Referee #1:

In their rebuttal letter and revised manuscript the authors addressed most of my queries appropriately. Although it is a pity that the dKO strain was lost during the Covid-19 lockdown, it would be unfair to ask the authors to re-establish this strain before publication. I believe that the manuscript is now almost fit for publication, pending on a few minor cosmetic changes listed below.

Page 3, first paragraph: "which models sequential events" sounds a bit awkward. How about "which anticipates and controls daily recurring events"?

Page 4, first paragraph: "CREB" (and other protein acronyms) should be spelled in capital letters (like AANAT in the next sentence).

Page 4, second paragraph: ".....nonetheless differences in light input and chronotype" does not sound right. How about ".....despite the differences in light input and chronotypes"?

Page 4. The last sentence "Altering the perspective of a photoreceptor that has until now solely been associated with behaviour to one that adapts diverse functions to the environment" is incomplete. I suggest "Our findings alter the perspective of a

photoreceptor that has until now solely been associated with behaviour to one that adapts diverse functions to the environment".

Page 5, second paragraph. In the sentence "....which comprises of the whole hypothalamus and pretectum, and part of the epithalamus (i.e. habenula), thalamus and posterior tuberculum" "comprises of the whole....." should read "comprises the whole...." (consist of something, but comprise something).

Page 6. In the sentence "Fisher's exact test indicates that differential expression of the same genes in the anterior and posterior brain is likely due to dependence (Fig. 2C)" "dependence" on what? Please clarify!

Page 11. Whereas the acronym CLK is used for Drosophila, CLOCK is generally used for vertebrate species. As aforementioned, protein acronyms should be spelled in capital letters throughout the paper.

Referee #2:

The corresponding author did not address two major concerns with the manuscript: (1) transcriptome analysis was done with adult animals and locomotion was done with larvae; (2) to prove that changes in the mRNA expression results in any changes in melatonin and these changes are, indeed, responsible for the changes in locomotion. Therefore, the main statement of the manuscript is not well supported. The author cannot provide more experimental data due to COVID19 pandemic. The revised manuscript is not much different from the original version. My recommendations on the manuscript cannot be significantly different from my original recommendations.

Dear Editor,

Please find below my response to comments of referee #1:

• comment 1: I followed his/her recommendation

• comment 2: I did NOT follow his/her recommendation.
Creb is not written in capital letters (CREB), as I refer here to the zebrafish protein. Note that zebrafish has different rules from mouse and human for writing gene and protein abbreviations. The zebrafish rules of spelling names is different because gene duplication took place in zebrafish so that it is not always clear if a gene is a paralog or ortholog. I therefore will keep myself to the ZFIN convention of zebrafish gene and protein names. (Examples: CREB [mouse protein], Creb [zebrafish protein], *Creb* [mouse gene]. *creb* [zebrafish gene])

• comment 3: I followed his/her recommendation

• comment 4: I followed his/her recommendation

• comment 5: I followed his/her recommendation

• comment 6: I followed in part his/her recommendation.
I suggest in the following sentence what the reason of dependence could be, but we did not further investigate it, so cannot make a clear statement here. I have altered the sentence to avoid further clarification.

• comment 7: I did NOT follow his/her recommendation. See my response to comment 2. I follow the ZFIN convention of zebrafish gene and protein name abbreviations.

Please find below my response to your requests received by email of 7-Jan-'22 :

• Citations are now in the "Harvard" style.

• All panels of the figures are called out in text.

• Figures submitted as one file per figure.

• All figures in 18 cm x 23 cm format.

• Figures S2 and S6 are now Appendix Figures 1 and 2 and start with list of contents.

• Figures renamed and call outs amended.

• Figure legends placed after reference list.

• EV Tables submitted as word file.

• ORCID info removed

• **Synopsis:**

This study proposes a correlation between the number of detected photons and locomotor activity in the diurnal wake state. Light elevates the activity level through a Melanopsin driven mechanism that represses the expression of melatonin pathway genes through homeodomain transcription factors.

Bullet points:

• Melanopsin elevates locomotor activity during the diurnal wake state.

• Sustained repression of the melatonin pathway is required to maintain a high level of locomotor activity during the diurnal wake state.

• A common Opn4/OPN4 driven mechanism between zebrafish and mammals.

• Melanopsin regulates the immediate behavioural response to loss of illumination in zebrafish.

• The light level affects locomotor activity during the wake state.

Notes from corresponding author:

• Note that I made further improvements to the text for clarity. All changes I made to the text have been made in review mode, except for figure and table call outs, for your convenience to see what has changed.

• One of the supplementary tables has become a table to be included in the text (Table1), all other supplementary tables are now EV Tables and renamed accordingly.

• Figure 2 has been reorganised, note that in panel D the genes that were not assigned to a pathway have been removed as this chart represents genes that were assigned to a pathway. The number of genes that were not assigned to a pathway has been mentioned in the figure legend.

• Concerning the comments of reviewer #2, I have already addressed both issues in my rebuttal letter and textually in the revised ms, so I do not see how to further address these. Comment 1 has no relevance as we validate the main overexpressed genes in adults also in larvae, on comment 2 we agree, therefore we have been very careful with our statements in the ms. However, melatonin data would not have added a novel aspect to the outcome of this study.

Kind regards,

Marcus Dekens

2nd Revision - Editorial Decision **4th Feb 2022**

Dear Marcus,

Thank you for submitting your revised manuscript. I have now looked at everything and all is fine. Therefore, I am very pleased to accept your manuscript for publication in EMBO Reports.

Congratulations on a nice work!

Kind regards,

Deniz
--
Deniz Senyilmaz Tiebe, PhD
Editor
EMBO Reports

--

At the end of this email I include important information about how to proceed. Please ensure that you take the time to read the information and complete and return the necessary forms to allow us to publish your manuscript as quickly as possible.

As part of the EMBO publication's Transparent Editorial Process, EMBO reports publishes online a Review Process File to accompany accepted manuscripts. As you are aware, this File will be published in conjunction with your paper and will include the referee reports, your point-by-point response and all pertinent correspondence relating to the manuscript.

If you do NOT want this File to be published, please inform the editorial office within 2 days, if you have not done so already, otherwise the File will be published by default [contact: emboreports@embo.org]. If you do opt out, the Review Process File link will point to the following statement: "No Review Process File is available with this article, as the authors have chosen not to make the review process public in this case." Please note that the author checklist will still be published even if you opt out of the transparent process.

Thank you again for your contribution to EMBO reports and congratulations on a successful publication. Please consider us again in the future for your most exciting work.

****

THINGS TO DO NOW:

You will receive proofs by e-mail approximately 2-3 weeks after all relevant files have been sent to our Production Office; you should return your corrections within 2 days of receiving the proofs.

Please inform us if there is likely to be any difficulty in reaching you at the above address at that time. Failure to meet our deadlines may result in a delay of publication, or publication without your corrections.

All further communications concerning your paper should quote reference number EMBOR-2020-51528V3 and be addressed to emboreports@wiley.com.

Should you be planning a Press Release on your article, please get in contact with emboreports@wiley.com as early as possible, in order to coordinate publication and release dates.

EMBO PRESS

YOU MUST COMPLETE ALL CELLS WITH A PINK BACKGROUND ➔

PLEASE NOTE THAT THIS CHECKLIST WILL BE PUBLISHED ALONGSIDE YOUR PAPER

Corresponding Author Name: Marcus Dekens

Journal Submitted to: EMBO Reports
Manuscript Number: EMBOR-2020-51528V2

USEFUL LINKS FOR COMPLETING THIS FORM

http://www.antibodypedia.com
http://1degreebio.org
http://www.equator-network.org/reporting-guidelines/improving-bioscience-research-repor
http://grants.nih.gov/grants/olaw/olaw.htm
http://www.mrc.ac.uk/Ourresearch/Ethicsresearchguidance/Useofanimals/index.htm
http://ClinicalTrials.gov
http://www.consort-statement.org
http://www.consort-statement.org/checklists/view/32-consort/66-title
http://www.equator-network.org/reporting-guidelines/reporting-recommendations-for-tumo
http://datadryad.org
http://figshare.com
http://www.ncbi.nlm.nih.gov/gap
http://www.ebi.ac.uk/ega
http://biomodels.net/
http://biomodels.net/miriam/
http://jjj.biochem.sun.ac.za
https://osp.od.nih.gov/biosafety-biosecurity-and-emerging-biotechnology/
http://www.selectagents.gov/

Reporting Checklist For Life Sciences Articles (Rev. June 2017)

This checklist is used to ensure good reporting standards and to improve the reproducibility of published results. These guidelines are consistent with the Principles and Guidelines for Reporting Preclinical Research issued by the NIH in 2014. Please follow the journal's authorship guidelines in preparing your manuscript.

A- Figures

1. Data

The data shown in figures should satisfy the following conditions:

- the data were obtained and processed according to the field's best practice and are presented to reflect the results of the experiments in an accurate and unbiased manner.
- figure panels include only data points, measurements or observations that can be compared to each other in a scientifically meaningful way.
- graphs include clearly labeled error bars for independent experiments and sample sizes. Unless justified, error bars should not be shown for technical replicates.
- if n< 5, the individual data points from each experiment should be plotted and any statistical test employed should be justified
- Source Data should be included to report the data underlying graphs. Please follow the guidelines set out in the author ship guidelines on Data Presentation.

2. Captions

Each figure caption should contain the following information, for each panel where they are relevant:

- a specification of the experimental system investigated (eg cell line, species name).
- the assay(s) and method(s) used to carry out the reported observations and measurements
- an explicit mention of the biological and chemical entity(ies) that are being measured.
- an explicit mention of the biological and chemical entity(ies) that are altered/varied/perturbed in a controlled manner.

- the exact sample size (n) for each experimental group/condition, given as a number, not a range;
- a description of the sample collection allowing the reader to understand whether the samples represent technical or biological replicates (including how many animals, litters, cultures, etc.).
- a statement of how many times the experiment shown was independently replicated in the laboratory.
- definitions of statistical methods and measures:
  - common tests, such as t-test (please specify whether paired vs. unpaired), simple $\chi 2$ tests, Wilcoxon and Mann-Whitney tests, can be unambiguously identified by name only, but more complex techniques should be described in the methods section;
  - are tests one-sided or two-sided?
  - are there adjustments for multiple comparisons?
  - exact statistical test results, e.g., P values = x but not P values < x;
  - definition of 'center values' as median or average;
  - definition of error bars as s.d. or s.e.m.

Any descriptions too long for the figure legend should be included in the methods section and/or with the source data.

In the pink boxes below, please ensure that the answers to the following questions are reported in the manuscript itself. Every question should be answered. If the question is not relevant to your research, please write NA (non applicable). We encourage you to include a specific subsection in the methods section for statistics, reagents, animal models and human subjects.

B- Statistics and general methods

Please fill out these boxes ➔ (Do not worry if you cannot see all your text once you press return)

1.a. How was the sample size chosen to ensure adequate power to detect a pre-specified effect size?	No prior experiments were available to deduct an expected effect size, as this is the first study to report these experiments. Moreover, at the onset of this project it was not clear if a difference between the two data sets could be expected. Thus, it was not possible to determine the sample size in advance. Significance indicates that the sample size was sufficient.
1.b. For animal studies, include a statement about sample size estimate even if no statistical methods were used.	One should use as few as possible animals, but as many as necessary to get significant results. As the sample size could not be deduced beforehand, experiments were executed with a sample size dictated by the limitations of the robots used. For instance to determine transcript levels with qPCR, 12 biological replicates of each wild-type and double mutant were processed, as under these conditions the maximum number of samples are reached for a 96 well plate. As a conclusion could be drawn based on significance no repeat experiments were executed in this case.
2. Describe inclusion/exclusion criteria if samples or animals were excluded from the analysis. Were the criteria pre-established?	A larva was excluded from an experiment when it did not develop normally, as assessed by morphology.
3. Were any steps taken to minimize the effects of subjective bias when allocating animals/samples to treatment (e.g. randomization procedure)? If yes, please describe.	See below.
For animal studies, include a statement about randomization even if no randomization was used.	Randomisation was used. For instance for each behaviour experiment offspring was selected from a different randomly chosen parent pair. Final analysis was based on 6 independent experiments conducted over a period of just under 2 years, thus including two generations. Note: For all experiments the same fish strain was used, and animals of the same age were compared.
4.a. Were any steps taken to minimize the effects of subjective bias during group allocation or/and when assessing results (e.g. blinding of the investigator)? If yes please describe.	No. Read response to question 4b.
4.b. For animal studies, include a statement about blinding even if no blinding was done	Larvae were placed in the DanioVision automatic observation chamber (Noldus), and were after several days of entrainment automatically tracked by the EthoVision software. The researcher regularly checked the robot, only to make sure that it was properly functioning. For gene expression experiments larvae were kept in a custom build synchronisation instrument for several days, and during this period the researcher regularly checked light and temperature to make sure that it was properly functioning, followed by taking and processing the samples. Thus human interference was minimised in the photobiology assays. Detection of transcript levels was performed by a qPCR robot.
5. For every figure, are statistical tests justified as appropriate?	Yes, see M&M.

Do the data meet the assumptions of the tests (e.g., normal distribution)? Describe any methods used to assess it.	The false discovery rate was applied for significance testing of the transcriptome sequencing raw data and the cutoff for differential expression was set at a significance level of α=0.05. Fisher's exact test was applied to determine if the shared differentially expressed genes between data sets are likely to be the result of dependence or coincidence. For behaviour and qPCR results, the data distribution was calculated with the Shapiro-Wilk test. For normally distributed data the Student t-test (t-distribution) was applied and for non-normally distributed data the nonparametric Wilcoxon rank-sum test/ Mann-Whitney U-test was applied. In addition to the mentioned tests applied to the behaviour data, I also calculated Cohen's effect size (d) to determine if the difference between the wild-type and double knockout data sets is due to interindividual variability or is the result of the mutant phenotype.
Is there an estimate of variation within each group of data?	Yes, the data is presented in box plots.
Is the variance similar between the groups that are being statistically compared?	Mostly it is, with a few exceptions. To determine if a significant difference (α=0.05) between the variances of two normally distributed data sets exists, the F-test was applied. When H0 is accepted (p>0.05), a t-test with equal variance was applied, and when H0 is rejected a t-test with Welch's correction was applied (unequal variance).

C- Reagents

6. To show that antibodies were profiled for use in the system under study (assay and species), provide a citation, catalog number and/or clone number, supplementary information or reference to an antibody validation profile. e.g., Antibodypedia (see link list at top right), 1DegreeBio (see link list at top right).	NA
7. Identify the source of cell lines and report if they were recently authenticated (e.g., by STR profiling) and tested for mycoplasma contamination.	NA

* for all hyperlinks, please see the table at the top right of the document

D- Animal Models

8. Report species, strain, gender, age of animals and genetic modification status where applicable. Please detail housing and husbandry conditions and the source of animals.	Danio rerio (Zebrafish), Tübingen strain, both genders, age 6 days pf - 1.5 years pf, mutations generated with TALEN genome editing technique (see M&M), husbandry: standard practise (see M&M and Zebrafish Book).
9. For experiments involving live vertebrates, include a statement of compliance with ethical regulations and identify the committee(s) approving the experiments.	NA. Experiments with live animals were non-invasive (see M&M).
10. We recommend consulting the ARRIVE guidelines (see link list at top right) (PLoS Biol. 8(6), e1000412, 2010) to ensure that other relevant aspects of animal studies are adequately reported. See author guidelines, under 'Reporting Guidelines'. See also: NIH (see link list at top right) and MRC (see link list at top right) recommendations. Please confirm compliance.	The experiments on vertebrates were executed according to Austrian (BMWFW-66.006/0012-WF/II/3b/2014) and European (Directive 2010/63/EU) legislation covering the use of animals for scientific purposes, and are described in detail in the submitted ms.

E- Human Subjects

11. Identify the committee(s) approving the study protocol.	NA
12. Include a statement confirming that informed consent was obtained from all subjects and that the experiments conformed to the principles set out in the WMA Declaration of Helsinki and the Department of Health and Human Services Belmont Report.	NA
13. For publication of patient photos, include a statement confirming that consent to publish was obtained.	NA
14. Report any restrictions on the availability (and/or on the use) of human data or samples.	NA
15. Report the clinical trial registration number (at ClinicalTrials.gov or equivalent), where applicable.	NA
16. For phase II and III randomized controlled trials, please refer to the CONSORT flow diagram (see link list at top right) and submit the CONSORT checklist (see link list at top right) with your submission. See author guidelines, under 'Reporting Guidelines'. Please confirm you have submitted this list.	NA
17. For tumor marker prognostic studies, we recommend that you follow the REMARK reporting guidelines (see link list at top right). See author guidelines, under 'Reporting Guidelines'. Please confirm you have followed these guidelines.	NA

F- Data Accessibility

18: Provide a "Data Availability" section at the end of the Materials & Methods, listing the accession codes for data generated in this study and deposited in a public database (e.g. RNA-Seq data: Gene Expression Omnibus GSE39462, Proteomics data: PRIDE PXD000208 etc.) Please refer to our author guidelines for 'Data Deposition'.<br><br>Data deposition in a public repository is mandatory for:<br>a. Protein, DNA and RNA sequences<br>b. Macromolecular structures<br>c. Crystallographic data for small molecules<br>d. Functional genomics data<br>e. Proteomics and molecular interactions	NA
19. Deposition is strongly recommended for any datasets that are central and integral to the study; please consider the journal's data policy. If no structured public repository exists for a given data type, we encourage the provision of datasets in the manuscript as a Supplementary Document (see author guidelines under 'Expanded View' or in unstructured repositories such as Dryad (see link list at top right) or Figshare (see link list at top right).	Provided generated transcriptome data in supplementary figure 2
20. Access to human clinical and genomic datasets should be provided with as few restrictions as possible while respecting ethical obligations to the patients and relevant medical and legal issues. If practically possible and compatible with the individual consent agreement used in the study, such data should be deposited in one of the major public access-controlled repositories such as dbGAP (see link list at top right) or EGA (see link list at top right).	NA
21. Computational models that are central and integral to a study should be shared without restrictions and provided in a machine-readable form. The relevant accession numbers or links should be provided. When possible, standardized format (SBML, CellML) should be used instead of scripts (e.g. MATLAB). Authors are strongly encouraged to follow the MIRIAM guidelines (see link list at top right) and deposit their model in a public database such as Biomodels (see link list at top right) or JWS Online (see link list at top right). If computer source code is provided with the paper, it should be deposited in a public repository or included in supplementary information.	NA

G- Dual use research of concern

22. Could your study fall under dual use research restrictions? Please check biosecurity documents (see link list at top right) and list of select agents and toxins (APHIS/CDC) (see link list at top right). According to our biosecurity guidelines, provide a statement only if it could.	NA
